# Sequence of the hyperplastic genome of the naturally competent *Thermus scotoductus *SA-01

**DOI:** 10.1186/1471-2164-12-577

**Published:** 2011-11-24

**Authors:** Kamini Gounder, Elzbieta Brzuszkiewicz, Heiko Liesegang, Antje Wollherr, Rolf Daniel, Gerhard Gottschalk, Oleg Reva, Benjamin Kumwenda, Malay Srivastava, Carlos Bricio, José Berenguer, Esta van Heerden, Derek Litthauer

**Affiliations:** 1 BioPAD Metagenomics Platform, Department of Microbial, Biochemical and Food Biotechnology, University of the Free State, Bloemfontein, South Africa; 2 Department of Genomic and Applied Microbiology & Göttingen Genomics Laboratory, Georg-August University Göttingen, Germany; 3 Bioinformatics and Computational Biology Unit, Department of Biochemistry, University of Pretoria, Lynnwood Road, Hillcrest, 0002 Pretoria, South Africa; 4 Centro de Biología Molecular Severo Ochoa. Universidad Autónoma de Madrid-Consejo Superior de Investigaciones Científicas. Madrid, 28049, Spain

## Abstract

**Background:**

Many strains of *Thermus *have been isolated from hot environments around the world. *Thermus scotoductus *SA-01 was isolated from fissure water collected 3.2 km below surface in a South African gold mine. The isolate is capable of dissimilatory iron reduction, growth with oxygen and nitrate as terminal electron acceptors and the ability to reduce a variety of metal ions, including gold, chromate and uranium, was demonstrated. The genomes from two different *Thermus thermophilus *strains have been completed. This paper represents the completed genome from a second *Thermus *species - *T. scotoductus*.

**Results:**

The genome of *Thermus scotoductus *SA-01 consists of a chromosome of 2,346,803 bp and a small plasmid which, together are about 11% larger than the *Thermus thermophilus *genomes. The *T. thermophilus *megaplasmid genes are part of the *T. scotoductus *chromosome and extensive rearrangement, deletion of nonessential genes and acquisition of gene islands have occurred, leading to a loss of synteny between the chromosomes of *T. scotoductus and T. thermophilus*. At least nine large inserts of which seven were identified as alien, were found, the most remarkable being a denitrification cluster and two operons relating to the metabolism of phenolics which appear to have been acquired from *Meiothermus ruber*. The majority of acquired genes are from closely related species of the Deinococcus-Thermus group, and many of the remaining genes are from microorganisms with a thermophilic or hyperthermophilic lifestyle. The natural competence of *Thermus scotoductus *was confirmed experimentally as expected as most of the proteins of the natural transformation system of *Thermus thermophilus *are present. Analysis of the metabolic capabilities revealed an extensive energy metabolism with many aerobic and anaerobic respiratory options. An abundance of sensor histidine kinases, response regulators and transporters for a wide variety of compounds are indicative of an oligotrophic lifestyle.

**Conclusions:**

The genome of *Thermus scotoductus *SA-01 shows remarkable plasticity with the loss, acquisition and rearrangement of large portions of its genome compared to *Thermus thermophilus*. Its ability to naturally take up foreign DNA has helped it adapt rapidly to a subsurface lifestyle in the presence of a dense and diverse population which acted as source of nutrients. The genome of *Thermus scotoductus *illustrates how rapid adaptation can be achieved by a highly dynamic and plastic genome.

## Background

The Witwatersrand Supergroup is a 2.9 billion year old formation of low-permeability sandstone and shale with minor volcanic units and conglomerates. The ambient temperature of the rock at levels in excess of 3 km is approximately 60°C [1,2]. These mines provide access to water emanating at levels up to 5 km [3,4]. Although approximately 300 bacterial and archaeal organisms have been identified using 16S rRNA gene sequencing, very few have been successfully cultivated. Detailed analysis of fissure water from many different sites in South African mines [5] has illustrated severe nutrient limitation which would require special adaptation by the microbial communities [6].

Hundreds of strains of *Thermus *have been isolated from various thermal environments around the world. All are thermophilic, Gram negative bacteria which grow optimally at temperatures between 65 to 70°C with no specific amino acid or vitamin requirements [7]. In 1999 Kieft *et al*. [1] described the isolation and characterization of a facultatively anaerobic *Thermus *strain from water collected 3.2 km below surface, which is capable of dissimilatory iron reduction as well as growth with oxygen and nitrate as terminal electron acceptors. The *Thermus *SA-01 strain (ATCC 700910) is closely related to *Thermus *strains NMX2, A.1 and VI-7 (previously isolated from thermal springs in New Mexico, USA and Portugal, respectively) and was identified as *T. scotoductus *[2]. Its ability to reduce chromate, gold and uranium was illustrated [8,9] and the chromate reductase was found to be an old yellow enzyme homologue [10]. In addition, a membrane associated "chromate reductase" [11] and "iron reductases" [12,13] were isolated.

The complete genome sequences of *Thermus thermophilus *strains HB8 and HB27 [14] have been determined. In both cases, a chromosome of slightly larger than 1.8 Mbp and a megaplasmid of 230 to 260 kbp were found. In addition, HB8 harbors a smaller plasmid of 9.3 kbp (Table 1). A high degree of synteny is found between the chromosomes of the two strains, except for an inversion near the origin of replication and the majority of the genes are conserved. A degree of plasticity is observed in the megaplasmids and it has been suggested that the major portion of the genes on the plasmid may be involved with a thermophilic lifestyle [15]. Recently, Ohtani and co-workers [16] demonstrated that *Thermus thermophilus *is polyploid under certain growth conditions and that this could be part of the survival strategy in thermophilic environments, where homologous recombination of chromosomes could be a mechanism of chromosome repair, as in *Deinococcus radiodurans *[17,18]. This implies a dynamic genome where rearrangement of genes occurs with high frequency. In addition to the plasticity of *Thermus *genomes, their system of uptake and horizontal exchange of DNA fragments is the most efficient reported to date [19-22]. Together, these provide a mechanism for adaptation to harsh environments.

**Table 1:** Genome features of two *T. thermophilus *strains and *T. scotoductus *SA-01

Feature	HB27	HB8	SA-01
Size in base pairs	1,894,877	1,849,742	2,346,803
Plasmid size in base pairs	232,605	256,992 + 9322	8,383
Total genome size	2,127,482	2,116,056	2,355,186
G+C content in percentage^a^	69.43 (69.15)	69.4 (69.3, 69.0)	64.9 (65.9)
Number of protein coding genes^a^	1,982 (228)	1,970 (251, 14)	2,506 (12)
Total number of genes	2,204	2,238	2,518
Conserved hypothetical genes	240 (39)	241 (36,2)	367 (3)
Hypothetical genes	64 (22)	107 (30,7)	168 (1)
Transposases^a,b^	12 (9)	16	22
tRNA genes	47	48	47
CRISPR sequences	9 + 1 candidate (8)	9 + 1 candidate (8)	3 + 1 candidate

Table adapted from [15]

^a ^values for the plasmids are given in brackets

We report here the complete genome sequence of *Thermus scotoductus *SA-01 and demonstrate how its hyperplasticity and it natural ability to acquire genes have enabled its adaptation to the subsurface.

## Results and Discussion

### General features of the *T. scotoductus *SA-01 complete chromosome sequence

**Figure 1:**
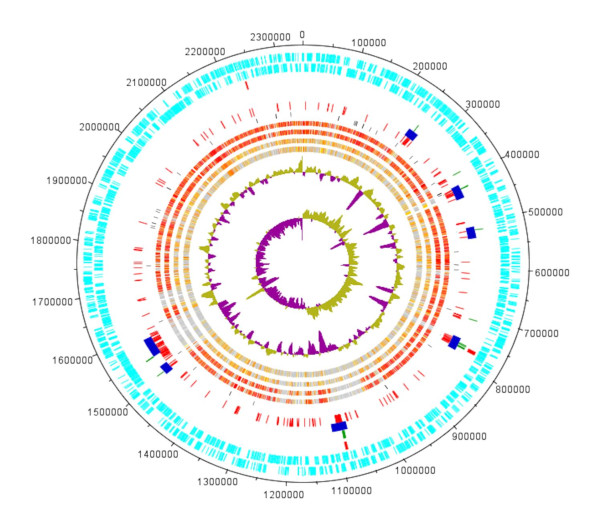
**The *Thermus scotoductus *SA-01 chromosome**. The different lanes represent (from outside): (a) CDS clockwise, (b) CDS anticlockwise, (c) Alien Genes by IslandView (Multiple Methods), (d) SIGI-HMM, (e) SeqWord sniffer, (f) Karlin's Method, (g) RNA genes, BI-BLAST comparisons for (h) *Thermus thermophilus *HB27, (i) HB8, (j) *Deinococcus radiodurans *(k) Candidatus *Desulfouridis audaxviator *, (l) G+C (m) G+C skew. BI-BLAST Sequence identities are colour coded from yellow (20%) to red (>90%). Genes missing in the comparison strain are indicated in grey.

The genome was sequenced with an approx. 20-fold coverage. The 35 contigs obtained from a combination of GS20/FLX pyrosequence runs were assembled with additional Sanger sequencing to close the gaps between the contigs. The genome of *T. scotoductus *SA-01 consists of a 2,346,803 bp chromosome (TSCc) [GenBank: CP001962] and a draft plasmid sequence of 8,383 bp (pTSC8) [GenBank: CP001963] with an average G+C content of 65.9%, similar to the *T. scotoductus *SA-01 chromosome of 64.9% (Table 1) and compared to the approximately 69% of *Thermus thermophilus*. Based on GC skew analysis, the origin of replication was identified as the location of the gene *dnaA *which encodes the chromosomal replication initiation protein DnaA (Figure 1). Manual curation of automatic annotation by deleting overlapping genes and curating gene starts resulted in 2,506 CDS on the chromosome. Proteins of unknown function, hypothetical and conserved hypothetical proteins constituted 21.4% of the total compared to 16.5% and 18.9% for HB27 and HB8, respectively (Table 1).

### Genome comparisons

**Figure 2:**
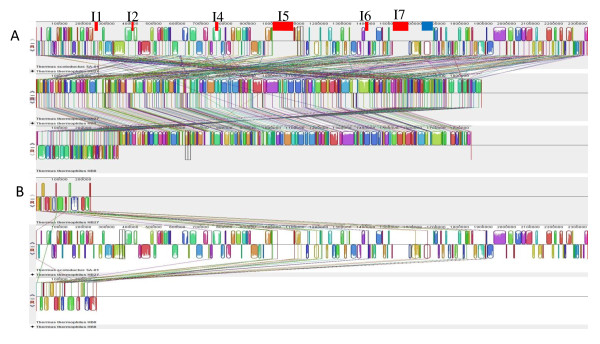
**MAUVE alignments of the *Thermus scotoductus *SA-01 chromosome with (A) *Thermus thermophilus *HB 27 (middle) and HB8 (bottom) chromosomes (B) HB27 (top) and HB8 (bottom) pTT27 plasmids**. Chromosomes and plasmids were aligned simultaneously but are shown separately for clarity. Approximate positions of inserts which were identified as putative islands are indicated as red bars, the blue bar is the largest insert which was not identified as an island. See Additional file 1, Table S1 for more detail on other inserts.

For the majority of genes present in the *T. scotoductus *SA-01 chromosome, their orthologous counterparts are found in the *T. thermophilus *chromosomes and plasmids. However, the syntenies of genes have not been preserved and the large plasmids of HB8 and HB27 contain genes, which are located on the chromosome in SA-01 (Figures 1+2). Several loci on the SA-01 chromosome are absent from the chromosomes of *T. thermophilus *(Figures 1+2). The distribution of *T. thermophilus *plasmid genes on the chromosome of *T. scotoductus *seems to be arbitrary (Figure 2). Functional analysis of the *T. thermophilus *megaplasmid genes using the Pathway Tools software showed that enzymes of several metabolic pathways are almost completely encoded by the plasmid genes, namely: coenzyme B12 synthesis and metabolism; adenosylcobalamin biosynthesis and adenosylcobalamin salvage pathways; dATP, dGTP and dUTP biosynthetic pathways; neurosporene and siroheme biosynthesis; and many other genes encoding acyl-CoA dehydrogenases, isomerases, oxidoreductase, glucosidases, galactosidases and other enzymes involved in the secondary metabolism. The plasmid genes form neat operon structures, for example the 9 genes of the adenosylcobalamin biosynthesis pathway. This is consistent with the observation that operons might be considered as the basic unit of recombinational events between species [23]. It may be hypothesized that these operons were well organized within an ancestral genome, which served as a donor for the plasmid formation in *T. thermophilus *after which an unknown genetic burst randomized the genes on the chromosomes and parts of the *T. scotoductus *chromosome were lost (Figure 2) with only 30 of the original megaplasmid genes remaining (Additional file 1, Table S1). The lost genes include the cobalamine biosynthesis pathway, plasmid stability protein and parA and parB chromosome partitioning family proteins. The *T. scotoductus *loci which were not found in the *T. thermophilus *chromosomes, might represent sequences which were either deleted from the chromosome of an ancestral organism or could be genes acquired by lateral transfer.

### Horizontally transferred genes and gene islands

The different approaches for identification of horizontally transferred genes, codon usage bias (Karlin's Codon Usage method and SIGI HMM), oligonucleotide usage patterns (SeqWord Sniffer), database comparisons (Island Pick as part of IslandView), G+C content, similarity searches or phylogenetic approaches for predicting HT genes or gene islands show varying success. For this reason we combined different approaches to try and reach a consensus.

Based upon the initial analysis of the protein coding genes of *T. scotoductus *SA-01 using Karlin's codon usage method [24], 171 (6.8%) ORFs showed codon usage differences above the threshold values and were identified as horizontally transferred candidate genes (Additional file 2, Table S2). BLAST and phylogenetic analysis showed that 80% of the HT genes showed homology to those of organisms belonging to either closely or distantly related lineage. Laterally acquired genes in *T. scotoductus *SA-01 show close homology with species belonging to diverse phyla predominantly within the Bacterial domain (Additional file 3, Table S3). Putative donor prediction by BLAST, Neighbour Joining or Maximum Likelihood prediction produced essentially the same results. The majority of the acquired genes (72%) are from species of the phylum Deinococcus-Thermus, supporting the evidence of lateral transfer from other sister *Thermus *species [25]. Furthermore, most of the horizontally acquired genes excluding the Deinococcus-Thermus were either from distantly related Proteobacteria (6%) or Firmicutes (13%) and many of the genes have been transferred from microorganisms either having hyperthermophilic (25.2%) or thermophilic (58.5%) lifestyles (Additional file 3, Table S3). The high proportion of genes from Firmicutes have been recently recognized in thermophilic bacteria, particularly in the genome sequence of *Thermosipho africanus *TCF52B, and they emerged as principal donors in thermophiles [26]. Phylogenetic analysis also identified the possible closest homolog, which was the same as the best hit in the majority of the cases (Additional file 3, Table S3).

Analysis of the *Thermus thermophilus *HB8 and HB27 genomes produced a similar number of putative HT genes, 116 (5.8%) and 104 (5.21%) on the chromosomes and 37 (14%) and 47 (21%) on the megaplasmids, respectively (Additionale file 4, Table S4 for HB8 and additional file 5, Table S5 for HB27). BLAST search of the HT genes of *T. thermophilus *HB27 against the proteins of HB8 and SA-01 was performed to determine which of the HT genes were shared (Additional file 6, Table S6). Of the HB27 sequences, 56% and 35% had homologs, while 37% and 6% of the homologs were also predicted as alien, in HB8 and SA-01 respectively. Only 43 (29%) of the HB27 sequences had homologs in both HB8 and SA-01. The HT genes of HB27 which are absent in HB8 (and mostly in SA-01) are found in small groups on the chromosome and megaplasmid. A significant fraction of the HT gene homologs of HB27 were not predicted as HT genes in HB8 and SA-01. In some cases, the gene next to the HB27 homolog in HB8 or SA-01 were predicted as HT. This could be an effect of amelioration or may reflect errors in the prediction so the results should be interpreted with caution.

In SA-01 there are many inserts not found in *T. thermophilus*, which have an alternative oligonucleotide composition compared to the chromosome. Islands I1, I2, I5, I6 and I7 (Figure 1 + 2) of the predicted genomic islands of SA-01 are inside the chromosomal loci that are absent in *T. thermophilus *genomes (Figure 2), with a part of I4 not present in *T. thermophilus*. Other strain specific regions (e.g. Island 3) in the SA-01 genome were adapted to the SA-01 sequence composition by the genome amelioration process [27].

Hypothetical proteins form the major part of the island genes, especially so for island 7. Island 1 contains predominantly enzymes involved in O-antigen synthesis, while enzymes involved in DNA processing and integration are interspersed. Island 5 hosts a variety of proteins in common with *Meiothermus ruber *DSM 1279 (accession NC_013946) including von Willebrand factor (TSC_C11350), conserved hypothetical proteins (TSC_C11340, TSC_C11360 - TSC_C11380) and two operons relating to metabolism of phenolics, TSC_C11390 - TSC_C11440 and TSC_C11450 - TSC_C11520, corresponding to MRUB_2676 - MRUB_2681 and MRUB_2682 - MRUB2689, respectively. These appear to have been directly acquired from *Meiothermus ruber *DSM 1279, the former operon having undergone an inversion in SA-01 (Additional file 7, Figure S1). The remainder of the genes in I5 have the closest orthologs in *T. thermophilus *or a variety of organisms including *Geobacter, T. aquaticus, Deinococcus deserti, Leptospirillum *or *Anaeromyxobacter dehalogenans*.

A consensus prediction of HT islands can be seen in Figure 1, with clusters of HT genes corresponding to the predicted islands. The islands are also further highlighted by anomalous GC content. The association with t-RNA genes (islands 1 and 6), a phage integrase gene associated with tRNA-Ile and followed by 4 hypothetical genes in a reverse direction to the integrase (island 2), an ATP-dependent DNA helicase PcrA and a single-stranded nucleic acid binding protein which may be parts of the former plasmid replication machinery (island 3) and an IS4 family transposase (island 7), provide clues to the possible mechanism of integration. Taken together with the comparison of the genomes of SA-01, HB8 and HB27 and their putative HT genes, this suggests that *T. scotoductus *SA-01 has a highly plastic genome and that it has adapted to its subsurface environment by the acquisition or loss of genes or gene islands.

### Transporters, Sensing and Energy Metabolism

**Figure 3:**
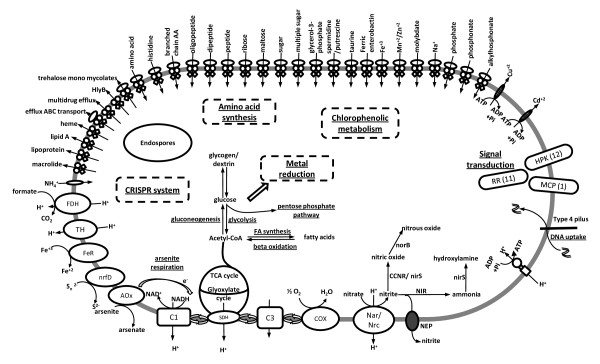
**Metabolic summary of SA-01**. FDH = formate dehydrogenase, TH = transhydrogenase, FeR = iron reductase, nrfD = polysulfide reductase, Aox = arsenite oxidase, C1 = complex 1, SDH - succinate dehydrogenase, C3 = complex 3, COX = cytochrome oxidase, NEP = nitrite extrusion protein, NIR = nitrite reductase, CCNR = copper containing nitrite reductase, RR = response regulator, MCP = methylating chemotaxis protein, HPH = histidine protein kinase.

We have identified 22 ABC transporters, including amino acid, phosphonate, phosphate, glucose, sugar, taurine, polyamine, ribose, cation, sodium-alanine symporter, trehalose mycolates, ammonium transporter, and other permeases (iron/zinc, glutathione, maltose, polypeptide, glycerol 3-phosphate, etc). Fe(III) iron import (fbpC) and two multidrug resistance proteins (Figure 3). *T. scotoductus *SA-01 retains pathways for glycolysis, gluconeogenesis, the pentose phosphate pathway, pyruvate dehydrogenase, the tricarboxylic acid cycle (TCA), glyoxylate cycle and beta oxidation (Figure 3).

ATP synthesis is accomplished by type V ATPases, which appear in a cluster of nine genes (TSC_C08720 - TSC_C08800), compared to the V/A type in HB27. A cluster of Sox genes *(soxA, soxX, soxY, soxZ) *(TSC_C21050, TSC_C 21060, TSC_C 21090, TSC_C 21100, TSC_C 21110) are also present in the genome of SA-01, located downstream of a number of cytochrome c genes (TSC_C20900 - TSC_C20950; TSC_C20970), which have a high similarity to the same proteins present in *Thermus thermophilus *HB8 and HB27 strains. The cluster of *sox *genes present in *T. thermophilus *is homologous to the *sox *genes present in sulfur-oxidizing organisms [14] and might have been horizontally transferred from an *Aquifex aeolicus *like species with some local rearrangements [25]. The presence of this *sox *operon suggests that SA-01 can oxidize sulfur compounds as a source of energy. The polysulfide reductase TSC_C03450 (annotated as molybdopterin oxidoreductase in SA-01) and NrfD (TSC_C10050) which is not present in HB27 or HB8 enables SA-01 to use S_0 _as electron acceptor [1,2]. The ability to reduce sulfur using hydrogen or organic substrates is widespread amongst thermophiles. BLAST searches showed that the closest orthologs of the TSC_C10050 are from *Thermincola *sp. JR, *Thermobaculum terrenum *ATCC BAA-798 (Yellowstone), *Thermosinus carboxydivorans *Nor1 (hot spring), *Acidiphilium cryptum *JF-5, *Anaeromyxobacter dehalogenans*, *Desulfitobacterium hafniense*. This suggests that the additional *nrfD *gene was obtained by horizontal gene transfer after separation of SA-01 from the other *Thermus *species.

The diverse metabolic capacity, abundance of sensor histidine kinases and transporters are indicative of oligotrophy, possibly in a dense and diverse population which could provide carbon and nitrogen compounds either from their metabolism or the breakdown of dead cells. Wanger *et al*. [28] illustrated that prolific, organic rich biofilms can be found at mineral surfaces in fracture zones in the deep subsurface and that these could serve as a, so far underestimated, source of C, N and P for cells in these biofilms. The absence of flagella proteins, but the presence of twitching motility proteins (TSC_C01540 and TSC_C03160), chemotaxis transducer proteins (TSC_C11960), PilT proteins TSC_C05390, TSC_C12240, TSC_C 18160, TSC_C20130), enzymes for S-layer synthesis (TSC_C19830, TSC_C24230, TSC_c03240, TSC_C16520, TSC_C24210,) and an S-layer repressor (*slrA*) (TSC_C03800), suggest a sessile lifestyle. Even in a planktonic phase, sufficient nutrients could be available in the water to sustain essential metabolic processes [6], albeit slowly [28].

### Aerobic respiration

The genome of *T. scotoductus *SA-01 encodes numerous genes assigned to a classical electron transport chain. Complex 1 NADH quinone oxidoreductases, *nuoA - nuoN *are in a cluster (TSC_C6050 - TSC_C5920), *nqo1 *(TSC_C05330), and two quinone oxidoreductases, one found on the plasmid (TSC_C14840, TSC_P80006). Complex II consists of succinate dehydrogenase (cytochrome b556 subunit SdhC (TSC_C18990), SdhA (TSC_C17730), SdhB1 and SdhB2 (TSC_C18960, TSC_C18970). SA-01 has the 2-ketoglutarate dehydrogenase complex including dihydrolipoyl dehydrogenase LpdA1 (TSC_C02350) and LpdA2 (TSC_C02700). The terminal cytochrome oxidase consists of 9 cytochrome c oxidase genes *ctaC1 *(TSC_C00920), *caaA *(TSC_C00930), *ctaH, ctaE1, ctaE2, ctaD1, coxM *(TSC_C00960 - TSC_C01000) and *ctaD2*, *ctaC2 *(TSC_C09680, TSC_C09681). Orthologous genes for the recently described complex III of HB27 (TTC1567-TTC1570) could be identified in SA-01 (TSC_C23640 - TSC_C23670) [29,30].

### Metal reduction, arsenic detoxification and respiration

Many bacteria and archaea, including SA-01 [1,2,8] display the ability to reduce metals [31]. These may be dissimilatory, detoxification or "accidental" processes. In some cases, specific proteins have been isolated and characterized e.g. the "chromate reductases" but significance of these activities remains uncertain, as it may be a secondary activity unrelated to their physiological roles [9].

Dissimilatory metal reduction is possibly the best studied in *Shewanella oneidensis *and *Geobacter. sulfurreducens *with 42 and 90 c-type cytochromes respectively, which may be indicative of their highly branched electron transfer transport systems that convey extensive versatility in terms of electron acceptor utilization [32]. In addition, Marshall *et al*. [33] showed experimentally that the c-type cytochromes of *S. oneidensis *MR-1 are essential for the reduction of U(VI) and formation of extracellular UO_2 _nanoparticles. The 12 c-type cytochromes found in SA-01 may explain its metal reducing abilities. BLAST analysis, however, indicate that none of the c-type cytochromes present in *T. scotoductus *SA-01 are similar to those in *S. oneidensis *or *G. sulfurreducens*.

Arsenite oxidase genes *aoxA *(TSC_C14700), *aoxB *(TSC_C14680) and the *arsR *transcription regulator (TSC_C14690), a hypothetical protein and a transcriptional repressor (TSC_C14720) form a cluster in SA-01. In HB8, the large and small subunit genes are found on the plasmid with the *arsR *located on the chromosome (TTHA0483). In addition SA-01 contains three more *arsR *transcriptional regulator genes; TSC_C04800 upstream of a nickel resistance protein, TSC_C14260 upstream of *hyfB,C,E,F,G*; NADH dehydrogenase (ubiquinone) iron-sulfur protein 7 and a soluble hydrogenase small subunit in a cluster and TSC_C16260 upstream of a AAA-ATPase. The arsenite oxidase converts arsenite to arsenate and is probably part of a detoxification pathway [34] but the presence of an arsenite oxidase suggests that aerobic arsenic respiration is possible by SA-01. Aerobic arsenite respiration (using arsenite as electron donor) is well known in chemolithoautotrophs [35]. The dissimilatory use of As(V) as electron acceptor by a *Thermus *isolate HR13 was described by Gihring and Banfield [36] who proposed that HR13 could oscillate between arsenite detoxification and arsenate respiration in microaerophilc culture. HR13 could however not grow with As(III) as sole energy source indicating that the As(III) oxidising system was probably not respiratory. Their cultures were however not aerated so this question is not resolved. Apart from the arsenate reductase (TSC_C23460), none of the other arsenate respiratory proteins are found in SA-01. Arsenite oxidation was also demonstrated for *Thermus aquaticus *and *Thermus thermophilus *[37]. Complete arsenic redox cycling has so far been observed only in *Thermus *[36], *Bacillus *[38] species and *Marinobacter santoriniensis *[39,40].

The ArsR family represents a class of transcriptional regulatory proteins that allowing prokaryotes to respond to stress induced by heavy metal toxicity [41]. TSC_C14260 has an ortholog in *Desulfouridis audaxviator *(e-value = 5e-11).

### Denitrification

Complete reduction of nitrate to ammonia can occur via the assimilatory nitrate reductase and cytochrome c-552 (cycA1,2,3), (TSC_C00210, TSC_C07570, TSC_C31130) and a ferredoxin-nitrite reductase (TSC_C11790). A nitrate inducible formate dehydrogenaseTSC_C10040 can also couple to the respiratory nitrate reductase to generate a proton-motive force across the cytoplasmic membrane.

**Figure 4:**
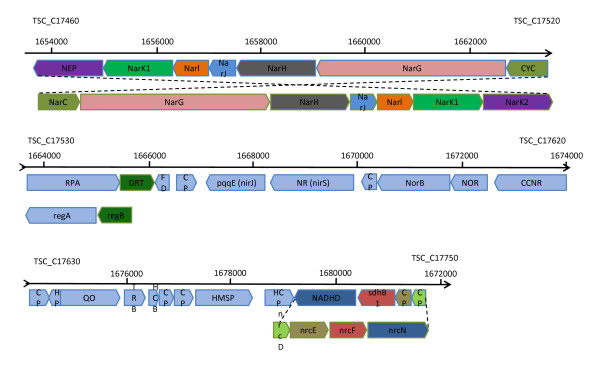
**The denitrification cluster of SA-01**. NEP = nitrite extrusion protein 1; CTC = periplasmic cytochrome C; RPA = regulatory protein A; DRT = denitrification regulator T; FD = ferredoxin 1; HP = hypothetical protein; CP = conserved hypothetical protein; NR = nitrite reductase; NOR = nitric oxide reductase cytochroms, subunit C; CCNR = copper containing nitrite reductase; QO = heme/copper-type cytochrome/quinol oxidase, subunit 1; TRB = transcriptional regulator, BadM/Rrf2 family; HCB = hemerythrin HHE cation binding domain protein; HMSP = hypothetical membrane spanning protein; HCP = putative hemerythrin HHE cation binding domain subfamily; NADHD = NADH dehydrogenase; sdhB1 = succinate dehydrogenase, iron-sulfur subunit.

A DNA fragment named the "nitrate respiratory conjugative element" in a different *Thermus thermophilus *strain [7,42] codes for the *nar *and *nrc *operons as well as their regulatory machinery. In *T. thermophilus*, the *nrcDEFN *operon is located upstream of the *nar *operon. The corresponding sequence in SA-01 codes for a NADH dehydrogenase, succinate dehydrogenase B subunit and two hypothetical proteins corresponding to NrcE and NrcD (TSC_C17720 - TSC_C17750) (Figure 4). The C-terminal 94 amino acids of the NrcE equivalent of SA-01, which consists of 111 amino acids, shows 87% identity with residues 270 - 363 of *T. thermophilus *NrcE which is absent in HB27 and HB8. Presumably this complex could be functional in SA-01.

Downstream of the *nrc *operon in *T. thermophilus *[7], lies the *nar *operon consisting of *narCGHJIKT*. In SA-01, the genes for a nitrite extrusion protein, *narK1, narI, narJ, narH, narG *and a periplasmic cytochrome c gene, which are not present in HB27 or HB8, form a cluster (TSC_C17460 - TSC_C17520) adjacent to regulatory protein A and a denitrification regulator (TSC_C17530 and TSC_C17540) which is the start of a large cluster of genes, completing the denitrification process up to nitrous oxide (Figures 3+4). The cluster contains ferredoxin 1, a conserved hypothetical proteins, coenzyme PQQ synthesis protein PqqE, nitrite reductase, conserved hypothetical protein, nitric oxide reductase subunit b NorB, NorC and what seems a copper containing nitrite reductase (TSC_C17530 - TSC_C17620), transcriptional regulator, cation binding domain protein, NADH dehydrogenase and succinate dehydrogenase b subunit (Figure 4). A ferredoxin nitrite reductase (TSC_C11790) is also found in HB27 and HB8. The presence of both a cytochrome cd1 and a copper containing nitrite reductases in the same organism is exceptional. Distant orthologs of the NarK1 protein (only in HB27), the alpha and beta subunit of nitrate reductase and the periplasmic cytochrome are found in HB27 and HB8. BLAST searches, however, indicated that the closest orthologs of all the other genes, except for ferredoxin 1 (TSC_C17750) which is found in HB27 and HB8, were from *Thermus thermophilus *[7] (all except copper containing nitrite reductase and hypothetical protein TSC_C17590), *Meiothermus silvanus *DSM 9946, *Haloferax volcanii *DS2, *Persephonella marina *EX-H1, *Hydrogenobacter thermophilus *TK-6 and *Thioalkalivibrio *sp. K90mix (Additional file 1, Table S1). The entire segment of DNA therefore appears to have been acquired by the *Thermus thermophilus *and *Thermus scotoductus *SA-01 from a distant donor/s. These genes were however not identified as acquired by any of the methods used. Balkwill *et al *[2] showed that *T. scotoductus *SA-01 could grow using nitrate as terminal electron acceptor.

### DNA transformation

Natural competence (the ability to take up and process exogenous DNA in specific growth conditions) has been observed in *T. thermophilus *strains. High frequencies of natural transformation has been displayed by this organism [43,44] and Schwarzenlander and Averhoff [21] proposed that the extraordinary broad substrate specificity of the highly efficient *T. thermophilus *HB27 DNA uptake system may contribute significantly to thermoadaptation of the organism by interdomain DNA transfer in hot environments. The natural transformation system of HB27 comprises at least 16 distinct competence proteins [45,46]. Among the 16 competence proteins the genes encoding PilM, PilN, PilO, PilW, and PilQ are found to be most essential for rendering natural transformation [20]. Several genes associated with transformation have been identified in SA-01. These include pilQ (TSC_C20070), pilM, pilN, pilO, and pilW (TSC_C20660 - TSC_C20690), pilF (TSC_C01550) pilC (TSC_C07140), pilD (TSC_C02040) comEA (TSC_C25010), comEC/rec2 (TSC_C25020), comF (TSC_C07880), comZ (TSC_C07710), dprA (TSC_C06380) and competence-damage protein CinA (TSC_C04130). In addition a FimA (TSC_C24040) and four PilT domain proteins (TSC_C05390, TSC_C12240, TSC_C18160, TSC_C20130) are present. TSC_C12240 and TSC_C20130 are absent in HB8 and HB27 while TSC_C18160 is found in HB27. PilA is absent in SA-01 and HB8, but present in HB27. Four *Thermus *species, *T. thermophilus *HB27 and HB8, *T. flavus *AT62, *T. caldophilus *GK24, and *T. aquaticus *YT1 have been demonstrated to be naturally competent, with HB27 showing the highest transformation frequency [43] with a maximal uptake rate of about 40 kb/s per cell [21] and with a broad specificity [21,22].

The transformation assays with *T. scotoductus *SA-01 cultures consistently showed colonies (20-30) with the cells incubated with the pMKNor plasmid but not with those incubated without DNA. The number of *T. thermophilus *PRQ25 transformants was tenfold more with pMKNor than those obtained for *T. scotoductus*. Therefore, we concluded that *T. scotoductus *SA1 can be transformed with plasmids carrying DNA sequences homologues to its chromosome. To confirm transformation in *T. scotoductu*s SA1, we compared the total protein pattern of the transformant colonies with that of the parental strain, and also carried out PCR amplification of the *nirK *gene, which is specific for *T. scotoductus *SA1. The results (Additional file 8, Figure S2) clearly demonstrate that transformants corresponds to *T. scotoductus *SA-01 and not to any contamination by other strain.

No putative self-replicating plasmid could be isolated by standard methods or by following a procedure for the isolation of total genomic DNA. No plasmid-like bands were detected by agarose gel electrophoresis, and transformation of *E. coli *ultra-competent cell preparations and selection on kanamycin plates was also unsuccessful. No colonies could be found which lost the kanamycin resistance after several rounds of growth without the antibiotic, whereas in *T. thermophilus *the pMK18 derivative was easily lost by this procedure [47]. Transformation with pMKNor is probably due to integration of the plasmid into the chromosome by homologous recombination.

It has been proposed [20] that the PilA 1-4 proteins may form a transformation shaft, which transports the DNA through the outer membrane into the periplasmic space. The possibility exists that the presence of PilA in HB27 and not in HB8 and SA-01 may be partly responsible for the superior transformability of HB27. Inactivation of the *Thermus *traffic ATPase PilF led to a loss of competence although the pili remained intact [45]. The *Thermus *PilF could be functionally similar to the gonococcal [48,49] and *Pseudomonas stutzeri *retraction ATPase PilT [48,50], which was demonstrated to be essential for competence. The role of the four PilT domain proteins in SA-01 needs to be elucidated. It is however evident that SA-01 possesses the machinery enabling it to take up DNA and that this may be partly responsible for its adaptation to higher temperatures.

### DNA protection and repair

The synthesis of carotenoids by HB27 and HB8, which imparts a yellow color to the cultures, has been proposed as an additional membrane and DNA protection mechanism [15,51]. The entire gene cluster TTP57-TTP67 as well as the UV endonuclease is absent in SA-01. Considering that SA-01 was isolated from water collected more than 3 kilometers underground and that it probably resided there for a significant period of time, it evidences in favor of the photo-protective role of the carotenoids.

*T. scotoductus *SA-01 has genes for repair pathways that include DNA replication, recombination and repair *recA *(TSC_C04150), *ruvB *(TSC_C19140), *uvrA *(TSC_C19840), *uvrB *(TSC_C24200), *gyrA *(TSC_C04920), *gyrB *(TSC_C22740), which were all upregulated in *Deinococcus radiodurans *after gamma radiation, but it is lacking in *katA, terB, terZ, mrsA *and *dps*, which encode for proteins alleviating oxidative stress [52]. In *Deinococcus radiodurans*, a *recA *deficient mutant was shown to be gamma ray sensitive. Wild type *recA *could complement the gamma ray sensitivity of *E. coli recA1^- ^*strain [53]. SA-01 contains the *cinA*, ligase and *recA *in a cluster (TSC_C04130-04150), as well as *recF *(TSC_C01050), *recR *(TSC_C21330) and *recO *(TSC_C13980). The RecA independent recombination repair system seems to be absent in SA-01. Of the hypothetical proteins upregulated by irradiation of *Deinococcus *[52], only DdrA showed homology to two proteins TSC_C06080 and TSC_C16120, which are Rad52/22 double-strand break repair protein in SA-01. These genes are also absent in the *Desulfouridis audaxviator *genome [6]. It seems then that the deep subsurface represents a less challenging environment, where mostly gamma radiation damage needs to be dealt with, possibly at lower levels. The RecA-dependent recombination repair system, similar to *D. radiodurans *could presumably be sufficient protection.

### Viral Resistance

Clustered regularly interspaced short palindromic repeats (CRISPR) are a distinctive feature of the genomes of most Bacteria and Archaea and are thought to be involved in resistance to bacteriophages with a resistance specificity determined by similarity between the spacers and phage sequences [54,55]. Using a CRISPRFinder online program (http://crispr.u-psud.fr/Server/CRISPRfinder.php/) on the *T. scotoductus *SA-01 chromosome revealed three CRISPR regions (CRISPR1: 575014 - 575590; CRISPR2: 1182085 - 1184314; CRISPR3: 1194463 - 1197065). Regions 2 and 3 are associated with CRISPR associated proteins. In HB27 and HB8 we find 10 and 11 CRISPRs respectively, with only two on the chromosome and the remaining 8 or 9 on the megaplasmid. The presence of an intact CRISPR system could imply that the ancestral genome may have suffered an invasion of exogenous genetic components, but that with subsequent rearrangement and deletion events most of these were lost by SA-01 or that the *T. thermophilus *faced a more phage challenging environment after separation from *T. scotoductus*.

## Conclusions

Members of the phylum Deinococcus-Thermus are known for their resistance to heat and radiation. The *Thermus *spp. are of the most widely spread [7] and have colonized a number of environments typical for themophilic organisms. It should therefore come as no surprise that a *Thermus *species should be found in the deep subsurface with ambient rock temperatures of 60°C or higher [5] at the depth where *Thermus scotoductus *SA-01 was discovered (3.2.kmbs). Fission track analysis in apatite in the Wits basin suggests that only hyperthermophiles could have existed at these depths and that current microbial communities must have migrated to these depths more recently [56]. Hyperthermophilic organisms currently found at these depths could be descendants of those initial inhabitants [56] and could have served as donors of genetic material to SA-01, assisting in thermoadaptation. The natural competence of *T. scotoductus *SA-01 could have been instrumental in the adaptation to these hot environments [19]. The earlier notion that most of the megaplasmid genes of HB27 and HB8 may be responsible for thermophily [15] may need reconsideration as SA-01 has dispensed with many of them.

The adaptability of SA-01, and *Thermus *in general, is highlighted by the extreme genome plasticity, loss of nonessential genes (e.g. carotenoid synthesis and UV endonuclease) and the acquisition of large islands subsequent to separation of SA-01 and *Thermus thermophilus*, the most significant of these being island 5 (chlorophenolic metabolism) and the insert containing the "nitrate respiratory conjugative element". SA-01 also has a number of aerobic and anaerobic respiratory options, further highlighting its metabolic versatility and its ability to sustain itself under varying conditions.

The genome of *Thermus scotoductus *SA-01 illustrates how rapid adaptation can be achieved by maintaining a highly dynamic and plastic genome. The possibility that it could sometimes be polyploid [16] has not been investigated, but it would be surprising if this was not the case.

## Methods

### Sequencing strategy

A 454 pyrosequencing/Sanger hybrid approach was used for whole genome sequencing of *T. scotoductus *SA-01. Genomic DNA from *T. scotoductus *SA-01 (ATCC 700910) was extracted from cells grown overnight at 65°C in TYG [5 g tryptone, 3 g yeast extract and 1 g glucose in 1 L dH_2_O] liquid culture, using the FastDNA^® ^SPIN Kit (Qbiogene). 454 sequencing was performed at Inqaba Biotech, Pretoria, South Africa. The methods were all standard protocols developed for the Roche GS20/FLX sequencer. The raw reads obtained were assembled into contigs using the 454 *de novo *Newbler Assembly software. A representative fosmid library of *T. scotoductus *SA-01 was constructed in *Escherichia coli *EPI300 cells using the pCC1FOS fosmid vector and the Copy Control Fosmid Library Production Kit (Epicentre Biotechnologies). Subsequently, 384 terminal DNA sequences of cloned genomic inserts were determined with an ABI 3730 × l DNA Analyzer (Applied Biosystems). Sequences were processed with Phred [57,58] and assembled with existing contigs by using the Phrap assembly tool [59]. Sequence editing of fosmid-end sequences and 454 sequences was done using GAP4 as part of the Staden software package [60]. Ambiguities and gaps were resolved using PCR and primer walking on purified DNA of selected fosmid clones.

#### 11.1.1. Gene Prediction and Annotation

The DNA sequence was submitted to the TIGR/JCVI Annotation Engine (http://www.tigr.org/AnnotationEngine), where it was run through TIGR's prokaryotic annotation pipeline. This initial automated annotation was verified and edited manually by using criteria such as the presence of a ribosome-binding site, GC frame plot analysis and similarity to known protein-encoding sequences (CDS). Functional annotation was done by using the ERGO tool from Integrated Genomics with a two-step approach. Initially, all proteins were screened against Swiss-Prot data and publicly available protein sequences from other related organisms. All predictions were verified and modified manually by comparing the protein sequences with the Pfam, GenBank, ProDom, COG, and Prosite public databases. Tmpred was used to predict transmembrane helices within CDS (http://www.ch.embnet.org/software/TMPRED_form.html).

### Comparative Genomics

The proteins obtained from the genome sequence of *T. scotoductus *SA-01 was bi-directionally blasted (A Wollherr - personal communication) against three chosen organisms using blastp from the NCBI-Blast suite. The EMBOSS package was used to evaluate reciprocal blast hits by global Needleman-Wunsch-Alignments. The entire proteome from *T. scotoductus *SA-01 has been compared to the proteins from the related species *Thermus thermophilus *HB8, *Thermus thermophilus *HB27 and the deep mine isolate *Desulfouridis auduxviator*. The global alignment of complete chromosomal sequences was performed by Mauve [61]. The Pathways Tools package [62-64] was used for reconstruction of metabolic pathways based on the genome annotation.

### Horizontally transferred genes

Several methods have been proposed so far to identify putative horizontally acquired genes. These methods are based on either deciphering atypical DNA composition or anomalous phylogenetic distribution of the genes under investigation. To get the most reliable set of candidate horizontally transferred genes in *T. scotoductus *SA-01 a combination of horizontally transferred genes detection methods/tools were applied. Horizontally transferred genomic islands were predicted by the SeqWord Genome Browser tool [65] and its semi-automatic implementation SeqWord Sniffer [66] available at http://www.bi.up.ac.za/SeqWord/ and using the IslandViewer web portal (http://www.pathogenomics.sfu.ca/islandviewer/query.php) that combines the prediction results of three genomic island identification algorithms: IslandPick [67,68], SIGI-HMM [69] and IslandPath-DIMOB [70]. Alien (horizontally transferred) genes in *T. scotoductus *SA-01, *T. thermophilus *HB8 and *T. thermophilus *HB27 were identified by Karlins's codon bias method [24] using the Predicted Highly expressed and Putative Alien genes web tool (http://www.cmbl.uga.edu/software/phxpa.html). This method is suitable for identifying highly expressed genes and also genes which have been laterally acquired [71,72]. All the putative HT gene products of SA-01 were BLASTed against the non-redundant protein database using BlastP with an e-value cut-off 1e-10 [73,74]. Paralogs were removed and the query sequence plus resultant hit sequences were used to infer the evolutionary history using the Neighbor-Joining [75] and Maximum Likelihood [76] methods with bootstrapping (1000 replicates) using MEGA5 [77]. Taxonomic and lifestyle based information of each predicted donor species was retrieved from the NCBI. The putative HT genes for *T. thermophilus *HB27 were also BLASTed against the genome sequences of *T. thermophilus *HB8 and *T. scotoductus *SA-01 to compare the putative HT genes in the three genomes.

### Natural competence

For transformation experiments *Thermus scotoductus *SA1 pre-inocula were grown overnight at 60°C in TBmq medium (8 g/L trypticase, 4g/L yeast extract, 3 g/L NaCl, MiliQ water) at half strength under aeration (180 rpm, 1/5 Erlenmeyer volume) and re-inoculated in a 1:100 ratio in the same medium. After 6 hours of incubation under the above conditions, 0.5 mL subcultures were separated into 12 mL sterile tubes to which 300 ng of plasmid DNA preparations was added. After 16 hours, the cells were plated on 1× TB prepared with carbonate-rich mineral water, and containing 30 mg/L of kanamycin. Parallel cultures without DNA were always plated as controls. Plates were incubated in a wet chamber for 48 hours at 60°C.

DNA samples used in these experiments were isolated from *E. coli *DH5α [*sup*E44, Δ(*lac*ZYA-*arg*F)U169 (Φ80 *lac*ZΔM15) *hsd*R17 *rec*A1 *end*A1 *gyr*A96 *thi *I *rel*A1]. A derivative of a bifunctional plasmid (pMK18) that replicates in both *E. coli *and *T. thermophilus *and confers thermostable resistance to kanamycin in both organisms at their respective optimal growth temperatures [78], was used. The plasmid (pMKNor) contained a 2396 bp DNA fragment encoding the *nor *genes from *T. thermophilus *PRQ25 [79,80] [GenBank: FN666415]. This sequence is very similar (87% DNA identity) to the equivalent genes of *T. scotoductus *SA1, so it was expected that homologous recombination could take place between the plasmid and the chromosome.

To confirm transformation in *T. scotoductus *SA1, we compared the total proteins pattern of the transformant colonies with that of the parental strain, and also carried out PCR amplification of the nirK gene (Primers: Kdir CCGGAGTTTTTATGTACCACTGC;Krev GGCCCCACGTTCAGGAAGTA), which is specific for *T. scotoductus *SA-01.

## Authors' contributions

All authors approved read and approved the final manuscript.

KG performed the sequencing, assembly and annotation, and wrote the first draft. EB and HL supervised the completion of the genome sequencing, assembly and annotation. AW performed and interpreted the bidirectional BLASTS. RD and GG supervised the genome sequencing. EvH contributed toward writing the manuscript. OR, BK and MS identified gene islands and alien genes and helped with the interpretation of the data. JB and CB performed the experiments to illustrate natural competence. DL provided funding, coordinated the research and wrote the final manuscript.

## Supplementary Material

Additional file 1 **Table S1. BLASTP comparisons between Thermus scotoductus SA01 and Thermus thermophilus HB27 and HB8 genomes**. This table provides a BLAST comparison between the predicted coding sequences of *Thermus scotoductus *SA-01 and *Thermus thermophiles *HB27 and HB8 and illustrates the conserved genes, the SA-01 genes which are not present in HB27 or HB8 as well as the predicted gene islands of SA-01. Click here for file

Additional file 2 **Table S2. List of alien genes: *Thermus scotoductus *SA01**. Contains a list of putative alien gens in *Thermus scotoductus *SA-01 as determined by codon bias relative to all genes using Karlin's codon bias method. Click here for file

Additional file 3 **Table S3. Putative horizontally transferred genes in the Thermus scotoductus SA-01 complete genome and their putative donor organisms based on BLAST best hits and on Neighbor-joining and Maximum likelihood trees**. Contains a list of predicted horisontally transferred genes and the putative donors organisms. Click here for file

Additional file 4 **Table S4. List of alien genes: *Thermus thermophilus *HB8 chromosome and megaplasmid**. Contains a list of putative alien gens in *Thermus thermophilusHB8 *as determined by codon bias relative to all genes using Karlin's codon bias method. Click here for file

Additional file 5 **Table S5. List of alien genes: *Thermus thermophilus *HB27 chromosome and megaplasmid**. Contains a list of putative alien gens in *Thermus thermophilusHB8 *as determined by codon bias relative to all genes using Karlin's codon bias method. Click here for file

Additional file 6 **Table S6. Putative alien genes in Thermus thermouphilus HB27 and their homologs in HB8 and SA01**. Contains a comparison of the putative horisontally transferred genes in *Thermus scotoductus *SA-01 and *Thermus thermophilus *HB27 and HB8. Click here for file

Additional file 7 **Figure S1. Part of Island 5 showing the DNA segment acquired by *T. scotoductus *SA01 from *Meiothermus ruber***. Illustrates the gene cluster for phenolic metabolism of SA-01 which was acquired from *Meiothermus ruber*. Click here for file

Additional file 8 **Figure S2. Identification of transformants as *T. scotoductu*s SA1 derivatives**. Illustrates that the pMKNor transformed cells were indeed SA-01 and not a contaminant. Click here for file
