# Supplementary material for: Sequence of the hyperplastic genome of the naturally competent Thermus scotoductus SA-01
Source: BMC Genomics. 2011 Nov 24;12:577. doi: 10.1186/1471-2164-12-577 (PMC3235269; doi:10.1186/1471-2164-12-577)
Supplement: Additional file 2 — Table S2. List of alien genes: Thermus scotoductus SA01. Contains a list of putative alien gens in Thermus scotoductus SA-01 as determined by codon bias relative to all genes using Karlin's codon bias method. [file 1471-2164-12-577-S2.DOC]

**LIST OF ALIEN GENES : *Thermus scotoductus* SA01**

**Determined by codon bias relative to all genes and selected other standards**

Standards: genomeCDS.cbRAll

genomeCDS.cbRRP

genomeCDS.cbRCH

genomeCDS.cbRTF

Number of genes: 2278

Criteria: all biases > threshold depending on gene length:

0.5223(100) 0.4550(150) 0.3856(250) 0.3395(400) 0.3031(600)

Eg(Standard) = Bias(All)/Bias(Standard)

Eg = Bias(All)/[0.5*Bias(RP)+0.25*Bias(CH)+0.25*Bias(TF)]

ALIEN GENES:

Eg B(all) EgRP B(RP) EgCH B(CH) EgTF B(TF) Ag Length S3 Position

**A** 0.83 0.415 0.84 0.495 0.80 0.519 0.83 0.500 0.078 264 68.18 9512

CDS complement(8715..9512)

/locus_tag="TSC_c00080"

/product="putative lipoprotein"

/protein_id="goetting:TSC_c00080"

**A** 0.87 0.548 0.91 0.602 0.83 0.657 0.84 0.653 0.140 153 62.75 60566

CDS complement(60105..60566)

/locus_tag="TSC_c00770"

/product="conserved hypothetical protein"

/protein_id="goetting:TSC_c00770"

**A** 0.85 0.524 0.85 0.614 0.85 0.617 0.83 0.628 0.049 100 68.00 60852

CDS complement(60547..60852)

/locus_tag="TSC_c00780"

/product="addiction module antitoxin, Axe family"

/protein_id="goetting:TSC_c00780"

**A** 0.88 0.543 0.90 0.607 0.85 0.637 0.87 0.624 0.048 91 76.92 70784

CDS complement(70506..70784)

/locus_tag="TSC_c00850"

/product="hypothetical protein"

/protein_id="goetting:TSC_c00850"

**A** 0.99 0.554 1.05 0.527 0.90 0.616 0.99 0.559 0.061 117 72.65 96984

CDS complement(96628..96984)

/locus_tag="TSC_c01120"

/product="conserved domain protein"

/protein_id="goetting:TSC_c01120"

HA 1.10 0.683 1.07 0.637 1.02 0.668 1.26 0.542 0.311 405 82.96 150776

CDS 150776..151996

/gene="tuf1"

/locus_tag="TSC_c01740"

/product="translation elongation factor Tu"

/protein_id="goetting:TSC_c01740"

**A** 0.82 0.418 0.82 0.507 0.90 0.462 0.73 0.573 0.090 281 74.38 249098

CDS complement(248250..249098)

/locus_tag="TSC_c02890"

/product="repeat motif-containing protein"

/protein_id="goetting:TSC_c02890"

**A** 0.83 0.596 0.85 0.704 0.80 0.743 0.82 0.728 0.291 301 61.46 253404

CDS 253404..254312

/locus_tag="TSC_c02930"

/product="rhamnosyl transferase"

/protein_id="goetting:TSC_c02930"

**A** 0.79 0.458 0.82 0.559 0.73 0.627 0.78 0.589 0.176 369 64.23 254454

CDS 254454..255566

/locus_tag="TSC_c02940"

/product="glycosyl transferase group 1"

/protein_id="goetting:TSC_c02940"

**A** 0.87 0.800 0.90 0.892 0.82 0.973 0.87 0.923 0.528 413 46.97 255830

CDS 255830..257074

/locus_tag="TSC_c02950"

/product="putative membrane protein"

/protein_id="goetting:TSC_c02950"

**A** 0.84 0.566 0.86 0.657 0.79 0.713 0.85 0.665 0.278 385 62.60 257071

CDS 257071..258231

/locus_tag="TSC_c02960"

/product="glycosyltransferase"

/protein_id="goetting:TSC_c02960"

**A** 0.79 0.439 0.82 0.538 0.75 0.584 0.79 0.558 0.152 373 65.68 258228

CDS 258228..259352

/gene="rfpB"

/locus_tag="TSC_c02970"

/product="putative galactosyltransferase"

/protein_id="goetting:TSC_c02970"

**A** 0.85 0.527 0.87 0.605 0.79 0.667 0.86 0.611 0.163 209 66.03 259975

CDS 259975..260607

/locus_tag="TSC_c02990"

/product="acetyltransferase with multiple hexapeptide

repeat domains"

/protein_id="goetting:TSC_c02990"

**A** 0.77 0.370 0.82 0.454 0.72 0.516 0.75 0.491 0.081 374 67.38 260604

CDS 260604..261731

/gene="spsC"

/locus_tag="TSC_c03000"

/product="spore coat polysaccharide biosynthesis protein

SpsC"

/protein_id="goetting:TSC_c03000"

**A** 0.93 0.689 0.91 0.754 0.94 0.734 0.95 0.726 0.170 84 67.86 266707

CDS 266707..266964

/locus_tag="TSC_c03050"

/product="transcriptional regulator, AbrB family"

/protein_id="goetting:TSC_c03050"

**A** 0.96 0.525 0.93 0.566 1.07 0.491 0.92 0.574 0.050 128 82.03 322708

CDS complement(322319..322708)

/locus_tag="TSC_c03750"

/product="domain of unknown function superfamily"

/protein_id="goetting:TSC_c03750"

**A** 0.75 0.374 0.77 0.487 0.76 0.489 0.72 0.521 0.090 376 67.29 351555

CDS complement(350422..351555)

/locus_tag="TSC_c04070"

/product="putative lipoprotein"

/protein_id="goetting:TSC_c04070"

**A** 0.90 0.537 0.93 0.578 0.84 0.642 0.91 0.588 0.075 120 70.83 360617

CDS 360617..360979

/locus_tag="TSC_c04180"

/product="conserved hypothetical protein"

/protein_id="goetting:TSC_c04180"

**A** 0.95 0.364 0.95 0.382 0.89 0.410 1.03 0.354 0.038 425 81.41 364429

CDS complement(363149..364429)

/locus_tag="TSC_c04220"

/product="transposase, IS4 family protein"

/protein_id="goetting:TSC_c04220"

**A** 0.84 0.632 0.87 0.727 0.82 0.774 0.83 0.764 0.330 323 56.04 384458

CDS complement(383484..384458)

/locus_tag="TSC_c04380"

/product="conserved hypothetical protein"

/protein_id="goetting:TSC_c04380"

**A** 0.80 0.471 0.83 0.567 0.80 0.589 0.76 0.618 0.083 159 67.30 384955

CDS complement(384473..384955)

/locus_tag="TSC_c04390"

/product="acetyltransferase, gnat family"

/protein_id="goetting:TSC_c04390"

**A** 0.85 0.521 0.87 0.599 0.81 0.646 0.87 0.602 0.151 205 64.39 388048

CDS complement(387428..388048)

/gene="queC"

/locus_tag="TSC_c04430"

/product="archaeosine biosynthesis protein QueC"

/protein_id="goetting:TSC_c04430"

**A** 0.90 0.516 0.92 0.564 0.94 0.550 0.85 0.607 0.047 118 74.58 394755

CDS complement(394396..394755)

/locus_tag="TSC_c04490"

/product="conserved hypothetical protein"

/protein_id="goetting:TSC_c04490"

**A** 0.95 0.364 0.95 0.382 0.89 0.410 1.03 0.354 0.038 425 81.41 412274

CDS complement(410994..412274)

/locus_tag="TSC_c04670"

/product="transposase, IS4 family protein"

/protein_id="goetting:TSC_c04670"

**A** 1.02 0.374 1.03 0.364 0.97 0.384 1.07 0.351 0.028 390 84.62 413710

CDS complement(412535..413710)

/locus_tag="TSC_c04700"

/product="DNA integration/recombination/invertion protein"

/protein_id="goetting:TSC_c04700"

**A** 0.85 0.668 0.87 0.770 0.80 0.834 0.86 0.779 0.415 527 54.08 417341

CDS 417341..418927

/locus_tag="TSC_c04750"

/product="putative ATP-dependent endonuclease of the OLD

family"

/protein_id="goetting:TSC_c04750"

**A** 0.80 0.448 0.83 0.537 0.75 0.599 0.78 0.572 0.205 1115 62.87 422423

CDS complement(419073..422423)

/locus_tag="TSC_c04760"

/product="ATPase"

/protein_id="goetting:TSC_c04760"

**A** 0.78 0.350 0.80 0.438 0.73 0.478 0.80 0.440 0.098 947 72.02 425335

CDS complement(422489..425335)

/locus_tag="TSC_c04770"

/product="protein of unknown function"

/protein_id="goetting:TSC_c04770"

**A** 0.76 0.343 0.80 0.428 0.70 0.486 0.75 0.460 0.097 1188 67.09 430685

CDS complement(427116..430685)

/locus_tag="TSC_c04780"

/product="type III restriction enzyme, subunit res"

/protein_id="goetting:TSC_c04780"

**A** 0.96 0.526 0.96 0.551 1.00 0.527 0.94 0.558 0.019 104 84.62 489451

CDS complement(489134..489451)

/locus_tag="TSC_c05370"

/product="putative nucleotidyltransferase protein"

/protein_id="goetting:TSC_c05370"

**A** 0.92 0.640 0.94 0.683 0.89 0.718 0.90 0.708 0.129 85 71.76 491320

CDS complement(491060..491320)

/locus_tag="TSC_c05400"

/product="conserved hypothetical protein"

/protein_id="goetting:TSC_c05400"

**A** 0.85 0.599 0.88 0.684 0.81 0.737 0.84 0.716 0.352 612 57.52 513678

CDS complement(511837..513678)

/locus_tag="TSC_c05610"

/product="conserved hypothetical protein"

/protein_id="goetting:TSC_c05610"

**A** 0.82 0.389 0.85 0.460 0.78 0.497 0.81 0.483 0.074 332 75.00 582309

CDS 582309..583310

/gene="dprA"

/locus_tag="TSC_c06380"

/product="competence protein DprA"

/protein_id="goetting:TSC_c06380"

**A** 0.84 0.504 0.87 0.577 0.80 0.629 0.80 0.627 0.085 137 65.69 621667

CDS complement(621251..621667)

/locus_tag="TSC_c06830"

/product="conserved hypothetical protein"

/protein_id="goetting:TSC_c06830"

**A** 0.96 0.467 0.95 0.491 1.09 0.427 0.87 0.538 0.060 206 83.01 654450

CDS complement(653827..654450)

/locus_tag="TSC_c07120"

/product="acetyltransferase"

/protein_id="goetting:TSC_c07120"

**A** 0.89 0.430 0.92 0.465 0.84 0.510 0.86 0.500 0.063 232 73.71 706394

CDS 706394..707095

/locus_tag="TSC_c07690"

/product="conserved hypothetical protein"

/protein_id="goetting:TSC_c07690"

**A** 0.81 0.546 0.82 0.666 0.80 0.680 0.79 0.694 0.301 553 57.50 709742

CDS complement(708078..709742)

/gene="comZ"

/locus_tag="TSC_c07710"

/product="ComZ"

/protein_id="goetting:TSC_c07710"

**A** 0.82 0.514 0.83 0.618 0.80 0.638 0.82 0.630 0.104 141 64.54 711438

CDS complement(711010..711438)

/locus_tag="TSC_c07720"

/product="conserved hypothetical protein"

/protein_id="goetting:TSC_c07720"

**A** 0.83 0.513 0.85 0.603 0.85 0.607 0.77 0.666 0.071 118 66.10 714090

CDS complement(713731..714090)

/locus_tag="TSC_c07760"

/product="nucleotidyltransferase"

/protein_id="goetting:TSC_c07760"

**A** 0.89 0.620 0.89 0.693 0.86 0.722 0.93 0.670 0.145 107 71.03 759668

CDS 759668..759994

/locus_tag="TSC_c08120"

/product="conserved hypothetical protein"

/protein_id="goetting:TSC_c08120"

**A** 0.92 0.353 0.98 0.362 0.84 0.420 0.92 0.384 0.068 964 78.01 764537

CDS complement(761640..764537)

/gene="hsdR1"

/locus_tag="TSC_c08160"

/product="type I restriction-modification system, subunit

R"

/protein_id="goetting:TSC_c08160"

**A** 0.84 0.606 0.86 0.708 0.84 0.722 0.81 0.751 0.313 352 58.24 765592

CDS complement(764534..765592)

/locus_tag="TSC_c08170"

/product="restriction modification system DNA specificity

domain"

/protein_id="goetting:TSC_c08170"

**A** 0.86 0.620 0.87 0.712 0.84 0.739 0.84 0.736 0.353 496 56.05 768566

CDS complement(767073..768566)

/locus_tag="TSC_c08200"

/product="hypothetical protein"

/protein_id="goetting:TSC_c08200"

**A** 0.82 0.505 0.84 0.599 0.79 0.642 0.82 0.618 0.259 1660 61.45 773548

CDS complement(768563..773548)

/locus_tag="TSC_c08210"

/product="conserved hypothetical protein"

/protein_id="goetting:TSC_c08210"

**A** 0.75 0.333 0.77 0.431 0.71 0.468 0.75 0.445 0.059 442 72.85 807232

CDS 807232..808563

/locus_tag="TSC_c08600"

/product="serine protease"

/protein_id="goetting:TSC_c08600"

**A** 0.88 0.558 0.89 0.626 0.84 0.664 0.91 0.613 0.062 91 74.73 869779

CDS complement(869501..869779)

/gene="rplG"

/locus_tag="TSC_c09160"

/product="50S ribosomal protein L7Ae family protein"

/protein_id="goetting:TSC_c09160"

**A** 0.85 0.570 0.87 0.654 0.80 0.717 0.87 0.659 0.204 202 63.37 956856

CDS 956856..957467

/locus_tag="TSC_c10060"

/product="conserved hypothetical protein"

/protein_id="goetting:TSC_c10060"

**A** 0.94 0.452 0.92 0.492 1.00 0.451 0.93 0.486 0.059 222 83.33 964768

CDS complement(964097..964768)

/locus_tag="TSC_c10180"

/product="hypothetical protein"

/protein_id="goetting:TSC_c10180"

**A** 0.80 0.418 0.82 0.511 0.75 0.554 0.80 0.520 0.089 255 67.06 983632

CDS 983632..984402

/locus_tag="TSC_c10390"

/product="hypothetical protein"

/protein_id="goetting:TSC_c10390"

**A** 0.84 0.611 0.86 0.708 0.81 0.758 0.83 0.738 0.143 94 62.77 1046337

CDS 1046337..1046624

/locus_tag="TSC_c10990"

/product="serine/threonine protein phosphatase"

/protein_id="goetting:TSC_c10990"

**A** 0.88 0.424 0.89 0.478 0.84 0.507 0.92 0.461 0.033 201 74.63 1048085

CDS 1048085..1048693

/locus_tag="TSC_c11020"

/product="hypothetical protein"

/protein_id="goetting:TSC_c11020"

**A** 0.86 0.457 0.89 0.512 0.81 0.567 0.84 0.541 0.066 182 68.13 1061049

CDS 1061049..1061600

/locus_tag="TSC_c11170"

/product="hypothetical protein"

/protein_id="goetting:TSC_c11170"

**A** 0.87 0.454 0.90 0.507 0.84 0.539 0.84 0.538 0.052 173 69.36 1069737

CDS 1069737..1070261

/locus_tag="TSC_c11250"

/product="rieske iron-sulfur protein"

/protein_id="goetting:TSC_c11250"

**A** 0.90 0.746 0.93 0.803 0.85 0.879 0.89 0.837 0.272 101 51.49 1071514

CDS 1071514..1071822

/locus_tag="TSC_c11280"

/product="hypothetical protein"

/protein_id="goetting:TSC_c11280"

**A** 0.83 0.511 0.87 0.586 0.78 0.657 0.82 0.619 0.206 333 59.76 1082784

CDS 1082784..1083788

/locus_tag="TSC_c11390"

/product="catechol 2,3 dioxygenase"

/protein_id="goetting:TSC_c11390"

**A** 0.81 0.530 0.84 0.632 0.77 0.685 0.79 0.674 0.189 218 61.47 1083788

CDS 1083788..1084447

/locus_tag="TSC_c11400"

/product="transcriptional regulator, GntR family"

/protein_id="goetting:TSC_c11400"

**A** 0.88 0.603 0.91 0.664 0.81 0.745 0.88 0.682 0.280 302 59.60 1084444

CDS 1084444..1085355

/locus_tag="TSC_c11410"

/product="conserved hypothetical protein"

/protein_id="goetting:TSC_c11410"

**A** 0.85 0.572 0.88 0.648 0.81 0.703 0.83 0.692 0.231 235 57.45 1085352

CDS 1085352..1086062

/locus_tag="TSC_c11420"

/product="LmbE family protein"

/protein_id="goetting:TSC_c11420"

**A** 0.90 0.669 0.94 0.710 0.84 0.800 0.91 0.735 0.303 221 55.20 1086072

CDS 1086072..1086740

/locus_tag="TSC_c11430"

/product="dimethylmenaquinone methyltransferase"

/protein_id="goetting:TSC_c11430"

**A** 0.86 0.650 0.90 0.718 0.79 0.821 0.83 0.779 0.329 263 56.65 1086733

CDS 1086733..1087527

/locus_tag="TSC_c11440"

/product="6-phosphogluconate dehydrogenase, NAD-binding

protein"

/protein_id="goetting:TSC_c11440"

**A** 0.87 0.556 0.89 0.624 0.84 0.663 0.85 0.651 0.262 403 60.05 1088788

CDS complement(1087574..1088788)

/locus_tag="TSC_c11450"

/note="phbh; 4-hydroxybenzoate 3-monooxygenase"

/product="P-hydroxybenzoate hydroxylase"

/protein_id="goetting:TSC_c11450"

**A** 0.90 0.558 0.91 0.612 0.87 0.639 0.92 0.604 0.042 82 65.85 1089032

CDS complement(1088781..1089032)

/locus_tag="TSC_c11460"

/product="dihydrodiol dehydrogenase"

/protein_id="goetting:TSC_c11460"

**A** 0.84 0.487 0.86 0.568 0.82 0.592 0.84 0.579 0.092 169 65.09 1089556

CDS complement(1089044..1089556)

/locus_tag="TSC_c11470"

/note="digoxigenin, subunit beta"

/product="3-phenylpropionate dioxygenase, subunit beta"

/protein_id="goetting:TSC_c11470"

**A** 0.76 0.356 0.80 0.445 0.73 0.487 0.72 0.496 0.084 444 68.92 1090903

CDS complement(1089566..1090903)

/locus_tag="TSC_c11480"

/note="biphenyl 2,3-dioxygenase"

/product="biphenyl dioxygenase, subunit alpha"

/protein_id="goetting:TSC_c11480"

**A** 0.77 0.367 0.81 0.452 0.74 0.499 0.73 0.505 0.102 483 66.46 1092368

CDS complement(1090914..1092368)

/locus_tag="TSC_c11490"

/product="ABC transporter ATP-binding/permease protein"

/protein_id="goetting:TSC_c11490"

**A** 0.78 0.383 0.79 0.485 0.78 0.492 0.76 0.502 0.073 316 68.04 1093308

CDS complement(1092355..1093308)

/locus_tag="TSC_c11500"

/product="membrane spanning protein"

/protein_id="goetting:TSC_c11500"

**A** 0.80 0.495 0.82 0.605 0.77 0.643 0.80 0.618 0.184 286 60.84 1094168

CDS complement(1093305..1094168)

/locus_tag="TSC_c11510"

/product="inner-membrane translocator"

/protein_id="goetting:TSC_c11510"

**A** 0.85 0.455 0.88 0.515 0.79 0.576 0.85 0.537 0.158 395 65.06 1095364

CDS complement(1094174..1095364)

/locus_tag="TSC_c11520"

/product="extracellular ligand-binding receptor"

/protein_id="goetting:TSC_c11520"

**A** 0.82 0.390 0.85 0.457 0.80 0.488 0.79 0.492 0.072 324 69.14 1096436

CDS complement(1095462..1096436)

/locus_tag="TSC_c11530"

/product="catechol 2,3 dioxygenase"

/protein_id="goetting:TSC_c11530"

**A** 0.85 0.504 0.86 0.584 0.85 0.590 0.83 0.610 0.073 134 66.42 1096840

CDS complement(1096433..1096840)

/locus_tag="TSC_c11540"

/product="carboxymuconolactone decarboxylase"

/protein_id="goetting:TSC_c11540"

**A** 0.83 0.408 0.86 0.474 0.76 0.540 0.84 0.485 0.054 230 67.39 1099930

CDS 1099930..1100625

/locus_tag="TSC_c11590"

/product="4-oxalocrotonate decarboxylase"

/protein_id="goetting:TSC_c11590"

**A** 0.78 0.325 0.81 0.403 0.71 0.455 0.79 0.411 0.053 501 72.26 1100636

CDS 1100636..1102144

/locus_tag="TSC_c11600"

/product="aldehyde dehydrogenase 5"

/protein_id="goetting:TSC_c11600"

**A** 0.86 0.421 0.88 0.481 0.79 0.537 0.89 0.471 0.075 257 74.71 1102149

CDS 1102149..1102925

/locus_tag="TSC_c11610"

/note="2-hydroxypentadienoic acidhydratase"

/product="2-keto-4-pentenoate hydratase"

/protein_id="goetting:TSC_c11610"

**A** 0.77 0.392 0.76 0.515 0.83 0.470 0.74 0.528 0.064 254 71.65 1118533

CDS complement(1117769..1118533)

/gene="hemD1"

/locus_tag="TSC_c11780"

/product="uroporphyrinogen-III synthase"

/protein_id="goetting:TSC_c11780"

**A** 0.76 0.348 0.76 0.459 0.77 0.455 0.75 0.467 0.092 548 71.72 1133890

CDS complement(1132241..1133890)

/locus_tag="TSC_c11950"

/product="diguanylate cyclase/phosphodiesterase with

PAS/PAC sensor"

/protein_id="goetting:TSC_c11950"

**A** 0.83 0.405 0.87 0.464 0.77 0.526 0.82 0.491 0.053 234 73.08 1136431

CDS 1136431..1137138

/locus_tag="TSC_c11980"

/product="transcriptional regulator, GntR family"

/protein_id="goetting:TSC_c11980"

**A** 0.77 0.372 0.80 0.462 0.72 0.516 0.77 0.486 0.125 592 66.55 1144513

CDS complement(1142732..1144513)

/locus_tag="TSC_c12030"

/product="amylo-alpha-1,6-glucosidase"

/protein_id="goetting:TSC_c12030"

**A** 0.76 0.342 0.78 0.440 0.74 0.463 0.77 0.447 0.058 409 72.86 1148248

CDS complement(1147016..1148248)

/locus_tag="TSC_c12060"

/product="extracellular solute-binding protein, family 1"

/protein_id="goetting:TSC_c12060"

**A** 0.80 0.421 0.84 0.502 0.75 0.559 0.77 0.549 0.116 324 65.74 1149292

CDS complement(1148315..1149292)

/locus_tag="TSC_c12070"

/product="transcriptional regulator, LacI family"

/protein_id="goetting:TSC_c12070"

**A** 0.95 0.579 0.98 0.590 0.89 0.649 0.94 0.616 0.057 86 69.77 1166416

CDS complement(1166153..1166416)

/locus_tag="TSC_c12280"

/product="transposase"

/protein_id="goetting:TSC_c12280"

**A** 0.92 0.536 0.94 0.569 0.87 0.613 0.92 0.585 0.048 106 70.75 1167066

CDS complement(1166743..1167066)

/locus_tag="TSC_c12290"

/product="transposase, ISLbp6"

/protein_id="goetting:TSC_c12290"

**A** 0.88 0.642 0.92 0.701 0.82 0.788 0.87 0.739 0.190 115 60.87 1180923

CDS complement(1180576..1180923)

/locus_tag="TSC_c12410"

/product="nucleotidyltransferase"

/protein_id="goetting:TSC_c12410"

**A** 0.86 0.516 0.87 0.593 0.85 0.610 0.86 0.598 0.233 476 67.23 1195885

CDS complement(1194452..1195885)

/locus_tag="TSC_c12530"

/product="conserved hypothetical protein"

/protein_id="goetting:TSC_c12530"

**A** 0.87 0.562 0.87 0.649 0.86 0.650 0.87 0.644 0.109 120 70.83 1196284

CDS complement(1195919..1196284)

/locus_tag="TSC_c12540"

/product="conserved hypothetical protein"

/protein_id="goetting:TSC_c12540"

**A** 0.88 0.517 0.86 0.598 0.96 0.539 0.86 0.603 0.042 112 71.43 1196622

CDS complement(1196281..1196622)

/locus_tag="TSC_c12550"

/product="conserved hypothetical protein"

/protein_id="goetting:TSC_c12550"

**A** 0.81 0.420 0.83 0.507 0.76 0.550 0.82 0.509 0.091 270 75.19 1199767

CDS 1199767..1200582

/locus_tag="TSC_c12620"

/product="family transposase"

/protein_id="goetting:TSC_c12620"

**A** 0.92 0.536 0.94 0.569 0.87 0.613 0.92 0.585 0.048 106 70.75 1205761

CDS complement(1205438..1205761)

/locus_tag="TSC_c12690"

/product="transposase, ISLbp6"

/protein_id="goetting:TSC_c12690"

**A** 0.91 0.342 0.94 0.363 0.85 0.404 0.92 0.371 0.035 476 75.00 1226502

CDS complement(1225069..1226502)

/locus_tag="TSC_c12850"

/note="badh"

/product="betaine aldehyde dehydrogenase"

/protein_id="goetting:TSC_c12850"

**A** 0.87 0.467 0.90 0.517 0.78 0.600 0.90 0.519 0.124 261 70.11 1227301

CDS complement(1226513..1227301)

/locus_tag="TSC_c12860"

/product="ABC transporter permease protein"

/protein_id="goetting:TSC_c12860"

**A** 0.83 0.386 0.87 0.441 0.72 0.539 0.86 0.449 0.061 301 71.10 1228218

CDS complement(1227310..1228218)

/locus_tag="TSC_c12870"

/product="ABC transporter permease protein"

/protein_id="goetting:TSC_c12870"

**A** 0.82 0.446 0.85 0.522 0.75 0.594 0.82 0.541 0.139 333 65.17 1229222

CDS complement(1228218..1229222)

/gene="potA3"

/locus_tag="TSC_c12880"

/product="spermidine/putrescine import ATP-binding protein

PotA"

/protein_id="goetting:TSC_c12880"

**A** 0.75 0.351 0.77 0.454 0.70 0.499 0.77 0.456 0.067 387 73.64 1230392

CDS complement(1229226..1230392)

/locus_tag="TSC_c12890"

/product="ABC transporter, periplasmic substrate-binding

protein"

/protein_id="goetting:TSC_c12890"

**A** 0.79 0.442 0.83 0.531 0.74 0.597 0.75 0.588 0.074 182 63.19 1269521

CDS 1269521..1270072

/locus_tag="TSC_c13220"

/product="putative lipoprotein"

/protein_id="goetting:TSC_c13220"

**A** 0.82 0.451 0.86 0.525 0.80 0.563 0.76 0.595 0.080 192 63.54 1273964

CDS 1273964..1274545

/gene="pcp"

/locus_tag="TSC_c13250"

/note="pyrase; PGP-I; pyroglutamyl-peptidase I;

5-oxoprolyl-peptidase"

/product="pyrrolidone-carboxylate peptidase"

/protein_id="goetting:TSC_c13250"

**A** 0.76 0.376 0.77 0.485 0.77 0.488 0.73 0.516 0.079 344 68.02 1274542

CDS 1274542..1275579

/gene="rfaG"

/locus_tag="TSC_c13260"

/product="lipopolysaccharide core biosynthesis protein

RfaG"

/protein_id="goetting:TSC_c13260"

**A** 0.82 0.534 0.86 0.619 0.77 0.697 0.80 0.668 0.245 357 61.06 1275573

CDS 1275573..1276649

/locus_tag="TSC_c13270"

/product="glycosyltransferase"

/protein_id="goetting:TSC_c13270"

**A** 0.96 0.552 0.97 0.571 0.88 0.627 1.03 0.536 0.056 110 80.00 1324022

CDS complement(1323687..1324022)

/locus_tag="TSC_c13740"

/product="conserved hypothetical protein"

/protein_id="goetting:TSC_c13740"

**A** 0.96 0.618 0.98 0.634 0.91 0.678 0.98 0.633 0.087 82 76.83 1324730

CDS 1324730..1324978

/locus_tag="TSC_c13780"

/product="hypothetical protein"

/protein_id="goetting:TSC_c13780"

**A** 0.82 0.456 0.84 0.543 0.77 0.589 0.84 0.542 0.191 529 68.05 1326554

CDS complement(1324962..1326554)

/locus_tag="TSC_c13790"

/product="PQQ enzyme repeat domain protein"

/protein_id="goetting:TSC_c13790"

**A** 0.92 0.498 0.94 0.529 0.84 0.594 0.97 0.516 0.090 182 71.98 1327181

CDS complement(1326630..1327181)

/locus_tag="TSC_c13800"

/product="putative MFS transporter, ADT family"

/protein_id="goetting:TSC_c13800"

**A** 0.76 0.338 0.80 0.425 0.69 0.488 0.76 0.443 0.085 561 70.41 1403096

CDS complement(1401408..1403096)

/locus_tag="TSC_c14590"

/product="two-component sensor histidine kinase"

/protein_id="goetting:TSC_c14590"

**A** 0.92 0.638 0.93 0.683 0.88 0.729 0.94 0.677 0.133 91 74.73 1470994

CDS complement(1470716..1470994)

/locus_tag="TSC_c15290"

/product="conserved hypothetical protein"

/protein_id="goetting:TSC_c15290"

**A** 0.83 0.352 0.88 0.402 0.75 0.469 0.82 0.427 0.047 380 73.68 1473258

CDS complement(1472113..1473258)

/gene="argJ"

/locus_tag="TSC_c15320"

/product="glutamate N-acetyltransferase/amino-acid

acetyltransferase"

/protein_id="goetting:TSC_c15320"

**A** 0.83 0.507 0.87 0.581 0.77 0.656 0.82 0.618 0.123 172 64.53 1474810

CDS complement(1474289..1474810)

/locus_tag="TSC_c15340"

/product="conserved hypothetical protein"

/protein_id="goetting:TSC_c15340"

**A** 0.93 0.342 0.97 0.354 0.86 0.399 0.93 0.369 0.041 522 80.08 1505296

CDS 1505296..1506867

/locus_tag="TSC_c15640"

/product="type I restriction-modification system, subunit

M"

/protein_id="goetting:TSC_c15640"

**A** 0.83 0.576 0.86 0.669 0.78 0.737 0.82 0.700 0.308 449 57.24 1506864

CDS 1506864..1508216

/locus_tag="TSC_c15650"

/product="restriction modification system DNA specificity

domain"

/protein_id="goetting:TSC_c15650"

**A** 0.93 0.486 0.96 0.505 0.89 0.549 0.92 0.529 0.202 588 68.54 1508232

CDS 1508232..1510001

/locus_tag="TSC_c15660"

/product="protein of unknown function"

/protein_id="goetting:TSC_c15660"

**A** 0.91 0.324 0.97 0.335 0.83 0.391 0.91 0.358 0.040 971 80.02 1510018

CDS 1510018..1512936

/gene="hsdR2"

/locus_tag="TSC_c15670"

/product="type I restriction-modification system, subunit

R"

/protein_id="goetting:TSC_c15670"

**A** 0.88 0.487 0.87 0.558 0.87 0.559 0.89 0.545 0.039 130 72.31 1524707

CDS 1524707..1525099

/locus_tag="TSC_c15790"

/product="death-on-curing family protein"

/protein_id="goetting:TSC_c15790"

**A** 0.91 0.436 0.88 0.495 0.96 0.455 0.91 0.479 0.022 180 81.67 1525667

CDS complement(1525122..1525667)

/locus_tag="TSC_c15800"

/product="hypothetical protein"

/protein_id="goetting:TSC_c15800"

**A** 0.94 0.622 0.93 0.669 1.00 0.621 0.91 0.686 0.180 146 74.66 1530445

CDS complement(1530005..1530445)

/locus_tag="TSC_c15850"

/product="conserved hypothetical protein"

/protein_id="goetting:TSC_c15850"

**A** 0.87 0.457 0.86 0.529 0.86 0.530 0.88 0.521 0.042 157 75.80 1530896

CDS complement(1530420..1530896)

/locus_tag="TSC_c15860"

/product="hypothetical protein"

/protein_id="goetting:TSC_c15860"

**A** 0.90 0.403 0.88 0.456 0.92 0.438 0.93 0.433 0.067 349 78.51 1533021

CDS complement(1531969..1533021)

/locus_tag="TSC_c15880"

/product="integral membrane protein/HD-hydrolase domain"

/protein_id="goetting:TSC_c15880"

**A** 0.87 0.471 0.90 0.525 0.80 0.590 0.88 0.534 0.088 197 75.13 1533741

CDS complement(1533145..1533741)

/locus_tag="TSC_c15890"

/product="transposase"

/protein_id="goetting:TSC_c15890"

**A** 0.88 0.437 0.88 0.495 0.96 0.456 0.82 0.535 0.070 235 77.45 1535384

CDS complement(1534674..1535384)

/locus_tag="TSC_c15910"

/product="conserved hypothetical protein"

/protein_id="goetting:TSC_c15910"

**A** 0.85 0.540 0.86 0.629 0.82 0.659 0.87 0.619 0.134 151 70.86 1535401

CDS 1535401..1535859

/locus_tag="TSC_c15920"

/product="hypothetical protein"

/protein_id="goetting:TSC_c15920"

**A** 0.83 0.445 0.83 0.537 0.89 0.500 0.77 0.576 0.133 339 73.16 1539355

CDS complement(1538333..1539355)

/locus_tag="TSC_c15990"

/product="putative DNA polymerase III, subunit beta"

/protein_id="goetting:TSC_c15990"

**A** 0.99 0.870 0.96 0.903 1.07 0.810 0.98 0.892 0.320 80 76.25 1542904

CDS complement(1542659..1542904)

/locus_tag="TSC_c16080"

/product="hypothetical protein"

/protein_id="goetting:TSC_c16080"

**A** 0.93 0.651 0.91 0.716 1.03 0.630 0.89 0.736 0.199 136 77.94 1543373

CDS complement(1542960..1543373)

/locus_tag="TSC_c16090"

/product="hypothetical protein"

/protein_id="goetting:TSC_c16090"

**A** 1.00 0.698 0.98 0.709 1.10 0.637 0.95 0.736 0.175 101 78.22 1543854

CDS complement(1543546..1543854)

/locus_tag="TSC_c16110"

/product="conserved hypothetical protein"

/protein_id="goetting:TSC_c16110"

**A** 0.94 0.593 0.94 0.630 0.99 0.598 0.90 0.660 0.160 155 75.48 1544367

CDS complement(1543897..1544367)

/locus_tag="TSC_c16120"

/product="Rad52/22 double-strand break repair protein"

/protein_id="goetting:TSC_c16120"

**A** 0.99 0.643 0.96 0.670 1.04 0.617 0.99 0.648 0.130 106 76.42 1544745

CDS complement(1544422..1544745)

/locus_tag="TSC_c16130"

/product="hypothetical protein"

/protein_id="goetting:TSC_c16130"

**A** 0.91 0.786 0.91 0.867 0.94 0.837 0.90 0.871 0.295 97 62.89 1545242

CDS complement(1544946..1545242)

/locus_tag="TSC_c16140"

/product="hypothetical protein"

/protein_id="goetting:TSC_c16140"

**A** 0.99 0.604 0.98 0.619 1.10 0.547 0.93 0.652 0.302 620 82.42 1547515

CDS complement(1545650..1547515)

/gene="pcrA2"

/locus_tag="TSC_c16170"

/product="ATP-dependent DNA helicase PcrA"

/protein_id="goetting:TSC_c16170"

**A** 0.95 0.557 0.94 0.594 1.07 0.523 0.89 0.628 0.224 379 81.53 1549031

CDS complement(1547889..1549031)

/locus_tag="TSC_c16190"

/product="conserved hypothetical protein"

/protein_id="goetting:TSC_c16190"

**A** 0.89 0.482 0.89 0.540 0.96 0.502 0.84 0.574 0.162 373 77.75 1550165

CDS complement(1549041..1550165)

/locus_tag="TSC_c16200"

/product="ATPase associated with various cellular

activities, AAA_3"

/protein_id="goetting:TSC_c16200"

**A** 0.94 0.645 0.91 0.709 1.04 0.622 0.92 0.702 0.120 86 76.74 1551735

CDS complement(1551472..1551735)

/locus_tag="TSC_c16240"

/product="hypothetical protein"

/protein_id="goetting:TSC_c16240"

**A** 0.83 0.437 0.85 0.513 0.76 0.575 0.86 0.508 0.085 230 73.04 1553642

CDS complement(1552947..1553642)

/locus_tag="TSC_c16260"

/product="transcriptional regulator, ArsR family protein"

/protein_id="goetting:TSC_c16260"

**A** 0.95 0.703 0.96 0.734 0.88 0.796 1.01 0.697 0.249 136 70.59 1554703

CDS complement(1554290..1554703)

/locus_tag="TSC_c16290"

/product="hypothetical protein"

/protein_id="goetting:TSC_c16290"

**A** 0.82 0.411 0.82 0.502 0.77 0.535 0.86 0.478 0.113 381 76.38 1558300

CDS complement(1557152..1558300)

/locus_tag="TSC_c16310"

/product="hypothetical protein"

/protein_id="goetting:TSC_c16310"

**A** 0.86 0.380 0.86 0.443 0.81 0.471 0.91 0.417 0.109 819 74.48 1561849

CDS complement(1559387..1561849)

/locus_tag="TSC_c16330"

/note="-40"

/product="hypothetical NTPase"

/protein_id="goetting:TSC_c16330"

**A** 0.90 0.740 0.92 0.808 0.85 0.868 0.91 0.813 0.268 104 60.58 1562126

CDS complement(1561809..1562126)

/locus_tag="TSC_c16340"

/product="hypothetical protein"

/protein_id="goetting:TSC_c16340"

**A** 0.95 0.587 0.95 0.618 0.88 0.670 1.02 0.573 0.100 114 68.42 1562588

CDS complement(1562241..1562588)

/locus_tag="TSC_c16350"

/product="hypothetical protein"

/protein_id="goetting:TSC_c16350"

**A** 0.83 0.572 0.85 0.676 0.81 0.708 0.83 0.687 0.279 360 62.78 1563450

CDS 1563450..1564535

/locus_tag="TSC_c16380"

/product="hypothetical protein"

/protein_id="goetting:TSC_c16380"

**A** 0.83 0.500 0.84 0.595 0.81 0.617 0.84 0.597 0.170 263 65.40 1564529

CDS 1564529..1565323

/locus_tag="TSC_c16390"

/product="conserved hypothetical protein"

/protein_id="goetting:TSC_c16390"

**A** 0.88 0.635 0.89 0.711 0.83 0.767 0.90 0.704 0.255 192 66.67 1566210

CDS complement(1565629..1566210)

/locus_tag="TSC_c16400"

/product="hypothetical protein"

/protein_id="goetting:TSC_c16400"

**A** 0.82 0.506 0.84 0.603 0.76 0.663 0.85 0.595 0.142 199 68.34 1566788

CDS complement(1566186..1566788)

/gene="trbP"

/locus_tag="TSC_c16410"

/product="TrbP protein"

/protein_id="goetting:TSC_c16410"

**A** 0.89 0.603 0.88 0.688 0.85 0.709 0.94 0.641 0.158 129 68.99 1567177

CDS complement(1566785..1567177)

/locus_tag="TSC_c16420"

/product="hypothetical protein"

/protein_id="goetting:TSC_c16420"

**A** 0.97 0.319 0.93 0.342 0.98 0.324 1.04 0.306 0.018 609 89.49 1571869

CDS complement(1570037..1571869)

/locus_tag="TSC_c16460"

/product="hypothetical protein"

/protein_id="goetting:TSC_c16460"

**A** 0.82 0.449 0.82 0.547 0.78 0.579 0.87 0.518 0.184 539 74.95 1574905

CDS complement(1573283..1574905)

/locus_tag="TSC_c16510"

/product="putative lipoprotein"

/protein_id="goetting:TSC_c16510"

**A** 0.85 0.476 0.84 0.563 0.80 0.598 0.90 0.527 0.174 381 72.44 1576047

CDS complement(1574899..1576047)

/locus_tag="TSC_c16520"

/product="S-layer protein-related protein"

/protein_id="goetting:TSC_c16520"

**A** 0.85 0.490 0.85 0.576 0.81 0.605 0.90 0.542 0.147 252 75.00 1576808

CDS complement(1576047..1576808)

/locus_tag="TSC_c16530"

/product="hypothetical protein"

/protein_id="goetting:TSC_c16530"

**A** 0.92 0.591 0.92 0.641 0.90 0.660 0.92 0.640 0.074 83 74.70 1577066

CDS complement(1576812..1577066)

/locus_tag="TSC_c16540"

/product="hypothetical protein"

/protein_id="goetting:TSC_c16540"

**A** 0.87 0.495 0.87 0.570 0.86 0.575 0.90 0.550 0.135 236 73.31 1579687

CDS complement(1578974..1579687)

/locus_tag="TSC_c16570"

/product="putative peptidase"

/protein_id="goetting:TSC_c16570"

**A** 0.90 0.524 0.90 0.579 0.85 0.619 0.93 0.562 0.108 161 76.40 1580971

CDS complement(1580483..1580971)

/locus_tag="TSC_c16590"

/product="hypothetical protein"

/protein_id="goetting:TSC_c16590"

**A** 0.89 0.465 0.88 0.526 0.86 0.538 0.92 0.504 0.055 173 73.99 1581833

CDS complement(1581309..1581833)

/locus_tag="TSC_c16610"

/product="hypothetical protein"

/protein_id="goetting:TSC_c16610"

**A** 0.84 0.496 0.83 0.595 0.81 0.614 0.88 0.562 0.097 163 75.46 1582305

CDS complement(1581811..1582305)

/locus_tag="TSC_c16620"

/product="hypothetical protein"

/protein_id="goetting:TSC_c16620"

**A** 0.95 0.635 0.95 0.670 1.01 0.627 0.90 0.705 0.148 114 72.81 1587205

CDS 1587205..1587552

/locus_tag="TSC_c16680"

/product="LexA repressor"

/protein_id="goetting:TSC_c16680"

**A** 0.95 0.359 0.95 0.377 0.89 0.405 1.03 0.349 0.033 425 81.41 1587881

CDS 1587881..1589161

/locus_tag="TSC_c16690"

/product="transposase, IS4 family protein"

/protein_id="goetting:TSC_c16690"

**A** 0.80 0.342 0.81 0.423 0.78 0.439 0.80 0.426 0.063 492 74.80 1591600

CDS 1591600..1593081

/locus_tag="TSC_c16730"

/note="aldh-e2; aldhi; ahd-m1; aldhclass 2"

/product="aldehyde dehydrogenase"

/protein_id="goetting:TSC_c16730"

**A** 0.86 0.368 0.92 0.402 0.79 0.463 0.83 0.443 0.037 317 71.29 1593307

CDS 1593307..1594263

/locus_tag="TSC_c16740"

/note="-46"

/product="ABC transporter"

/protein_id="goetting:TSC_c16740"

**A** 1.03 0.539 1.01 0.534 1.16 0.463 0.96 0.564 0.180 366 87.43 1607202

CDS 1607202..1608305

/locus_tag="TSC_c16930"

/product="phage integrase"

/protein_id="goetting:TSC_c16930"

**A** 0.97 0.634 0.97 0.653 0.90 0.702 1.06 0.600 0.204 173 79.77 1639360

CDS complement(1638839..1639360)

/locus_tag="TSC_c17270"

/product="transposase family protein"

/protein_id="goetting:TSC_c17270"

**A** 0.91 0.382 0.86 0.443 1.08 0.352 0.85 0.449 0.048 365 84.38 1677301

CDS 1677301..1678401

/locus_tag="TSC_c17700"

/product="hypothetical membrane spanning protein"

/protein_id="goetting:TSC_c17700"

**A** 0.92 0.523 0.94 0.558 0.84 0.622 0.97 0.538 0.059 125 72.80 1722735

CDS complement(1722355..1722735)

/locus_tag="TSC_c18160"

/note="-34"

/product="PilT protein domain protein"

/protein_id="goetting:TSC_c18160"

**A** 0.97 0.371 0.97 0.383 0.90 0.413 1.05 0.352 0.041 421 81.71 1753122

CDS complement(1751857..1753122)

/locus_tag="TSC_c18490"

/note="-34"

/product="transposase, IS4 family protein"

/protein_id="goetting:TSC_c18490"

**A** 0.76 0.412 0.79 0.523 0.72 0.572 0.74 0.559 0.133 368 67.93 1761751

CDS complement(1760642..1761751)

/locus_tag="TSC_c18570"

/product="hypothetical protein"

/protein_id="goetting:TSC_c18570"

**A** 0.80 0.393 0.83 0.471 0.76 0.514 0.79 0.496 0.055 246 71.95 1791413

CDS complement(1790670..1791413)

/locus_tag="TSC_c18850"

/product="conserved hypothetical protein"

/protein_id="goetting:TSC_c18850"

**A** 0.85 0.610 0.88 0.695 0.81 0.753 0.84 0.723 0.162 113 64.60 1792601

CDS complement(1792257..1792601)

/locus_tag="TSC_c18870"

/product="hypothetical protein"

/protein_id="goetting:TSC_c18870"

**A** 0.78 0.383 0.82 0.466 0.73 0.525 0.76 0.505 0.080 330 66.36 1794029

CDS 1794029..1795024

/locus_tag="TSC_c18900"

/product="restriction endonuclease"

/protein_id="goetting:TSC_c18900"

**A** 0.94 0.450 0.94 0.479 0.96 0.470 0.94 0.478 0.031 183 74.86 1798898

CDS 1798898..1799452

/locus_tag="TSC_c18950"

/product="protease I"

/protein_id="goetting:TSC_c18950"

**A** 0.82 0.420 0.83 0.506 0.81 0.522 0.82 0.513 0.140 470 69.15 1955796

CDS 1955796..1957211

/locus_tag="TSC_c20630"

/product="integral membrane protein/HD-hydrolase domain"

/protein_id="goetting:TSC_c20630"

**A** 0.81 0.475 0.84 0.567 0.78 0.610 0.78 0.611 0.169 313 64.22 1961372

CDS 1961372..1962316

/gene="pilW"

/locus_tag="TSC_c20690"

/product="competence protein PilW"

/protein_id="goetting:TSC_c20690"

**A** 0.88 0.550 0.90 0.614 0.87 0.630 0.86 0.637 0.097 123 65.04 1967404

CDS 1967404..1967775

/locus_tag="TSC_c20740"

/product="PIN domain protein"

/protein_id="goetting:TSC_c20740"

**A** 0.88 0.613 0.91 0.674 0.81 0.759 0.91 0.672 0.354 623 62.76 2086010

CDS complement(2084136..2086010)

/locus_tag="TSC_c22080"

/product="fibronectin, type III domain protein"

/protein_id="goetting:TSC_c22080"

**A** 0.90 0.557 0.93 0.596 0.83 0.667 0.90 0.616 0.150 170 65.88 2086543

CDS complement(2086028..2086543)

/locus_tag="TSC_c22090"

/product="putative lipoprotein"

/protein_id="goetting:TSC_c22090"

HA 1.09 0.682 1.07 0.640 1.02 0.672 1.25 0.547 0.312 405 82.22 2107377

CDS complement(2106157..2107377)

/gene="tuf2"

/locus_tag="TSC_c22460"

/product="translation elongation factor Tu"

/protein_id="goetting:TSC_c22460"

**A** 0.82 0.452 0.84 0.537 0.79 0.573 0.79 0.571 0.078 189 69.31 2187791

CDS 2187791..2188360

/locus_tag="TSC_c23280"

/product="conserved hypothetical protein"

/protein_id="goetting:TSC_c23280"

**A** 0.85 0.619 0.84 0.738 0.88 0.707 0.83 0.746 0.138 89 64.04 2201545

CDS 2201545..2201817

/locus_tag="TSC_c23410"

/product="conserved hypothetical protein"

/protein_id="goetting:TSC_c23410"

**A** 0.85 0.534 0.85 0.627 0.87 0.613 0.83 0.646 0.071 109 66.06 2235344

CDS 2235344..2235676

/locus_tag="TSC_c23800"

/product="putative nucleotidyltransferase domain"

/protein_id="goetting:TSC_c23800"

**A** 0.85 0.478 0.86 0.559 0.86 0.554 0.83 0.577 0.060 146 70.55 2291961

CDS 2291961..2292404

/locus_tag="TSC_c24430"

/product="putative PIN domain"

/protein_id="goetting:TSC_c24430"
