# Supplementary material for: Sequence of the hyperplastic genome of the naturally competent Thermus scotoductus SA-01
Source: BMC Genomics. 2011 Nov 24;12:577. doi: 10.1186/1471-2164-12-577 (PMC3235269; doi:10.1186/1471-2164-12-577)
Supplement: Additional file 4 — Table S4. List of alien genes: Thermus thermophilus HB8 chromosome and megaplasmid. Contains a list of putative alien gens in Thermus thermophilusHB8 as determined by codon bias relative to all genes using Karlin's codon bias method. [file 1471-2164-12-577-S4.DOC]

**LIST OF ALIEN GENES: *Thermus thermophilus* HB8 chromosome and megaplasmid.**

**Determined by codon bias relative to all genes and selected other standards**

Standards: chromosome1CDS.cbRAll

chromosome1CDS.cbRRP

chromosome1CDS.cbRCH

chromosome1CDS.cbRTF

Number of genes: 1868

Criteria: all biases > threshold depending on gene length:

0.4275(100) 0.3830(150) 0.3268(250) 0.2886(400) 0.2533(600)

Eg(Standard) = Bias(All)/Bias(Standard)

Eg = Bias(All)/[0.5*Bias(RP)+0.25*Bias(CH)+0.25*Bias(TF)]

ALIEN GENES:

Eg B(all) EgRP B(RP) EgCH B(CH) EgTF B(TF) Ag Length S3 Position

**A** 0.90 0.690 0.93 0.739 0.89 0.774 0.85 0.810 0.424 323 61.30 32270

CDS 32270..33244

/gene="TTHA0030"

/inference="non-experimental evidence, no additional

details recorded"

/note="similar to GB:AAS80924.1 percent identity 72 in 29

aa"

/product="hypothetical protein"

/protein_id="BAD69853.1"

**A** 0.81 0.367 0.87 0.421 0.79 0.464 0.73 0.501 0.122 384 77.60 33231

CDS 33231..34388

/gene="TTHA0031"

/inference="non-experimental evidence, no additional

details recorded"

/note="similar to GB:AAS82309.1 percent identity 48 in 181

aa"

/product="conserved hypothetical membrane protein"

/protein_id="BAD69854.1"

**A** 0.95 0.433 0.97 0.446 0.91 0.477 0.96 0.451 0.051 137 86.13 34687

CDS 34687..35103

/gene="TTHA0033"

/inference="non-experimental evidence, no additional

details recorded"

/note="similar to REF:ZP_00340578.1 percent identity 31 in

120 aa"

/product="hypothetical protein"

/protein_id="BAD69856.1"

**A** 0.93 0.816 0.95 0.858 0.93 0.881 0.89 0.920 0.542 320 56.56 35234

CDS 35234..36199

/gene="TTHA0034"

/inference="non-experimental evidence, no additional

details recorded"

/note="similar to GB:AAS80924.1 percent identity 68 in 29

aa"

/product="hypothetical protein"

/protein_id="BAD69857.1"

**A** 0.85 0.478 0.89 0.538 0.84 0.568 0.81 0.594 0.225 367 70.30 36233

CDS 36233..37336

/gene="TTHA0035"

/inference="non-experimental evidence, no additional

details recorded"

/note="similar to REF:ZP_00226519.1 percent identity 28 in

376 aa"

/product="hypothetical membrane protein"

/protein_id="BAD69858.1"

**A** 0.92 0.751 0.95 0.794 0.92 0.814 0.88 0.857 0.475 310 58.71 37564

CDS 37564..38499

/gene="TTHA0036"

/inference="non-experimental evidence, no additional

details recorded"

/note="similar to GB:AAS80924.1 percent identity 75 in 29

aa"

/product="hypothetical protein"

/protein_id="BAD69859.1"

**A** 0.88 0.448 0.90 0.500 0.89 0.504 0.85 0.530 0.102 159 76.10 145229

CDS complement(144747..145229)

/gene="TTHA0150"

/inference="non-experimental evidence, no additional

details recorded"

/note="similar to PDB:1BKG percent identity 32 in 121 aa"

/product="hypothetical protein"

/protein_id="BAD69973.1"

**A** 0.85 0.404 0.85 0.475 0.83 0.484 0.88 0.460 0.053 147 80.95 178038

CDS 178038..178484

/gene="TTHA0182"

/inference="non-experimental evidence, no additional

details recorded"

/note="similar to GB:AAS82146.1 percent identity 98 in 148

aa"

/product="conserved hypothetical protein"

/protein_id="BAD70005.1"

**A** 0.89 0.352 0.92 0.384 0.87 0.403 0.85 0.412 0.060 293 87.03 187400

CDS complement(186516..187400)

/gene="TTHA0191"

/inference="non-experimental evidence, no additional

details recorded"

/note="similar to GB:AAS82137.1 percent identity 98 in 294

aa"

/product="dihydropteroate synthase"

/protein_id="BAD70014.1"

**A** 0.85 0.442 0.91 0.487 0.83 0.530 0.77 0.574 0.198 405 81.73 228697

CDS complement(227477..228697)

/gene="TTHA0234"

/inference="non-experimental evidence, no additional

details recorded"

/note="similar to DBJ:BAC55317.1 percent identity 99 in

405 aa"

/product="transposase"

/protein_id="BAD70057.1"

**A** 0.82 0.454 0.86 0.526 0.81 0.561 0.74 0.611 0.229 442 75.79 230754

CDS complement(229423..230754)

/gene="TTHA0236"

/inference="non-experimental evidence, no additional

details recorded"

/note="similar to GB:AAS82092.1 percent identity 99 in 443

aa"

/product="conserved hypothetical protein"

/protein_id="BAD70059.1"

**A** 0.79 0.413 0.77 0.536 0.78 0.529 0.84 0.495 0.055 118 88.98 232060

CDS complement(231701..232060)

/gene="TTHA0238"

/inference="non-experimental evidence, no additional

details recorded"

/note="similar to GB:AAS82090.1 percent identity 99 in 119

aa"

/product="hypothetical protein"

/protein_id="BAD70061.1"

**A** 0.99 0.717 1.02 0.705 0.98 0.730 0.95 0.752 0.435 405 84.69 240167

CDS complement(238947..240167)

/gene="TTHA0251"

/inference="non-experimental evidence, no additional

details recorded"

/note="similar to EMB:CAA43956.1 percent identity 100 in

406 aa"

/product="translation elongation factor EF-Tu.B"

/protein_id="BAD70074.1"

**A** 0.87 0.552 0.90 0.616 0.88 0.629 0.81 0.679 0.258 229 85.59 261143

CDS complement(260454..261143)

/gene="TTHA0273"

/inference="non-experimental evidence, no additional

details recorded"

/note="similar to GB:AAS81959.1 percent identity 99 in 229

aa"

/product="hypothetical protein (transposase related

protein)"

/protein_id="BAD70096.1"

**A** 0.95 0.267 0.92 0.290 0.99 0.271 0.96 0.278 0.020 663 88.24 273598

CDS 273598..275592

/gene="TTHA0285"

/inference="non-experimental evidence, no additional

details recorded"

/note="similar to GB:AAF12643.1 percent identity 24 in 691

aa"

/product="conserved hypothetical protein"

/protein_id="BAD70108.1"

**A** 0.87 0.552 0.90 0.616 0.88 0.629 0.81 0.679 0.258 229 85.59 338921

CDS 338921..339610

/gene="TTHA0358"

/inference="non-experimental evidence, no additional

details recorded"

/note="similar to GB:AAS81959.1 percent identity 99 in 229

aa"

/product="hypothetical protein"

/protein_id="BAD70181.1"

**A** 0.89 0.478 0.89 0.539 0.89 0.535 0.88 0.546 0.092 111 83.78 372275

CDS 372275..372613

/gene="TTHA0393"

/inference="non-experimental evidence, no additional

details recorded"

/note="similar to GB:AAS80373.1 percent identity 96 in 112

aa"

/product="conserved hypothetical protein"

/protein_id="BAD70216.1"

**A** 0.85 0.564 0.89 0.635 0.85 0.665 0.79 0.714 0.270 212 69.34 389286

CDS 389286..389927

/gene="TTHA0410"

/inference="non-experimental evidence, no additional

details recorded"

/note="similar to GB:AAS80390.1 percent identity 96 in 105

aa"

/product="hypothetical protein"

/protein_id="BAD70233.1"

**A** 0.91 0.674 0.92 0.730 0.91 0.738 0.86 0.781 0.322 142 84.51 436717

CDS complement(436289..436717)

/gene="TTHA0467"

/inference="non-experimental evidence, no additional

details recorded"

/note="similar to GB:AAS81959.1 percent identity 100 in

142 aa"

/product="hypothetical protein"

/protein_id="BAD70290.1"

**A** 0.99 0.558 1.07 0.521 0.97 0.572 0.87 0.640 0.199 175 75.43 437261

CDS complement(436731..437261)

/gene="TTHA0468"

/inference="non-experimental evidence, no additional

details recorded"

/note="similar to GB:AAS80749.1 percent identity 90 in 134

aa"

/product="hypothetical protein"

/protein_id="BAD70291.1"

**A** 0.91 0.443 0.95 0.464 0.90 0.490 0.85 0.519 0.163 339 90.56 441587

CDS 441587..442609

/gene="TTHA0472"

/inference="non-experimental evidence, no additional

details recorded"

/note="similar to GB:AAS80449.1 percent identity 99 in 340

aa"

/product="peptide ABC transporter, ATP-binding protein"

/protein_id="BAD70295.1"

**A** 0.92 0.342 0.98 0.350 0.90 0.379 0.84 0.406 0.053 326 92.94 442606

CDS 442606..443589

/gene="TTHA0473"

/inference="non-experimental evidence, no additional

details recorded"

/note="similar to GB:AAS80450.1 percent identity 99 in 327

aa"

/product="peptide ABC transporter, ATP-binding protein"

/protein_id="BAD70296.1"

**A** 0.90 0.612 0.94 0.654 0.89 0.688 0.85 0.717 0.249 131 67.94 464647

CDS complement(464249..464647)

/gene="TTHA0498"

/inference="non-experimental evidence, no additional

details recorded"

/note="similar to GB:AAN50079.1 percent identity 33 in 125

aa"

/product="conserved hypothetical protein"

/protein_id="BAD70321.1"

**A** 1.00 0.458 1.03 0.446 1.00 0.459 0.97 0.472 0.121 232 91.38 473046

CDS 473046..473747

/gene="TTHA0508"

/inference="non-experimental evidence, no additional

details recorded"

/note="similar to GB:AAS80480.1 percent identity 99 in 233

aa"

/product="transcriptional regulator, MerR family"

/protein_id="BAD70331.1"

**A** 0.93 0.453 0.98 0.462 0.92 0.490 0.87 0.523 0.046 102 89.22 510962

CDS 510962..511273

/gene="TTHA0547"

/inference="non-experimental evidence, no additional

details recorded"

/note="similar to GB:AAS80527.1 percent identity 98 in 103

aa"

/product="hypothetical protein"

/protein_id="BAD70370.1"

**A** 0.93 0.530 0.98 0.540 0.93 0.567 0.84 0.630 0.170 148 77.70 548793

CDS 548793..549242

/gene="TTHA0581"

/inference="non-experimental evidence, no additional

details recorded"

/note="similar to PDB:1BKG percent identity 33 in 168 aa"

/product="hypothetical protein"

/protein_id="BAD70404.1"

**A** 0.81 0.373 0.86 0.433 0.82 0.457 0.72 0.517 0.114 328 79.57 574551

CDS 574551..575540

/gene="TTHA0607"

/inference="non-experimental evidence, no additional

details recorded"

/note="similar to GB:AAS80590.1 percent identity 99 in 329

aa"

/product="biotin synthase (biotin synthetase)"

/protein_id="BAD70430.1"

**A** 0.93 0.594 0.99 0.597 0.90 0.658 0.85 0.696 0.215 123 79.67 599595

CDS complement(599221..599595)

/gene="TTHA0627"

/inference="non-experimental evidence, no additional

details recorded"

/note="similar to GB:AAS80609.1 percent identity 100 in

124 aa"

/product="large-conductance mechanosensitive channel"

/protein_id="BAD70450.1"

**A** 0.88 0.573 0.94 0.607 0.87 0.660 0.80 0.718 0.222 136 71.32 608062

CDS complement(607649..608062)

/gene="TTHA0640"

/inference="non-experimental evidence, no additional

details recorded"

/note="similar to PDB:1BKG percent identity 53 in 121 aa"

/product="conserved hypothetical protein"

/protein_id="BAD70463.1"

**A** 0.93 0.936 0.95 0.986 0.92 1.017 0.89 1.047 0.688 400 50.50 613513

CDS complement(612308..613513)

/gene="TTHA0644"

/inference="non-experimental evidence, no additional

details recorded"

/note="similar to PDB:1BKG percent identity 23 in 377 aa"

/product="hypothetical protein"

/protein_id="BAD70467.1"

**A** 0.89 0.692 0.91 0.758 0.89 0.780 0.84 0.826 0.435 336 60.71 614523

CDS complement(613510..614523)

/gene="TTHA0645"

/inference="non-experimental evidence, no additional

details recorded"

/note="similar to EMB:CAG37941.1 percent identity 32 in

257 aa"

/product="putative glycosyltransferase"

/protein_id="BAD70468.1"

**A** 0.96 0.641 0.98 0.653 0.99 0.651 0.90 0.711 0.232 104 67.31 614581

CDS 614581..614898

/gene="TTHA0646"

/inference="non-experimental evidence, no additional

details recorded"

/note="similar to EMB:CAB78869.1 percent identity 38 in 72

aa"

/product="hypothetical protein"

/protein_id="BAD70469.1"

**A** 0.91 0.744 0.94 0.792 0.90 0.825 0.86 0.863 0.490 373 59.25 616040

CDS complement(614916..616040)

/gene="TTHA0647"

/inference="non-experimental evidence, no additional

details recorded"

/note="similar to PDB:1BKG percent identity 51 in 234 aa"

/product="putative glycosyltransferase"

/protein_id="BAD70470.1"

**A** 0.87 0.587 0.91 0.645 0.87 0.675 0.80 0.731 0.339 370 67.03 617146

CDS complement(616031..617146)

/gene="TTHA0648"

/inference="non-experimental evidence, no additional

details recorded"

/note="similar to EMB:CAD86181.1 percent identity 32 in

378 aa"

/product="probable glycosyltransferase"

/protein_id="BAD70471.1"

**A** 0.81 0.358 0.85 0.420 0.80 0.446 0.75 0.478 0.118 420 78.57 618418

CDS complement(617153..618418)

/gene="TTHA0649"

/inference="non-experimental evidence, no additional

details recorded"

/note="similar to GB:AAB84853.1 percent identity 30 in 373

aa"

/product="putative O-antigen transporter"

/protein_id="BAD70472.1"

**A** 0.74 0.353 0.75 0.474 0.78 0.454 0.68 0.520 0.107 311 86.17 641845

CDS 641845..642783

/gene="TTHA0677"

/inference="non-experimental evidence, no additional

details recorded"

/note="similar to GB:AAS80666.1 percent identity 99 in 312

aa"

/product="putative oxidoreductase"

/protein_id="BAD70500.1"

**A** 0.97 0.431 0.96 0.448 1.00 0.430 0.97 0.443 0.041 137 84.67 650720

CDS complement(650304..650720)

/gene="TTHA0687"

/inference="non-experimental evidence, no additional

details recorded"

/note="similar to GB:AAS80675.1 percent identity 100 in

107 aa"

/product="sugar ABC transporter, permease protein

[N-terminal]"

/protein_id="BAD70510.1"

**A** 0.92 0.461 0.94 0.489 0.89 0.515 0.90 0.513 0.047 90 83.33 664320

CDS complement(664045..664320)

/gene="TTHA0700"

/inference="non-experimental evidence, no additional

details recorded"

/note="similar to GB:AAS80696.1 percent identity 100 in 91

aa"

/product="conserved hypothetical protein"

/protein_id="BAD70523.1"

**A** 0.82 0.349 0.89 0.390 0.82 0.423 0.70 0.497 0.139 683 80.97 669093

CDS 669093..671147

/gene="TTHA0706"

/inference="non-experimental evidence, no additional

details recorded"

/note="similar to GB:AAS80702.1 percent identity 89 in 684

aa"

/product="cation-transporting ATPase"

/protein_id="BAD70529.1"

**A** 0.88 0.572 0.92 0.620 0.87 0.655 0.81 0.702 0.333 433 69.52 686268

CDS 686268..687572

/gene="TTHA0724"

/inference="non-experimental evidence, no additional

details recorded"

/note="similar to GB:AAS80720.1 percent identity 53 in 458

aa"

/product="serine protease"

/protein_id="BAD70547.1"

**A** 0.92 0.387 0.99 0.393 0.86 0.448 0.86 0.451 0.074 236 79.66 724474

CDS complement(723761..724474)

/gene="TTHA0757"

/inference="non-experimental evidence, no additional

details recorded"

/note="similar to GB:AAS80753.1 percent identity 99 in 237

aa"

/product="hypothetical protein"

/protein_id="BAD70580.1"

**A** 0.89 0.373 0.95 0.394 0.89 0.418 0.78 0.477 0.102 360 79.72 732580

CDS 732580..733665

/gene="TTHA0766"

/inference="non-experimental evidence, no additional

details recorded"

/note="similar to GB:AAS80762.1 percent identity 97 in 361

aa"

/product="ABC transporter, solute-binding protein"

/protein_id="BAD70589.1"

**A** 0.79 0.312 0.83 0.376 0.77 0.406 0.74 0.421 0.075 436 85.78 734172

CDS 734172..735485

/gene="TTHA0768"

/inference="non-experimental evidence, no additional

details recorded"

/note="similar to GB:AAS80764.1 percent identity 100 in

437 aa"

/product="putative large integral membrane transport

protein"

/protein_id="BAD70591.1"

**A** 0.84 0.349 0.88 0.396 0.85 0.411 0.75 0.464 0.126 558 77.06 740674

CDS complement(738995..740674)

/gene="TTHA0771"

/inference="non-experimental evidence, no additional

details recorded"

/note="similar to GB:AAS82028.1 percent identity 43 in 386

aa"

/product="hypothetical protein"

/protein_id="BAD70594.1"

**A** 0.84 0.541 0.88 0.615 0.82 0.656 0.80 0.680 0.201 137 79.56 744055

CDS 744055..744471

/gene="TTHA0776"

/inference="non-experimental evidence, no additional

details recorded"

/note="similar to GB:AAS82025.1 percent identity 37 in 126

aa"

/product="hypothetical protein"

/protein_id="BAD70599.1"

**A** 0.92 0.577 0.95 0.606 0.95 0.610 0.86 0.673 0.232 171 87.13 841486

CDS complement(840968..841486)

/gene="TTHA0883"

/inference="non-experimental evidence, no additional

details recorded"

/note="similar to GB:AAS82429.1 percent identity 100 in

172 aa"

/product="probable transposase"

/protein_id="BAD70706.1"

**A** 0.95 0.817 0.98 0.835 0.95 0.860 0.90 0.911 0.415 99 59.60 872255

CDS complement(871956..872255)

/gene="TTHA0913"

/inference="non-experimental evidence, no additional

details recorded"

/note="similar to GB:AAS82439.1 percent identity 60 in 28

aa"

/product="hypothetical protein"

/protein_id="BAD70736.1"

**A** 0.89 0.698 0.91 0.765 0.88 0.790 0.84 0.832 0.388 193 65.80 872395

CDS complement(871811..872395)

/gene="TTHA0911"

/inference="non-experimental evidence, no additional

details recorded"

/note="similar to GB:AAS82439.1 percent identity 100 in 13

aa"

/product="hypothetical protein"

/protein_id="BAD70734.1"

**A** 1.01 0.788 1.05 0.751 0.98 0.802 0.96 0.823 0.361 98 60.20 872838

CDS complement(872539..872838)

/gene="TTHA0917"

/inference="non-experimental evidence, no additional

details recorded"

/note="similar to GB:AAS82439.1 percent identity 63 in 65

aa"

/product="hypothetical protein"

/protein_id="BAD70740.1"

**A** 0.88 0.570 0.90 0.631 0.87 0.655 0.85 0.670 0.284 250 69.20 891422

CDS complement(890667..891422)

/gene="TTHA0943"

/inference="non-experimental evidence, no additional

details recorded"

/note="similar to GB:AAS80922.1 percent identity 45 in 250

aa"

/product="conserved hypothetical protein"

/protein_id="BAD70766.1"

**A** 0.88 0.491 0.93 0.526 0.88 0.560 0.80 0.616 0.219 318 73.27 892633

CDS complement(891674..892633)

/gene="TTHA0944"

/inference="non-experimental evidence, no additional

details recorded"

/note="similar to GB:AAS80924.1 percent identity 99 in 319

aa"

/product="hypothetical protein"

/protein_id="BAD70767.1"

**A** 0.90 0.542 0.94 0.573 0.90 0.604 0.83 0.651 0.168 122 73.77 893096

CDS complement(892725..893096)

/gene="TTHA0945"

/inference="non-experimental evidence, no additional

details recorded"

/note="similar to GB:AAS80927.1 percent identity 92 in 41

aa"

/product="hypothetical protein"

/protein_id="BAD70768.1"

**A** 0.84 0.383 0.89 0.430 0.83 0.461 0.76 0.507 0.056 176 86.93 909204

CDS 909204..909737

/gene="TTHA0963"

/inference="non-experimental evidence, no additional

details recorded"

/note="similar to GB:AAS80947.1 percent identity 98 in 134

aa"

/product="ABC tranpsorter ATP binding protein related

protein"

/protein_id="BAD70786.1"

**A** 0.87 0.471 0.91 0.517 0.87 0.540 0.80 0.589 0.122 145 78.62 936513

CDS 936513..936953

/gene="TTHA0990"

/inference="non-experimental evidence, no additional

details recorded"

/note="similar to GB:AAS80976.1 percent identity 86 in 75

aa"

/product="hypothetical protein"

/protein_id="BAD70813.1"

**A** 1.01 0.713 1.04 0.683 1.00 0.715 0.97 0.735 0.287 103 66.99 955973

CDS 955973..956287

/gene="TTHA1009"

/inference="non-experimental evidence, no additional

details recorded"

/note="similar to GB:AAR36113.1 percent identity 48 in 70

aa"

/product="conserved hypothetical protein"

/protein_id="BAD70832.1"

**A** 0.90 0.534 0.94 0.567 0.87 0.613 0.85 0.631 0.139 97 77.32 961734

CDS complement(961438..961734)

/gene="TTHA1015"

/inference="non-experimental evidence, no additional

details recorded"

/note="similar to GB:AAM32305.1 percent identity 53 in 84

aa"

/product="probable nucleotidyltransferase"

/protein_id="BAD70838.1"

**A** 0.86 0.266 0.94 0.284 0.88 0.304 0.73 0.365 0.039 913 83.46 964535

CDS complement(961791..964535)

/gene="TTHA1016"

/inference="non-experimental evidence, no additional

details recorded"

/note="similar to PDB:1BKG percent identity 35 in 958 aa"

/product="conserved hypothetical protein"

/protein_id="BAD70839.1"

**A** 0.88 0.495 0.93 0.531 0.86 0.573 0.80 0.620 0.255 450 73.11 966050

CDS complement(964695..966050)

/gene="TTHA1017"

/inference="non-experimental evidence, no additional

details recorded"

/note="similar to GB:AAM71914.1 percent identity 67 in 452

aa"

/product="conserved hypothetical protein"

/protein_id="BAD70840.1"

**A** 0.85 0.447 0.91 0.492 0.83 0.535 0.77 0.579 0.203 405 81.48 967294

CDS complement(966074..967294)

/gene="TTHA1018"

/inference="non-experimental evidence, no additional

details recorded"

/note="similar to DBJ:BAC55317.1 percent identity 99 in

405 aa"

/product="transposase"

/protein_id="BAD70841.1"

**A** 0.96 0.789 1.00 0.790 0.94 0.842 0.91 0.864 0.401 120 60.00 967652

CDS complement(967287..967652)

/gene="TTHA1019"

/inference="non-experimental evidence, no additional

details recorded"

/note="similar to GB:AAM71914.1 percent identity 60 in 109

aa"

/product="conserved hypothetical protein"

/protein_id="BAD70842.1"

**A** 1.01 0.476 1.04 0.458 0.95 0.501 1.02 0.467 0.057 110 92.73 973272

CDS 973272..973607

/gene="TTHA1023"

/inference="non-experimental evidence, no additional

details recorded"

/note="similar to GB:AAS81006.1 percent identity 92 in 111

aa"

/product="hypothetical protein"

/protein_id="BAD70846.1"

**A** 0.86 0.458 0.87 0.523 0.80 0.571 0.89 0.515 0.108 143 81.12 995832

CDS 995832..996266

/gene="TTHA1052"

/inference="non-experimental evidence, no additional

details recorded"

/note="similar to GB:AAS82146.1 percent identity 39 in 130

aa"

/product="conserved hypothetical protein"

/protein_id="BAD70875.1"

**A** 0.88 0.447 0.87 0.515 0.93 0.481 0.85 0.527 0.032 80 90.00 1024439

CDS 1024439..1024684

/gene="TTHA1077"

/inference="non-experimental evidence, no additional

details recorded"

/note="similar to GB:AAS81060.1 percent identity 98 in 81

aa"

/product="hypothetical protein"

/protein_id="BAD70900.1"

**A** 0.74 0.343 0.80 0.428 0.73 0.467 0.66 0.520 0.102 334 87.13 1055670

CDS 1055670..1056677

/gene="TTHA1109"

/inference="non-experimental evidence, no additional

details recorded"

/note="similar to GB:AAS81091.1 percent identity 100 in

152 aa"

/product="putative glycosyltransferase"

/protein_id="BAD70932.1"

**A** 0.96 0.524 1.02 0.513 0.96 0.548 0.86 0.606 0.162 158 79.11 1097569

CDS complement(1097090..1097569)

/gene="TTHA1151"

/inference="non-experimental evidence, no additional

details recorded"

/note="similar to GB:AAS81134.1 percent identity 99 in 408

aa"

/product="hypothetical protein"

/protein_id="BAD70974.1"

**A** 0.94 0.509 0.98 0.520 0.94 0.544 0.87 0.583 0.112 112 76.79 1145132

CDS complement(1144791..1145132)

/gene="TTHA1201"

/inference="non-experimental evidence, no additional

details recorded"

/note="similar to GB:AAS81186.1 percent identity 91 in 113

aa"

/product="conserved hypothetical protein"

/protein_id="BAD71024.1"

**A** 0.95 0.423 0.99 0.429 0.95 0.447 0.88 0.482 0.068 174 82.76 1151330

CDS complement(1150803..1151330)

/gene="TTHA1209"

/inference="non-experimental evidence, no additional

details recorded"

/note="similar to GB:AAS81192.1 percent identity 98 in 175

aa"

/product="probable acetyltransferase"

/protein_id="BAD71032.1"

**A** 0.90 0.406 0.95 0.426 0.89 0.455 0.80 0.508 0.054 154 83.12 1158001

CDS 1158001..1158468

/gene="TTHA1217"

/inference="non-experimental evidence, no additional

details recorded"

/note="similar to GB:AAM55482.1 percent identity 36 in 163

aa"

/product="prepilin-like protein"

/protein_id="BAD71040.1"

**A** 0.94 0.436 0.97 0.449 0.95 0.457 0.88 0.498 0.044 122 77.87 1158455

CDS 1158455..1158826

/gene="TTHA1218"

/inference="non-experimental evidence, no additional

details recorded"

/note="similar to EMB:CAG69992.1 percent identity 35 in 62

aa"

/product="probable prepilin-like protein"

/protein_id="BAD71041.1"

**A** 0.82 0.370 0.87 0.426 0.79 0.469 0.75 0.491 0.081 235 80.43 1158823

CDS 1158823..1159533

/gene="TTHA1219"

/inference="non-experimental evidence, no additional

details recorded"

/note="similar to PDB:1BKG percent identity 29 in 105 aa"

/product="probable general secretion pathway protein J"

/protein_id="BAD71042.1"

**A** 0.89 0.462 0.93 0.499 0.88 0.523 0.84 0.550 0.084 121 76.86 1161319

CDS 1161319..1161687

/gene="TTHA1221"

/inference="non-experimental evidence, no additional

details recorded"

/note="similar to GB:AAF10127.1 percent identity 45 in 143

aa"

/product="pilin, type IV, putative"

/protein_id="BAD71044.1"

**A** 0.92 0.580 0.97 0.596 0.88 0.657 0.86 0.673 0.203 122 68.03 1161746

CDS 1161746..1162117

/gene="TTHA1222"

/inference="non-experimental evidence, no additional

details recorded"

/note="similar to GB:AAF10127.1 percent identity 43 in 66

aa"

/product="pilin, type IV, putative"

/protein_id="BAD71045.1"

**A** 1.03 0.367 1.09 0.337 0.99 0.370 0.96 0.381 0.039 252 86.11 1162310

CDS 1162310..1163071

/gene="TTHA1223"

/inference="non-experimental evidence, no additional

details recorded"

/note="similar to GB:AAS81203.1 percent identity 76 in 170

aa"

/product="conserved hypothetical protein"

/protein_id="BAD71046.1"

**A** 0.91 0.626 0.95 0.657 0.90 0.693 0.85 0.741 0.325 232 67.24 1163147

CDS 1163147..1163848

/gene="TTHA1224"

/inference="non-experimental evidence, no additional

details recorded"

/note="similar to GB:AAS81204.1 percent identity 96 in 86

aa"

/product="conserved hypothetical protein"

/protein_id="BAD71047.1"

**A** 0.84 0.439 0.90 0.489 0.83 0.530 0.76 0.575 0.198 405 80.99 1211008

CDS 1211008..1212228

/gene="TTHA1269"

/inference="non-experimental evidence, no additional

details recorded"

/note="similar to GB:AAS81248.1 percent identity 100 in

197 aa"

/product="transposase"

/protein_id="BAD71092.1"

**A** 0.77 0.435 0.82 0.531 0.75 0.582 0.71 0.611 0.244 557 80.07 1233754

CDS complement(1232078..1233754)

/gene="TTHA1290"

/inference="non-experimental evidence, no additional

details recorded"

/note="similar to GB:AAS81268.1 percent identity 100 in 89

aa"

/product="putative membrane protein"

/protein_id="BAD71113.1"

**A** 0.89 0.499 0.93 0.534 0.89 0.559 0.83 0.603 0.116 113 76.99 1237694

CDS complement(1237350..1237694)

/gene="TTHA1297"

/inference="non-experimental evidence, no additional

details recorded"

/note="similar to GB:AAS81274.1 percent identity 100 in

114 aa"

/product="hypothetical protein"

/protein_id="BAD71120.1"

**A** 0.86 0.458 0.91 0.506 0.83 0.551 0.81 0.562 0.079 109 84.40 1247243

CDS complement(1246911..1247243)

/gene="TTHA1307"

/inference="non-experimental evidence, no additional

details recorded"

/note="similar to GB:AAS81283.1 percent identity 99 in 110

aa"

/product="hypothetical protein"

/protein_id="BAD71130.1"

**A** 0.95 0.960 0.98 0.983 0.94 1.020 0.89 1.075 0.642 207 45.89 1273732

CDS 1273732..1274358

/gene="TTHA1333"

/inference="non-experimental evidence, no additional

details recorded"

/note="similar to GB:AAK61383.1 percent identity 38 in 175

aa"

/product="hypothetical protein"

/protein_id="BAD71156.1"

**A** 0.90 0.558 0.93 0.601 0.90 0.620 0.84 0.664 0.288 336 69.35 1302641

CDS complement(1301628..1302641)

/gene="TTHA1364"

/inference="non-experimental evidence, no additional

details recorded"

/note="similar to GB:AAS81341.1 percent identity 95 in 337

aa"

/product="conserved hypothetical protein"

/protein_id="BAD71187.1"

**A** 0.89 0.295 0.93 0.318 0.90 0.329 0.81 0.363 0.039 467 84.58 1312180

CDS 1312180..1313586

/gene="TTHA1376"

/inference="non-experimental evidence, no additional

details recorded"

/note="similar to GB:AAS81352.1 percent identity 90 in 464

aa"

/product="HD domain protein"

/protein_id="BAD71199.1"

**A** 0.92 0.283 0.88 0.323 0.96 0.295 0.96 0.295 0.031 547 90.68 1358638

CDS 1358638..1360284

/gene="TTHA1430"

/inference="non-experimental evidence, no additional

details recorded"

/note="similar to GB:AAS81407.1 percent identity 99 in 548

aa"

/product="long-chain fatty acid--CoA ligase"

/protein_id="BAD71253.1"

**A** 0.98 0.299 0.93 0.319 0.99 0.302 1.09 0.274 0.038 559 89.98 1389147

CDS 1389147..1390829

/gene="TTHA1463"

/inference="non-experimental evidence, no additional

details recorded"

/note="similar to GB:AAS81441.1 percent identity 95 in 560

aa"

/product="long-chain-fatty-acid--CoA ligase"

/protein_id="BAD71286.1"

**A** 0.98 0.436 1.04 0.421 1.00 0.435 0.86 0.505 0.052 139 89.21 1409578

CDS complement(1409156..1409578)

/gene="TTHA1481"

/inference="non-experimental evidence, no additional

details recorded"

/note="similar to GB:AAS81459.1 percent identity 98 in 140

aa"

/product="thioredoxin"

/protein_id="BAD71304.1"

**A** 0.82 0.387 0.87 0.444 0.85 0.457 0.73 0.532 0.092 225 87.56 1435887

CDS complement(1435207..1435887)

/gene="TTHA1507"

/inference="non-experimental evidence, no additional

details recorded"

/note="similar to GB:AAS81485.1 percent identity 98 in 225

aa"

/product="probable uroporphyrinogen III synthase"

/protein_id="BAD71330.1"

**A** 0.94 0.563 1.01 0.558 0.90 0.629 0.86 0.655 0.151 89 82.02 1438352

CDS complement(1438080..1438352)

/gene="TTHA1512"

/inference="non-experimental evidence, no additional

details recorded"

/note="similar to GB:AAS81490.1 percent identity 100 in 90

aa"

/product="putative nucleotidyltransferase"

/protein_id="BAD71335.1"

**A** 0.87 0.479 0.90 0.531 0.86 0.555 0.82 0.586 0.191 249 71.49 1458300

CDS 1458300..1459052

/gene="TTHA1533"

/inference="non-experimental evidence, no additional

details recorded"

/note="similar to GB:AAM43200.1 percent identity 35 in 256

aa"

/product="hypothetical protein"

/protein_id="BAD71356.1"

**A** 0.82 0.346 0.86 0.405 0.82 0.423 0.77 0.452 0.121 533 82.55 1464883

CDS complement(1463279..1464883)

/gene="TTHA1537"

/inference="non-experimental evidence, no additional

details recorded"

/note="similar to GB:AAS80793.1 percent identity 90 in 534

aa"

/product="conserved hypothetical protein"

/protein_id="BAD71360.1"

**A** 0.94 0.616 0.99 0.624 0.94 0.653 0.85 0.726 0.326 292 78.08 1465735

CDS 1465735..1466616

/gene="TTHA1539"

/inference="non-experimental evidence, no additional

details recorded"

/note="similar to PDB:1BKG percent identity 33 in 294 aa"

/product="putative phage integrase/recombinase"

/protein_id="BAD71362.1"

**A** 0.94 0.588 0.97 0.609 0.93 0.631 0.90 0.653 0.249 190 73.68 1467166

CDS complement(1466591..1467166)

/gene="TTHA1540"

/inference="non-experimental evidence, no additional

details recorded"

/note="similar to EMB:CAA68552.1 percent identity 57 in 26

aa"

/product="hypothetical protein"

/protein_id="BAD71363.1"

HA 1.11 0.783 1.14 0.685 1.11 0.706 1.04 0.750 0.338 119 81.51 1467466

CDS 1467466..1467828

/gene="TTHA1541"

/inference="non-experimental evidence, no additional

details recorded"

/note="similar to PDB:1BKG percent identity 35 in 109 aa"

/product="hypothetical protein"

/protein_id="BAD71364.1"

**A** 0.82 0.453 0.86 0.530 0.82 0.555 0.77 0.587 0.221 427 74.47 1506201

CDS 1506201..1507487

/gene="TTHA1583"

/inference="non-experimental evidence, no additional

details recorded"

/note="similar to PIR:JH0634 percent identity 99 in 428

aa"

/product="site-specific DNA-methyltransferase

(adenine-specific) TthHB8I"

/protein_id="BAD71406.1"

**A** 0.93 0.753 0.95 0.793 0.92 0.818 0.90 0.840 0.461 262 58.40 1507447

CDS 1507447..1508238

/gene="TTHA1584"

/inference="non-experimental evidence, no additional

details recorded"

/note="similar to GB:AAA27490.1 percent identity 99 in 263

aa"

/product="Type II restriction enzyme TthHB8I (endonuclease

TthHB8I) (R.TthHB8I)"

/protein_id="BAD71407.1"

**A** 0.88 0.434 0.93 0.464 0.86 0.502 0.79 0.548 0.054 114 78.95 1562305

CDS complement(1561958..1562305)

/gene="TTHA1646"

/inference="non-experimental evidence, no additional

details recorded"

/note="similar to GB:AAP42871.1 percent identity 31 in 108

aa"

/product="hypothetical protein"

/protein_id="BAD71469.1"

**A** 0.92 0.536 0.94 0.567 0.92 0.581 0.89 0.605 0.127 94 80.85 1564469

CDS complement(1564182..1564469)

/gene="TTHA1649"

/inference="non-experimental evidence, no additional

details recorded"

/note="similar to GB:AAS81627.1 percent identity 100 in 95

aa"

/product="nucleotidyltransferase"

/protein_id="BAD71472.1"

**A** 0.88 0.393 0.95 0.413 0.84 0.469 0.80 0.488 0.038 146 83.56 1571531

CDS complement(1571088..1571531)

/gene="TTHA1656"

/inference="non-experimental evidence, no additional

details recorded"

/note="similar to GB:AAS81634.1 percent identity 99 in 147

aa"

/product="hypothetical protein"

/protein_id="BAD71479.1"

**A** 0.98 0.703 1.01 0.695 0.98 0.720 0.93 0.752 0.425 405 84.44 1592316

CDS complement(1591096..1592316)

/gene="TTHA1694"

/inference="non-experimental evidence, no additional

details recorded"

/note="similar to EMB:CAA29856.1 percent identity 100 in

406 aa"

/product="elongation factor Tu (EF-Tu)"

/protein_id="BAD71517.1"

**A** 0.80 0.345 0.83 0.416 0.77 0.451 0.76 0.452 0.071 270 80.74 1620596

CDS 1620596..1621411

/gene="TTHA1727"

/inference="non-experimental evidence, no additional

details recorded"

/note="similar to GB:EAL13330.1 percent identity 34 in 230

aa"

/product="conserved hypothetical protein"

/protein_id="BAD71550.1"

**A** 0.92 0.390 0.96 0.406 0.88 0.445 0.88 0.442 0.042 175 77.71 1621420

CDS 1621420..1621950

/gene="TTHA1728"

/inference="non-experimental evidence, no additional

details recorded"

/note="similar to GB:AAS81379.1 percent identity 61 in 177

aa"

/product="conserved hypothetical protein"

/protein_id="BAD71551.1"

**A** 0.94 0.471 1.02 0.462 0.86 0.546 0.87 0.540 0.048 80 78.75 1622660

CDS 1622660..1622905

/gene="TTHA1730"

/inference="non-experimental evidence, no additional

details recorded"

/note="similar to GB:AAS81709.1 percent identity 100 in 79

aa"

/product="conserved hypothetical protein"

/protein_id="BAD71553.1"

**A** 0.75 0.295 0.78 0.377 0.76 0.387 0.67 0.441 0.058 391 81.07 1646104

CDS 1646104..1647282

/gene="TTHA1758"

/inference="non-experimental evidence, no additional

details recorded"

/note="similar to GB:AAS81739.1 percent identity 97 in 297

aa"

/product="GGDEF domain protein"

/protein_id="BAD71581.1"

**A** 0.95 0.688 0.99 0.692 0.95 0.728 0.87 0.789 0.286 101 63.37 1664247

CDS complement(1663939..1664247)

/gene="TTHA1776"

/inference="non-experimental evidence, no additional

details recorded"

/note="similar to SP:P20909 percent identity 33 in 101 aa"

/product="hypothetical protein"

/protein_id="BAD71599.1"

**A** 0.87 0.550 0.90 0.614 0.87 0.629 0.81 0.679 0.257 229 85.15 1683043

CDS complement(1682354..1683043)

/gene="TTHA1801"

/inference="non-experimental evidence, no additional

details recorded"

/note="similar to GB:AAS81959.1 percent identity 99 in 229

aa"

/product="hypothetical protein"

/protein_id="BAD71624.1"

**A** 0.92 0.490 0.98 0.502 0.91 0.541 0.84 0.585 0.232 424 76.42 1732434

CDS 1732434..1733711

/gene="TTHA1848"

/inference="non-experimental evidence, no additional

details recorded"

/note="similar to GB:AAA51770.1 percent identity 63 in 41

aa"

/product="hypothetical protein"

/protein_id="BAD71671.1"

**A** 0.96 0.310 1.02 0.303 0.89 0.349 0.93 0.333 0.037 438 84.93 1746121

CDS 1746121..1747440

/gene="TTHA1864"

/inference="non-experimental evidence, no additional

details recorded"

/note="similar to GB:AAS81874.1 percent identity 30 in 288

aa"

/product="S-layer protein-related protein"

/protein_id="BAD71687.1"

**A** 0.81 0.381 0.84 0.452 0.81 0.473 0.74 0.512 0.152 456 80.48 1748822

CDS complement(1747449..1748822)

/gene="TTHA1865"

/inference="non-experimental evidence, no additional

details recorded"

/note="similar to GB:AAO26021.1 percent identity 33 in 476

aa"

/product="serine protease precursor"

/protein_id="BAD71688.1"

**A** 0.76 0.351 0.81 0.435 0.75 0.468 0.69 0.511 0.127 422 85.07 1750090

CDS complement(1748819..1750090)

/gene="TTHA1866"

/inference="non-experimental evidence, no additional

details recorded"

/note="similar to GB:AAF12643.1 percent identity 26 in 258

aa"

/product="hypothetical protein"

/protein_id="BAD71689.1"

**A** 0.84 0.439 0.90 0.489 0.83 0.530 0.76 0.575 0.198 405 80.99 1750258

CDS 1750258..1751478

/gene="TTHA1867"

/inference="non-experimental evidence, no additional

details recorded"

/note="similar to DBJ:BAC55317.1 percent identity 99 in

406 aa"

/product="transposase"

/protein_id="BAD71690.1"

**A** 0.95 0.556 0.98 0.567 0.96 0.581 0.88 0.635 0.165 120 73.33 1754254

CDS complement(1753889..1754254)

/gene="TTHA1869"

/inference="non-experimental evidence, no additional

details recorded"

/note="similar to DBJ:BAC17422.1 percent identity 25 in

125 aa"

/product="hypothetical protein"

/protein_id="BAD71692.1"

**A** 0.92 0.411 0.95 0.432 0.94 0.438 0.84 0.490 0.056 162 83.95 1754971

CDS complement(1754480..1754971)

/gene="TTHA1870"

/inference="non-experimental evidence, no additional

details recorded"

/note="similar to GB:AAP79133.1 percent identity 34 in 84

aa"

/product="hypothetical protein"

/protein_id="BAD71693.1"

**A** 0.87 0.552 0.90 0.616 0.88 0.629 0.81 0.679 0.258 229 85.59 1780897

CDS 1780897..1781586

/gene="TTHA1895"

/inference="non-experimental evidence, no additional

details recorded"

/note="similar to GB:AAS81959.1 percent identity 99 in 229

aa"

/product="hypothetical protein"

/protein_id="BAD71718.1"

**A** 1.03 0.574 1.09 0.528 1.02 0.561 0.93 0.620 0.164 122 79.51 1791445

CDS complement(1791074..1791445)

/gene="TTHA1905"

/inference="non-experimental evidence, no additional

details recorded"

/note="similar to GB:AAK41855.1 percent identity 37 in 130

aa"

/product="conserved hypothetical protein"

/protein_id="BAD71728.1"

**A** 0.82 0.425 0.86 0.496 0.84 0.507 0.74 0.570 0.051 105 84.76 1791737

CDS complement(1791417..1791737)

/gene="TTHA1906"

/inference="non-experimental evidence, no additional

details recorded"

/note="similar to PDB:1BKG percent identity 34 in 93 aa"

/product="conserved hypothetical protein"

/protein_id="BAD71729.1"

**A** 0.89 0.385 0.93 0.414 0.88 0.438 0.84 0.458 0.030 154 87.66 1824153

CDS 1824153..1824620

/gene="TTHA1947"

/inference="non-experimental evidence, no additional

details recorded"

/note="similar to GB:AAS81926.1 percent identity 92 in 151

aa"

/product="MutT/nudix family protein"

/protein_id="BAD71770.1"

**A** 0.87 0.550 0.90 0.614 0.87 0.629 0.81 0.679 0.257 229 85.15 1825398

CDS 1825398..1826087

/gene="TTHA1948"

/inference="non-experimental evidence, no additional

details recorded"

/note="similar to GB:AAS81959.1 percent identity 99 in 229

aa"

/product="hypothetical protein"

/protein_id="BAD71771.1"

**A** 0.93 0.348 0.97 0.359 0.92 0.379 0.86 0.405 0.078 414 80.19 1826158

CDS 1826158..1827405

/gene="TTHA1949"

/inference="non-experimental evidence, no additional

details recorded"

/note="similar to GB:AAM30086.1 percent identity 25 in 218

aa"

/product="hypothetical protein"

/protein_id="BAD71772.1"

LIST OF ALIEN GENES

Determined by codon bias relative to all genes and selected other standards

Standards: chromosome2CDS.cbRAll

chromosome2CDS.cbRRP

chromosome2CDS.cbRCH

chromosome2CDS.cbRTF

Number of genes: 235

Criteria: all biases > threshold depending on gene length:

0.4275(100) 0.3830(150) 0.3268(250) 0.2886(400) 0.2533(600)

Eg(Standard) = Bias(All)/Bias(Standard)

Eg = Bias(All)/[0.5*Bias(RP)+0.25*Bias(CH)+0.25*Bias(TF)]

ALIEN GENES:

Eg B(all) EgRP B(RP) EgCH B(CH) EgTF B(TF) Ag Length S3 Position

**A** 0.89 0.726 0.92 0.793 0.91 0.796 0.84 0.863 0.342 98 71.43 302

CDS complement(3..302)

/gene="TTHB001"

/inference="non-experimental evidence, no additional

details recorded"

/note="similar to GB:AAS81775.1 percent identity 100 in 60

aa"

/product="hypothetical protein"

/protein_id="BAD71797.1"

**A** 0.93 0.544 0.91 0.601 0.96 0.564 0.97 0.562 0.123 90 74.44 1033

CDS 1033..1308

/gene="TTHB003"

/inference="non-experimental evidence, no additional

details recorded"

/note="similar to EMB:CAE26382.1 percent identity 41 in 41

aa"

/product="hypothetical protein"

/protein_id="BAD71799.1"

**A** 0.90 0.571 0.94 0.610 0.91 0.629 0.82 0.694 0.237 171 85.96 1823

CDS complement(1305..1823)

/gene="TTHB004"

/inference="non-experimental evidence, no additional

details recorded"

/note="similar to GB:AAS82429.1 percent identity 100 in

172 aa"

/product="probable transposase"

/protein_id="BAD71800.1"

**A** 0.91 0.427 0.97 0.439 0.87 0.490 0.84 0.510 0.055 133 91.73 3555

CDS 3555..3959

/gene="TTHB009"

/inference="non-experimental evidence, no additional

details recorded"

/note="similar to GB:AAS82427.1 percent identity 97 in 134

aa"

/product="conserved hypothetical protein"

/protein_id="BAD71805.1"

**A** 0.87 0.366 0.93 0.394 0.85 0.429 0.80 0.457 0.050 216 77.31 5697

CDS complement(5044..5697)

/gene="TTHB012"

/inference="non-experimental evidence, no additional

details recorded"

/note="similar to REF:ZP_00245479.1 percent identity 44 in

225 aa"

/product="phosphoglycerate mutase family protein"

/protein_id="BAD71808.1"

**A** 0.86 0.434 0.90 0.482 0.86 0.503 0.78 0.556 0.154 275 80.00 15489

CDS 15489..16319

/gene="TTHB024"

/inference="non-experimental evidence, no additional

details recorded"

/note="similar to GB:AAS82410.1 percent identity 80 in 315

aa"

/product="hypothetical protein"

/protein_id="BAD71821.1"

**A** 0.90 0.364 0.94 0.388 0.84 0.433 0.88 0.416 0.068 276 90.58 38945

CDS complement(38112..38945)

/gene="TTHB045"

/inference="non-experimental evidence, no additional

details recorded"

/note="similar to GB:AAS82333.1 percent identity 78 in 170

aa"

/product="repeat motif-containing protein"

/protein_id="BAD71841.1"

**A** 0.78 0.388 0.81 0.479 0.79 0.491 0.72 0.539 0.077 176 86.36 42538

CDS 42538..43071

/gene="TTHB049"

/inference="non-experimental evidence, no additional

details recorded"

/note="similar to GB:AAS82336.1 percent identity 99 in 177

aa"

/product="alpha-ribazole-5'-phosphate phosphatase"

/protein_id="BAD71845.1"

**A** 0.74 0.336 0.79 0.423 0.72 0.464 0.68 0.498 0.070 246 86.59 47663

CDS 47663..48406

/gene="TTHB055"

/inference="non-experimental evidence, no additional

details recorded"

/note="similar to GB:AAS82342.1 percent identity 100 in

247 aa"

/product="precorrin-4 C11-methyltransferase"

/protein_id="BAD71851.1"

**A** 0.93 0.496 0.99 0.501 0.93 0.531 0.82 0.609 0.131 142 78.17 54005

CDS 54005..54436

/gene="TTHB063"

/inference="non-experimental evidence, no additional

details recorded"

/note="similar to GB:AAS82350.1 percent identity 100 in

139 aa"

/product="conserved hypothetical protein"

/protein_id="BAD71859.1"

**A** 0.76 0.364 0.80 0.454 0.72 0.508 0.74 0.494 0.073 206 83.01 64037

CDS 64037..64660

/gene="TTHB072"

/inference="non-experimental evidence, no additional

details recorded"

/note="similar to GB:AAS82360.1 percent identity 98 in 160

aa"

/product="4-hydroxy-2-oxoglutarate

aldolase/2-deydro-3-deoxyphosphogluconate aldolase"

/protein_id="BAD71868.1"

**A** 0.84 0.439 0.90 0.489 0.83 0.530 0.76 0.575 0.198 405 80.99 75983

CDS complement(74763..75983)

/gene="TTHB084"

/inference="non-experimental evidence, no additional

details recorded"

/note="similar to DBJ:BAC55317.1 percent identity 99 in

406 aa"

/product="transposase"

/protein_id="BAD71880.1"

**A** 0.88 0.452 0.86 0.527 0.91 0.496 0.90 0.502 0.040 85 85.88 95716

CDS 95716..95976

/gene="TTHB107"

/inference="non-experimental evidence, no additional

details recorded"

/note="similar to GB:AAS82394.1 percent identity 100 in 80

aa"

/product="conserved hypothetical protein"

/protein_id="BAD71903.1"

**A** 0.86 0.411 0.85 0.484 0.90 0.459 0.86 0.479 0.041 129 83.72 99060

CDS 99060..99452

/gene="TTHB111"

/inference="non-experimental evidence, no additional

details recorded"

/note="similar to GB:AAS82398.1 percent identity 99 in 130

aa"

/product="conserved hypothetical protein"

/protein_id="BAD71907.1"

**A** 0.84 0.417 0.88 0.475 0.84 0.498 0.79 0.527 0.065 138 83.33 105746

CDS 105746..106165

/gene="TTHB117"

/inference="non-experimental evidence, no additional

details recorded"

/note="similar to GB:AAS82405.1 percent identity 97 in 139

aa"

/product="putative type IV pilin"

/protein_id="BAD71913.1"

**A** 0.90 0.469 0.94 0.498 0.90 0.524 0.84 0.559 0.062 90 80.00 111400

CDS complement(111125..111400)

/gene="TTHB123"

/inference="non-experimental evidence, no additional

details recorded"

/note="similar to GB:AAC68729.1 percent identity 26 in 89

aa"

/product="hypothetical protein"

/protein_id="BAD71919.1"

**A** 0.84 0.439 0.90 0.489 0.83 0.530 0.76 0.575 0.198 405 80.99 119658

CDS complement(118438..119658)

/gene="TTHB129"

/inference="non-experimental evidence, no additional

details recorded"

/note="similar to DBJ:BAC55317.1 percent identity 99 in

406 aa"

/product="transposase"

/protein_id="BAD71925.1"

**A** 0.94 0.714 0.99 0.724 0.94 0.759 0.86 0.828 0.360 151 64.24 143986

CDS complement(143528..143986)

/gene="TTHB153"

/inference="non-experimental evidence, no additional

details recorded"

/note="similar to GB:AAS82438.1 percent identity 100 in

152 aa"

/product="hypothetical protein"

/protein_id="BAD71949.1"

**A** 0.94 0.710 0.97 0.735 0.93 0.760 0.90 0.790 0.324 118 81.36 158494

CDS 158494..158853

/gene="TTHB167"

/inference="non-experimental evidence, no additional

details recorded"

/note="similar to GB:AAS82410.1 percent identity 97 in 113

aa"

/product="hypothetical protein"

/protein_id="BAD71963.1"

**A** 0.90 0.461 0.97 0.474 0.88 0.525 0.80 0.573 0.104 143 73.43 158902

CDS 158902..159336

/gene="TTHB168"

/inference="non-experimental evidence, no additional

details recorded"

/note="similar to PDB:1V26 percent identity 56 in 32 aa"

/product="hypothetical protein"

/protein_id="BAD71964.1"

**A** 0.86 0.380 0.91 0.417 0.89 0.429 0.74 0.512 0.044 168 83.93 196441

CDS 196441..196950

/gene="TTHB189"

/inference="non-experimental evidence, no additional

details recorded"

/note="similar to PDB:1V26 percent identity 44 in 158 aa"

/product="conserved hypothetical protein"

/protein_id="BAD71985.1"

**A** 0.93 0.449 0.97 0.463 0.92 0.490 0.87 0.518 0.062 123 82.11 200376

CDS 200376..200750

/gene="TTHB194"

/inference="non-experimental evidence, no additional

details recorded"

/note="similar to GB:AAC75796.1 percent identity 45 in 87

aa"

/product="conserved hypothetical protein"

/protein_id="BAD71990.1"

**A** 1.04 0.475 1.12 0.426 1.04 0.458 0.92 0.519 0.183 401 78.80 206364

CDS 206364..207572

/gene="TTHB198"

/inference="non-experimental evidence, no additional

details recorded"

/note="similar to GB:AAS82476.1 percent identity 88 in 402

aa"

/product="hypothetical protein"

/protein_id="BAD71994.1"

**A** 0.98 0.474 1.07 0.445 0.95 0.499 0.85 0.557 0.234 946 81.29 207574

CDS 207574..210417

/gene="TTHB199"

/inference="non-experimental evidence, no additional

details recorded"

/note="similar to GB:AAS82477.1 percent identity 95 in 857

aa"

/product="hypothetical protein"

/protein_id="BAD71995.1"

**A** 0.88 0.524 0.94 0.557 0.87 0.599 0.80 0.655 0.225 228 81.14 211812

CDS complement(211123..211812)

/gene="TTHB200"

/inference="non-experimental evidence, no additional

details recorded"

/note="similar to DBJ:BAC55317.1 percent identity 100 in

229 aa"

/product="transposase-like protein"

/protein_id="BAD71996.1"

**A** 0.92 0.512 0.99 0.518 0.88 0.579 0.83 0.613 0.178 185 77.30 212344

CDS complement(211784..212344)

/gene="TTHB201"

/inference="non-experimental evidence, no additional

details recorded"

/note="similar to DBJ:BAC55317.1 percent identity 98 in

165 aa"

/product="transposase-like protein"

/protein_id="BAD71997.1"

**A** 0.83 0.440 0.85 0.517 0.85 0.515 0.79 0.559 0.070 114 78.95 213872

CDS complement(213525..213872)

/gene="TTHB205"

/inference="non-experimental evidence, no additional

details recorded"

/note="similar to GB:AAS82487.1 percent identity 95 in 62

aa"

/product="hypothetical protein"

/protein_id="BAD72001.1"

**A** 0.87 0.550 0.90 0.614 0.87 0.629 0.81 0.679 0.257 229 85.15 214431

CDS complement(213742..214431)

/gene="TTHB206"

/inference="non-experimental evidence, no additional

details recorded"

/note="similar to GB:AAS81959.1 percent identity 99 in 229

aa"

/product="hypothetical protein"

/protein_id="BAD72002.1"

**A** 0.83 0.323 0.89 0.362 0.82 0.395 0.73 0.445 0.056 331 77.95 229756

CDS complement(228758..229756)

/gene="TTHB224"

/inference="non-experimental evidence, no additional

details recorded"

/note="similar to GB:AAS82526.1 percent identity 98 in 325

aa"

/product="conserved hypothetical protein"

/protein_id="BAD72020.1"

**A** 0.81 0.359 0.85 0.425 0.83 0.435 0.75 0.481 0.044 193 79.79 230310

CDS complement(229726..230310)

/gene="TTHB225"

/inference="non-experimental evidence, no additional

details recorded"

/note="similar to GB:AAS82527.1 percent identity 99 in 194

aa"

/product="conserved hypothetical protein"

/protein_id="BAD72021.1"

**A** 0.76 0.304 0.82 0.372 0.79 0.387 0.66 0.462 0.066 396 81.82 230466

CDS 230466..231659

/gene="TTHB226"

/inference="non-experimental evidence, no additional

details recorded"

/note="similar to GB:AAS82528.1 percent identity 98 in 378

aa"

/product="conserved hypothetical protein"

/protein_id="BAD72022.1"

**A** 0.80 0.326 0.87 0.375 0.80 0.408 0.70 0.465 0.118 765 78.43 231677

CDS 231677..233977

/gene="TTHB227"

/inference="non-experimental evidence, no additional

details recorded"

/note="similar to GB:AAS82529.1 percent identity 98 in 766

aa"

/product="hypothetical protein"

/protein_id="BAD72023.1"

**A** 0.79 0.346 0.83 0.419 0.80 0.432 0.71 0.490 0.083 303 80.20 234739

CDS 234739..235653

/gene="TTHB229"

/inference="non-experimental evidence, no additional

details recorded"

/note="similar to GB:AAS82531.1 percent identity 99 in 304

aa"

/product="hypothetical protein"

/protein_id="BAD72025.1"

**A** 0.94 0.331 1.00 0.330 0.91 0.363 0.86 0.386 0.022 263 81.37 238073

CDS 238073..238867

/gene="TTHB231"

/inference="non-experimental evidence, no additional

details recorded"

/note="similar to GB:AAS82532.1 percent identity 98 in 264

aa"

/product="conserved hypothetical protein"

/protein_id="BAD72027.1"

**A** 0.85 0.442 0.91 0.487 0.83 0.530 0.77 0.574 0.198 405 81.48 240526

CDS complement(239306..240526)

/gene="TTHB232"

/inference="non-experimental evidence, no additional

details recorded"

/note="similar to DBJ:BAC55317.1 percent identity 99 in

405 aa"

/product="transposase"

/protein_id="BAD72028.1"

**A** 0.91 0.364 0.96 0.381 0.93 0.393 0.82 0.442 0.066 283 86.93 250724

CDS complement(249870..250724)

/gene="TTHB245"

/inference="non-experimental evidence, no additional

details recorded"

/note="similar to PDB:1V26 percent identity 44 in 291 aa"

/product="conserved hypothetical protein"

/protein_id="BAD72041.1"

**A** 1.01 0.609 1.04 0.588 1.03 0.593 0.94 0.650 0.176 93 77.42 256540

CDS 256540..256824

/gene="TTHB251"

/inference="non-experimental evidence, no additional

details recorded"

/note="similar to DBJ:BAC51672.1 percent identity 48 in 74

aa"

/product="ABC transporter, periplasmic solute-binding

protein-related protein"

/protein_id="BAD72047.1"
