# Supplementary material for: Sequence of the hyperplastic genome of the naturally competent Thermus scotoductus SA-01
Source: BMC Genomics. 2011 Nov 24;12:577. doi: 10.1186/1471-2164-12-577 (PMC3235269; doi:10.1186/1471-2164-12-577)
Supplement: Additional file 5 — Table S5. List of alien genes: Thermus thermophilus HB27 chromosome and megaplasmid. Contains a list of putative alien gens in Thermus thermophilusHB8 as determined by codon bias relative to all genes using Karlin's codon bias method. [file 1471-2164-12-577-S5.DOC]

**LIST OF ALIEN GENES: *Thermus thermophilus* HB27 chromosome and megaplasmid.**

**Determined by codon bias relative to all genes and selected other standards**

Standards: chromosome1CDS.cbRAll

chromosome1CDS.cbRRP

chromosome1CDS.cbRCH

chromosome1CDS.cbRTF

Number of genes: 1893

Criteria: all biases > threshold depending on gene length:

0.4223(100) 0.3813(150) 0.3294(250) 0.2875(400) 0.2531(600)

Eg(Standard) = Bias(All)/Bias(Standard)

Eg = Bias(All)/[0.5*Bias(RP)+0.25*Bias(CH)+0.25*Bias(TF)]

ALIEN GENES:

Eg B(all) EgRP B(RP) EgCH B(CH) EgTF B(TF) Ag Length S3 Position

**A** 0.90 0.576 0.93 0.616 0.87 0.658 0.86 0.673 0.193 104 69.23 39291

CDS 39291..39608

/locus_tag="TT_C0042"

/old_locus_tag="TTC0042"

/product="hypothetical protein"

/protein_id="AAS80390.1"

**A** 0.87 0.407 0.95 0.429 0.79 0.515 0.82 0.497 0.084 191 80.10 68033

CDS complement(67455..68033)

/locus_tag="TT_C0077"

/old_locus_tag="TTC0077"

/product="ribonuclease P protein subunit"

/protein_id="AAS80425.1"

**A** 0.87 0.387 0.95 0.407 0.80 0.485 0.81 0.477 0.125 365 75.62 71652

CDS 71652..72752

/gene="livM"

/locus_tag="TT_C0081"

/old_locus_tag="TTC0081"

/product="branched-chain amino acid transport system

permease protein livM"

/protein_id="AAS80429.1"

**A** 0.82 0.329 0.88 0.374 0.70 0.469 0.86 0.382 0.041 256 87.11 80017

CDS 80017..80790

/locus_tag="TT_C0090"

/old_locus_tag="TTC0090"

/product="hypothetical protein"

/protein_id="AAS80438.1"

**A** 0.92 0.443 0.96 0.460 0.87 0.508 0.90 0.493 0.160 339 91.45 90230

CDS 90230..91252

/gene="appD"

/locus_tag="TT_C0101"

/old_locus_tag="TTC0101"

/product="oligopeptide transport ATP-binding protein appD"

/protein_id="AAS80449.1"

**A** 0.92 0.345 0.98 0.351 0.84 0.409 0.89 0.389 0.056 326 92.02 91249

CDS 91249..92232

/gene="appF"

/locus_tag="TT_C0102"

/old_locus_tag="TTC0102"

/product="oligopeptide transport ATP-binding protein appF"

/protein_id="AAS80450.1"

**A** 1.01 0.472 1.03 0.458 0.99 0.475 0.99 0.475 0.131 230 90.87 117218

CDS 117218..117913

/gene="merR"

/locus_tag="TT_C0132"

/old_locus_tag="TTC0132"

/product="probable transcriptional regulator, merR family"

/protein_id="AAS80480.1"

**A** 0.91 0.335 0.97 0.345 0.81 0.413 0.90 0.374 0.046 320 88.12 141611

CDS complement(140646..141611)

/locus_tag="TT_C0158"

/old_locus_tag="TTC0158"

/product="probable two-component sensor"

/protein_id="AAS80506.1"

**A** 0.97 0.435 1.01 0.433 0.95 0.456 0.91 0.481 0.025 102 90.20 161450

CDS 161450..161761

/locus_tag="TT_C0179"

/old_locus_tag="TTC0179"

/product="hypothetical protein"

/protein_id="AAS80527.1"

**A** 0.91 0.413 0.97 0.428 0.82 0.504 0.89 0.461 0.100 233 79.40 210553

CDS complement(209849..210553)

/locus_tag="TT_C0223"

/old_locus_tag="TTC0223"

/product="hypothetical protein"

/protein_id="AAS80571.1"

**A** 0.83 0.367 0.88 0.417 0.80 0.460 0.76 0.481 0.102 328 80.18 228534

CDS 228534..229523

/locus_tag="TT_C0242"

/old_locus_tag="TTC0242"

/product="biotin synthase"

/protein_id="AAS80590.1"

**A** 0.92 0.595 1.00 0.596 0.85 0.701 0.87 0.685 0.224 123 79.67 253368

CDS complement(252994..253368)

/locus_tag="TT_C0261"

/old_locus_tag="TTC0261"

/product="large-conductance mechanosensitive channel"

/protein_id="AAS80609.1"

**A** 0.94 0.403 0.95 0.424 0.95 0.423 0.92 0.438 0.017 128 84.38 259319

CDS 259319..259705

/locus_tag="TT_C0270"

/old_locus_tag="TTC0270"

/product="probable nucleotidyltransferase"

/protein_id="AAS80618.1"

**A** 0.83 0.318 0.88 0.361 0.80 0.400 0.78 0.408 0.091 541 86.88 262882

CDS complement(261254..262882)

/locus_tag="TT_C0273"

/old_locus_tag="TTC0273"

/product="transposase"

/protein_id="AAS80621.1"

**A** 0.92 0.804 0.96 0.841 0.89 0.908 0.89 0.904 0.547 367 54.77 267457

CDS complement(266351..267457)

/locus_tag="TT_C0277"

/old_locus_tag="TTC0277"

/product="hypothetical protein"

/protein_id="AAS80625.1"

**A** 0.85 0.552 0.88 0.626 0.82 0.669 0.83 0.667 0.245 195 69.74 268067

CDS complement(267477..268067)

/locus_tag="TT_C0278"

/old_locus_tag="TTC0278"

/product="hypothetical protein"

/protein_id="AAS80626.1"

**A** 0.89 0.417 0.94 0.442 0.87 0.480 0.81 0.513 0.131 297 77.44 268989

CDS complement(268093..268989)

/locus_tag="TT_C0279"

/old_locus_tag="TTC0279"

/product="probable glycosyltransferase"

/protein_id="AAS80627.1"

**A** 0.91 0.679 0.94 0.721 0.89 0.765 0.86 0.788 0.419 358 61.45 270055

CDS complement(268979..270055)

/locus_tag="TT_C0280"

/old_locus_tag="TTC0280"

/product="lipopolysaccharide

N-acetylglucosaminyltransferase"

/protein_id="AAS80628.1"

**A** 0.94 0.831 0.96 0.863 0.94 0.884 0.90 0.924 0.583 459 53.81 271397

CDS complement(270015..271397)

/locus_tag="TT_C0281"

/old_locus_tag="TTC0281"

/product="hypothetical protein"

/protein_id="AAS80629.1"

**A** 0.92 0.791 0.95 0.834 0.91 0.873 0.88 0.903 0.573 574 55.57 273097

CDS complement(271370..273097)

/locus_tag="TT_C0282"

/old_locus_tag="TTC0282"

/note="glutamine-hydrolyzing]"

/product="asparagine synthetase"

/protein_id="AAS80630.1"

**A** 0.94 0.724 0.96 0.753 0.92 0.787 0.91 0.799 0.467 417 63.79 274350

CDS complement(273094..274350)

/locus_tag="TT_C0283"

/old_locus_tag="TTC0283"

/product="hypothetical protein"

/protein_id="AAS80631.1"

**A** 0.89 0.296 0.96 0.309 0.78 0.377 0.88 0.338 0.023 370 81.35 275462

CDS complement(274347..275462)

/locus_tag="TT_C0284"

/old_locus_tag="TTC0284"

/product="pleiotropic regulatory protein"

/protein_id="AAS80632.1"

**A** 0.89 0.439 0.95 0.463 0.84 0.524 0.84 0.520 0.173 364 71.98 276556

CDS complement(275459..276556)

/locus_tag="TT_C0285"

/old_locus_tag="TTC0285"

/product="UDP-N-acetylglucosamine 2-epimerase"

/protein_id="AAS80633.1"

**A** 0.83 0.385 0.84 0.457 0.78 0.496 0.86 0.448 0.066 191 82.20 277139

CDS complement(276561..277139)

/locus_tag="TT_C0286"

/old_locus_tag="TTC0286"

/product="acetyltransferase"

/protein_id="AAS80634.1"

**A** 0.87 0.546 0.91 0.599 0.81 0.671 0.83 0.654 0.241 206 81.07 280064

CDS complement(279441..280064)

/locus_tag="TT_C0289"

/old_locus_tag="TTC0289"

/product="transposase"

/protein_id="AAS80637.1"

**A** 0.76 0.370 0.78 0.477 0.72 0.512 0.77 0.482 0.118 311 86.50 304005

CDS 304005..304943

/locus_tag="TT_C0318"

/old_locus_tag="TTC0318"

/product="putative oxidoreductase"

/protein_id="AAS80666.1"

**A** 0.91 0.461 0.94 0.491 0.85 0.544 0.93 0.497 0.056 90 83.33 334865

CDS complement(334590..334865)

/locus_tag="TT_C0348"

/old_locus_tag="TTC0348"

/product="hypothetical conserved protein"

/protein_id="AAS80696.1"

**A** 0.79 0.310 0.86 0.361 0.71 0.433 0.73 0.422 0.104 682 84.02 339638

CDS 339638..341689

/locus_tag="TT_C0354"

/old_locus_tag="TTC0354"

/product="cation-transporting ATPase"

/protein_id="AAS80702.1"

**A** 0.92 0.528 0.99 0.534 0.87 0.608 0.85 0.622 0.281 458 73.14 356874

CDS 356874..358250

/locus_tag="TT_C0372"

/old_locus_tag="TTC0372"

/product="serine protease"

/protein_id="AAS80720.1"

**A** 0.93 0.426 0.97 0.438 0.90 0.472 0.87 0.488 0.111 239 80.33 388313

CDS complement(387591..388313)

/locus_tag="TT_C0400"

/old_locus_tag="TTC0400"

/product="hypothetical protein"

/protein_id="AAS80748.1"

**A** 0.87 0.528 0.89 0.591 0.86 0.614 0.85 0.623 0.272 366 72.13 389315

CDS 389315..390418

/locus_tag="TT_C0402"

/old_locus_tag="TTC0402"

/product="hypothetical conserved protein"

/protein_id="AAS80750.1"

**A** 0.91 0.376 0.97 0.386 0.82 0.461 0.88 0.426 0.063 236 79.66 393908

CDS complement(393195..393908)

/locus_tag="TT_C0405"

/old_locus_tag="TTC0405"

/product="hypothetical protein"

/protein_id="AAS80753.1"

**A** 0.97 0.328 1.06 0.311 0.90 0.364 0.88 0.371 0.040 360 84.72 402015

CDS 402015..403100

/locus_tag="TT_C0414"

/old_locus_tag="TTC0414"

/product="mannitol-binding protein"

/protein_id="AAS80762.1"

**A** 0.79 0.314 0.82 0.382 0.74 0.425 0.77 0.411 0.079 436 85.32 403607

CDS 403607..404920

/locus_tag="TT_C0416"

/old_locus_tag="TTC0416"

/product="tripartite transporter, large subunit"

/protein_id="AAS80764.1"

**A** 0.89 0.497 0.95 0.524 0.81 0.612 0.85 0.583 0.168 177 70.62 413527

CDS complement(412991..413527)

/locus_tag="TT_C0423"

/old_locus_tag="TTC0423"

/product="hypothetical protein"

/protein_id="AAS80771.1"

**A** 0.87 0.409 0.92 0.446 0.84 0.488 0.81 0.508 0.133 314 82.17 431544

CDS 431544..432491

/locus_tag="TT_C0444"

/old_locus_tag="TTC0444"

/product="hypothetical conserved protein"

/protein_id="AAS80792.1"

**A** 0.84 0.318 0.89 0.356 0.76 0.419 0.82 0.389 0.088 527 83.68 434178

CDS complement(432592..434178)

/locus_tag="TT_C0445"

/old_locus_tag="TTC0445"

/product="hypothetical protein"

/protein_id="AAS80793.1"

**A** 0.88 0.556 0.95 0.586 0.81 0.689 0.85 0.657 0.328 492 69.72 436065

CDS complement(434584..436065)

/locus_tag="TT_C0447"

/old_locus_tag="TTC0447"

/product="prephenate dehydrogenase"

/protein_id="AAS80795.1"

HA 1.09 0.480 1.11 0.433 1.11 0.433 1.04 0.461 0.031 91 84.62 504815

CDS complement(504537..504815)

/locus_tag="TT_C0521"

/old_locus_tag="TTC0521"

/product="glutamyl-tRNA(Gln) amidotransferase subunit C"

/protein_id="AAS80869.1"

**A** 0.95 0.351 1.02 0.344 0.92 0.380 0.86 0.409 0.077 402 79.10 550206

CDS complement(548995..550206)

/locus_tag="TT_C0563"

/old_locus_tag="TTC0563"

/product="Na(+)/H(+) antiporter"

/protein_id="AAS80911.1"

**A** 0.86 0.557 0.90 0.616 0.81 0.684 0.82 0.677 0.255 206 81.07 556245

CDS 556245..556868

/locus_tag="TT_C0572"

/old_locus_tag="TTC0572"

/product="probable transposase"

/protein_id="AAS80920.1"

**A** 0.88 0.491 0.94 0.520 0.84 0.584 0.81 0.604 0.220 318 73.27 561038

CDS complement(560079..561038)

/locus_tag="TT_C0576"

/old_locus_tag="TTC0576"

/product="hypothetical protein"

/protein_id="AAS80924.1"

**A** 0.87 0.565 0.92 0.617 0.82 0.689 0.84 0.675 0.260 206 81.07 561682

CDS complement(561059..561682)

/locus_tag="TT_C0577"

/old_locus_tag="TTC0577"

/product="probable transposase"

/protein_id="AAS80925.1"

**A** 0.89 0.612 0.93 0.661 0.86 0.710 0.86 0.713 0.308 220 66.36 642216

CDS 642216..642881

/locus_tag="TT_C0653"

/old_locus_tag="TTC0653"

/product="hypothetical protein"

/protein_id="AAS81001.1"

**A** 0.93 0.558 0.99 0.566 0.87 0.644 0.90 0.620 0.156 93 83.87 642892

CDS 642892..643176

/locus_tag="TT_C0654"

/old_locus_tag="TTC0654"

/product="hypothetical protein"

/protein_id="AAS81002.1"

**A** 1.03 0.512 1.03 0.497 0.98 0.520 1.09 0.471 0.090 110 97.27 646020

CDS 646020..646355

/locus_tag="TT_C0658"

/old_locus_tag="TTC0658"

/product="hypothetical protein"

/protein_id="AAS81006.1"

**A** 0.83 0.487 0.85 0.573 0.75 0.646 0.86 0.568 0.144 131 77.10 649127

CDS 649127..649525

/locus_tag="TT_C0662"

/old_locus_tag="TTC0662"

/product="hypothetical protein"

/protein_id="AAS81010.1"

**A** 0.91 0.584 0.95 0.612 0.89 0.660 0.86 0.682 0.239 154 68.83 651303

CDS 651303..651770

/locus_tag="TT_C0664"

/old_locus_tag="TTC0664"

/product="hypothetical conserved protein"

/protein_id="AAS81012.1"

**A** 0.88 0.298 0.94 0.317 0.79 0.379 0.87 0.341 0.031 390 87.95 651891

CDS 651891..653066

/locus_tag="TT_C0665"

/old_locus_tag="TTC0665"

/product="transposase"

/protein_id="AAS81013.1"

**A** 0.95 0.530 1.02 0.520 0.87 0.608 0.89 0.595 0.209 223 74.44 814451

CDS complement(813777..814451)

/locus_tag="TT_C0834"

/old_locus_tag="TTC0834"

/product="hypothetical conserved protein"

/protein_id="AAS81180.1"

**A** 0.97 0.512 1.01 0.506 0.95 0.537 0.90 0.567 0.112 112 75.89 819183

CDS complement(818842..819183)

/locus_tag="TT_C0840"

/old_locus_tag="TTC0840"

/product="hypothetical protein"

/protein_id="AAS81186.1"

**A** 0.94 0.427 0.99 0.432 0.86 0.497 0.92 0.463 0.077 174 82.76 825282

CDS complement(824755..825282)

/locus_tag="TT_C0848"

/old_locus_tag="TTC0848"

/product="acetyltransferase"

/protein_id="AAS81192.1"

**A** 0.93 0.427 0.95 0.451 0.88 0.483 0.94 0.455 0.086 192 83.85 831932

CDS 831932..832513

/locus_tag="TT_C0855"

/old_locus_tag="TTC0855"

/product="prepilin-like protein"

/protein_id="AAS81199.1"

**A** 0.94 0.609 0.99 0.613 0.88 0.693 0.90 0.678 0.237 130 73.85 835117

CDS 835117..835512

/gene="pilA"

/locus_tag="TT_C0858"

/old_locus_tag="TTC0858"

/product="pilA"

/protein_id="AAS81202.1"

**A** 0.90 0.499 0.94 0.533 0.86 0.582 0.86 0.583 0.121 113 76.99 911417

CDS complement(911073..911417)

/locus_tag="TT_C0930"

/old_locus_tag="TTC0930"

/product="hypothetical protein"

/protein_id="AAS81274.1"

**A** 0.86 0.485 0.89 0.543 0.77 0.632 0.91 0.534 0.113 109 83.49 920967

CDS complement(920635..920967)

/locus_tag="TT_C0941"

/old_locus_tag="TTC0941"

/product="hypothetical protein"

/protein_id="AAS81283.1"

**A** 0.94 0.588 0.97 0.606 0.94 0.627 0.89 0.658 0.303 334 69.16 979831

CDS complement(978824..979831)

/locus_tag="TT_C0999"

/old_locus_tag="TTC0999"

/product="hypothetical conserved protein"

/protein_id="AAS81341.1"

**A** 0.87 0.268 0.85 0.316 0.84 0.318 0.95 0.281 0.024 547 90.86 1035075

CDS 1035075..1036721

/locus_tag="TT_C1065"

/old_locus_tag="TTC1065"

/product="long-chain-fatty-acid-CoA ligase"

/protein_id="AAS81407.1"

**A** 0.97 0.445 1.01 0.440 0.93 0.477 0.92 0.481 0.092 185 76.76 1057413

CDS 1057413..1057973

/locus_tag="TT_C1088"

/old_locus_tag="TTC1088"

/product="protease I"

/protein_id="AAS81430.1"

**A** 0.96 0.298 0.92 0.324 0.93 0.320 1.08 0.276 0.042 559 89.45 1066141

CDS 1066141..1067823

/locus_tag="TT_C1099"

/old_locus_tag="TTC1099"

/product="long-chain-fatty-acid-CoA ligase"

/protein_id="AAS81441.1"

**A** 0.96 0.409 1.04 0.392 0.89 0.459 0.90 0.452 0.031 139 89.93 1086573

CDS complement(1086151..1086573)

/locus_tag="TT_C1117"

/old_locus_tag="TTC1117"

/product="thioredoxin"

/protein_id="AAS81459.1"

**A** 0.83 0.387 0.87 0.447 0.81 0.480 0.79 0.490 0.087 224 87.50 1112972

CDS complement(1112295..1112972)

/gene="hemD"

/locus_tag="TT_C1143"

/old_locus_tag="TTC1143"

/product="putative uroporphyrinogen-III synthase hemD"

/protein_id="AAS81485.1"

**A** 0.93 0.564 1.01 0.558 0.85 0.665 0.89 0.635 0.161 89 83.15 1115440

CDS complement(1115168..1115440)

/locus_tag="TT_C1148"

/old_locus_tag="TTC1148"

/product="putative nucleotidyltransferase"

/protein_id="AAS81490.1"

**A** 0.83 0.309 0.87 0.355 0.75 0.412 0.83 0.373 0.079 527 85.01 1143486

CDS complement(1141900..1143486)

/locus_tag="TT_C1174"

/old_locus_tag="TTC1174"

/product="conserved hypothetical protein"

/protein_id="AAS81516.1"

**A** 0.95 0.766 0.98 0.781 0.94 0.814 0.90 0.851 0.465 263 59.32 1180702

CDS complement(1179908..1180702)

/locus_tag="TT_C1217"

/old_locus_tag="TTC1217"

/product="hypothetical protein"

/protein_id="AAS81559.1"

**A** 0.92 0.469 0.94 0.498 0.90 0.521 0.89 0.524 0.078 110 77.27 1240055

CDS complement(1239720..1240055)

/locus_tag="TT_C1284"

/old_locus_tag="TTC1284"

/product="nucleotidyltransferase"

/protein_id="AAS81626.1"

**A** 0.92 0.535 0.94 0.569 0.91 0.586 0.90 0.597 0.132 94 80.85 1240339

CDS complement(1240052..1240339)

/locus_tag="TT_C1285"

/old_locus_tag="TTC1285"

/product="nucleotidyltransferase"

/protein_id="AAS81627.1"

**A** 0.88 0.407 0.96 0.423 0.78 0.524 0.83 0.493 0.059 146 82.88 1247401

CDS complement(1246958..1247401)

/locus_tag="TT_C1292"

/old_locus_tag="TTC1292"

/product="hypothetical protein"

/protein_id="AAS81634.1"

**A** 0.99 0.706 1.02 0.695 0.97 0.725 0.96 0.737 0.426 405 84.69 1268186

CDS complement(1266966..1268186)

/locus_tag="TT_C1330"

/old_locus_tag="TTC1330"

/product="elongation factor Tu"

/protein_id="AAS81672.1"

**A** 0.91 0.424 0.93 0.455 0.88 0.484 0.89 0.476 0.077 171 78.36 1295659

CDS 1295659..1296177

/locus_tag="TT_C1362"

/old_locus_tag="TTC1362"

/product="hypothetical conserved protein"

/protein_id="AAS81704.1"

**A** 0.89 0.509 0.92 0.553 0.85 0.597 0.87 0.588 0.107 82 78.05 1297046

CDS 1297046..1297297

/locus_tag="TT_C1364"

/old_locus_tag="TTC1364"

/product="hypothetical protein"

/protein_id="AAS81706.1"

**A** 0.92 0.584 0.97 0.600 0.88 0.661 0.88 0.667 0.344 507 67.26 1351412

CDS 1351412..1352938

/locus_tag="TT_C1428"

/old_locus_tag="TTC1428"

/product="hypothetical protein"

/protein_id="AAS81770.1"

**A** 0.95 0.511 1.03 0.497 0.90 0.571 0.87 0.590 0.239 380 74.47 1352928

CDS 1352928..1354073

/locus_tag="TT_C1429"

/old_locus_tag="TTC1429"

/product="hypothetical protein"

/protein_id="AAS81771.1"

**A** 0.92 0.465 1.00 0.463 0.85 0.546 0.85 0.550 0.239 610 75.74 1354087

CDS 1354087..1355922

/locus_tag="TT_C1430"

/old_locus_tag="TTC1430"

/product="hypothetical protein"

/protein_id="AAS81772.1"

HA 1.09 0.509 1.13 0.450 1.06 0.483 1.04 0.488 0.072 104 85.58 1355993

CDS 1355993..1356310

/locus_tag="TT_C1431"

/old_locus_tag="TTC1431"

/product="transposase"

/protein_id="AAS81773.1"

**A** 0.89 0.401 0.97 0.414 0.85 0.474 0.82 0.492 0.139 384 76.56 1357495

CDS complement(1356338..1357495)

/locus_tag="TT_C1432"

/old_locus_tag="TTC1432"

/product="transposase"

/protein_id="AAS81774.1"

**A** 0.86 0.547 0.90 0.611 0.82 0.665 0.84 0.649 0.254 229 84.72 1370910

CDS 1370910..1371599

/locus_tag="TT_C1450"

/old_locus_tag="TTC1450"

/product="hypothetical conserved protein"

/protein_id="AAS81792.1"

**A** 0.90 0.370 0.96 0.385 0.84 0.439 0.86 0.431 0.098 371 78.71 1395245

CDS 1395245..1396363

/locus_tag="TT_C1472"

/old_locus_tag="TTC1472"

/product="hypothetical protein"

/protein_id="AAS81814.1"

**A** 0.75 0.328 0.81 0.406 0.66 0.497 0.76 0.430 0.074 313 87.86 1419652

CDS 1419652..1420596

/locus_tag="TT_C1495"

/old_locus_tag="TTC1495"

/product="hypothetical protein"

/protein_id="AAS81837.1"

**A** 0.86 0.495 0.92 0.538 0.80 0.619 0.81 0.612 0.223 287 71.08 1421978

CDS 1421978..1422844

/locus_tag="TT_C1497"

/old_locus_tag="TTC1497"

/product="hypothetical protein"

/protein_id="AAS81839.1"

**A** 0.83 0.287 0.95 0.300 0.72 0.399 0.76 0.375 0.042 443 82.62 1424383

CDS 1424383..1425717

/locus_tag="TT_C1499"

/old_locus_tag="TTC1499"

/product="hypothetical protein"

/protein_id="AAS81841.1"

**A** 0.86 0.547 0.90 0.611 0.82 0.665 0.84 0.649 0.254 229 84.72 1534040

CDS complement(1533351..1534040)

/locus_tag="TT_C1617"

/old_locus_tag="TTC1617"

/product="hypothetical conserved protein"

/protein_id="AAS81959.1"

**A** 0.89 0.529 0.94 0.561 0.86 0.617 0.84 0.633 0.209 197 71.57 1595709

CDS complement(1595113..1595709)

/locus_tag="TT_C1680"

/old_locus_tag="TTC1680"

/product="hypothetical protein"

/protein_id="AAS82022.1"

**A** 0.99 0.711 1.02 0.698 0.98 0.728 0.96 0.742 0.430 405 84.44 1649913

CDS 1649913..1651133

/locus_tag="TT_C1734"

/old_locus_tag="TTC1734"

/product="elongation factor Tu"

/protein_id="AAS82076.1"

**A** 0.78 0.421 0.76 0.551 0.70 0.604 0.90 0.467 0.074 118 88.98 1659288

CDS 1659288..1659647

/locus_tag="TT_C1748"

/old_locus_tag="TTC1748"

/product="hypothetical protein"

/protein_id="AAS82090.1"

**A** 0.82 0.451 0.87 0.522 0.77 0.588 0.78 0.577 0.227 442 75.79 1660594

CDS 1660594..1661925

/locus_tag="TT_C1750"

/old_locus_tag="TTC1750"

/product="methyltransferase"

/protein_id="AAS82092.1"

**A** 0.84 0.300 0.92 0.326 0.81 0.372 0.76 0.397 0.034 363 81.27 1690695

CDS complement(1689601..1690695)

/locus_tag="TT_C1784"

/old_locus_tag="TTC1784"

/product="hypothetical conserved protein"

/protein_id="AAS82126.1"

**A** 0.85 0.341 0.89 0.382 0.79 0.430 0.85 0.401 0.055 293 87.37 1702396

CDS 1702396..1703280

/locus_tag="TT_C1795"

/old_locus_tag="TTC1795"

/product="dihydropteroate synthase"

/protein_id="AAS82137.1"

**A** 0.84 0.386 0.83 0.463 0.83 0.466 0.87 0.441 0.037 147 81.63 1711762

CDS complement(1711316..1711762)

/locus_tag="TT_C1804"

/old_locus_tag="TTC1804"

/product="hypothetical conserved protein"

/protein_id="AAS82146.1"

**A** 0.99 0.561 1.03 0.545 1.00 0.561 0.92 0.611 0.157 115 75.65 1744602

CDS 1744602..1744952

/locus_tag="TT_C1836"

/old_locus_tag="TTC1836"

/product="hypothetical protein"

/protein_id="AAS82178.1"

**A** 0.86 0.404 0.90 0.450 0.80 0.507 0.87 0.465 0.040 129 83.72 1747298

CDS 1747298..1747690

/locus_tag="TT_C1840"

/old_locus_tag="TTC1840"

/product="hypothetical protein"

/protein_id="AAS82182.1"

**A** 0.91 0.447 0.95 0.469 0.85 0.529 0.89 0.502 0.141 245 76.33 1747687

CDS 1747687..1748427

/locus_tag="TT_C1841"

/old_locus_tag="TTC1841"

/product="hypothetical protein"

/protein_id="AAS82183.1"

**A** 0.84 0.429 0.88 0.487 0.79 0.541 0.82 0.522 0.151 278 73.38 1748424

CDS 1748424..1749263

/locus_tag="TT_C1842"

/old_locus_tag="TTC1842"

/product="hypothetical protein"

/protein_id="AAS82184.1"

**A** 0.91 0.295 0.97 0.303 0.81 0.363 0.89 0.330 0.024 391 88.49 1779757

CDS complement(1778579..1779757)

/locus_tag="TT_C1876"

/old_locus_tag="TTC1876"

/product="DNA integration/recombination/invertion protein"

/protein_id="AAS82218.1"

**A** 1.01 0.435 1.08 0.404 0.96 0.454 0.93 0.467 0.185 1120 77.50 1780134

CDS 1780134..1783499

/locus_tag="TT_C1877"

/old_locus_tag="TTC1877"

/product="hypothetical conserved protein"

/protein_id="AAS82219.1"

**A** 0.95 0.589 1.02 0.577 0.90 0.658 0.89 0.660 0.316 376 70.48 1783504

CDS 1783504..1784637

/locus_tag="TT_C1878"

/old_locus_tag="TTC1878"

/product="hypothetical protein"

/protein_id="AAS82220.1"

**A** 0.97 0.648 1.00 0.646 0.95 0.681 0.92 0.706 0.404 567 68.08 1784640

CDS 1784640..1786346

/locus_tag="TT_C1879"

/old_locus_tag="TTC1879"

/product="hypothetical cytosolic protein"

/protein_id="AAS82221.1"

**A** 0.93 0.986 0.96 1.030 0.93 1.065 0.90 1.101 0.740 409 46.94 1786343

CDS 1786343..1787575

/locus_tag="TT_C1880"

/old_locus_tag="TTC1880"

/product="hypothetical protein"

/protein_id="AAS82222.1"

**A** 0.82 0.306 0.88 0.347 0.77 0.395 0.77 0.397 0.080 541 87.43 1789366

CDS complement(1787738..1789366)

/locus_tag="TT_C1881"

/old_locus_tag="TTC1881"

/product="transposase"

/protein_id="AAS82223.1"

**A** 0.93 0.432 0.97 0.445 0.92 0.468 0.86 0.504 0.118 240 78.75 1790230

CDS 1790230..1790955

/locus_tag="TT_C1883"

/old_locus_tag="TTC1883"

/product="hypothetical conserved protein"

/protein_id="AAS82225.1"

**A** 0.92 0.849 0.94 0.907 0.92 0.918 0.89 0.957 0.605 426 52.82 1792446

CDS complement(1791163..1792446)

/locus_tag="TT_C1884"

/old_locus_tag="TTC1884"

/product="hydrolase (HAD superfamily)"

/protein_id="AAS82226.1"

**A** 0.84 0.543 0.90 0.605 0.78 0.694 0.81 0.670 0.254 220 71.36 1851925

CDS 1851925..1852590

/locus_tag="TT_C1943"

/old_locus_tag="TTC1943"

/product="hypothetical protein"

/protein_id="AAS82285.1"

**A** 0.88 0.372 0.93 0.400 0.80 0.463 0.85 0.439 0.092 314 78.03 1878517

CDS complement(1877570..1878517)

/locus_tag="TT_C1973"

/old_locus_tag="TTC1973"

/product="cbs domain proteins"

/protein_id="AAS82315.1"

**A** 0.89 0.457 0.95 0.483 0.86 0.533 0.82 0.557 0.082 117 76.07 1878901

CDS complement(1878545..1878901)

/locus_tag="TT_C1974"

/old_locus_tag="TTC1974"

/product="hypothetical conserved protein"

/protein_id="AAS82316.1"

LIST OF ALIEN GENES

Determined by codon bias relative to all genes and selected other standards

Standards: chromosome2CDS.cbRAll

chromosome2CDS.cbRRP

chromosome2CDS.cbRCH

chromosome2CDS.cbRTF

Number of genes: 211

Criteria: all biases > threshold depending on gene length:

0.4223(100) 0.3813(150) 0.3294(250) 0.2875(400) 0.2531(600)

Eg(Standard) = Bias(All)/Bias(Standard)

Eg = Bias(All)/[0.5*Bias(RP)+0.25*Bias(CH)+0.25*Bias(TF)]

ALIEN GENES:

Eg B(all) EgRP B(RP) EgCH B(CH) EgTF B(TF) Ag Length S3 Position

**A** 0.78 0.393 0.81 0.486 0.71 0.556 0.79 0.495 0.085 176 86.93 4509

CDS 4509..5042

/locus_tag="TT_P0006"

/old_locus_tag="TTP0006"

/product="phosphoglycerate mutase"

/protein_id="AAS82336.1"

**A** 0.74 0.338 0.80 0.424 0.67 0.505 0.73 0.466 0.070 246 86.59 9634

CDS 9634..10377

/locus_tag="TT_P0012"

/old_locus_tag="TTP0012"

/product="precorrin-4 C11-methyltransferase"

/protein_id="AAS82342.1"

**A** 0.94 0.475 1.01 0.470 0.93 0.512 0.83 0.570 0.108 141 80.14 15946

CDS 15946..16371

/locus_tag="TT_P0020"

/old_locus_tag="TTP0020"

/product="hypothetical conserved protein"

/protein_id="AAS82350.1"

**A** 0.86 0.547 0.90 0.611 0.82 0.665 0.84 0.649 0.254 229 84.72 58294

CDS complement(57605..58294)

/locus_tag="TT_P0063"

/old_locus_tag="TTP0063"

/product="hypothetical conserved protein"

/protein_id="AAS82393.1"

**A** 0.87 0.404 0.85 0.476 0.85 0.476 0.93 0.435 0.034 129 83.72 61953

CDS 61953..62345

/locus_tag="TT_P0068"

/old_locus_tag="TTP0068"

/product="hypothetical conserved protein"

/protein_id="AAS82398.1"

**A** 0.87 0.431 0.91 0.472 0.81 0.534 0.86 0.500 0.081 145 83.45 68624

CDS 68624..69061

/locus_tag="TT_P0075"

/old_locus_tag="TTP0075"

/product="hypothetical protein"

/protein_id="AAS82405.1"

**A** 0.87 0.600 0.93 0.642 0.81 0.739 0.82 0.729 0.399 660 68.03 74881

CDS complement(72896..74881)

/locus_tag="TT_P0079"

/old_locus_tag="TTP0079"

/product="hypothetical conserved protein"

/protein_id="AAS82409.1"

**A** 0.87 0.409 0.92 0.446 0.84 0.488 0.81 0.508 0.133 314 82.17 75857

CDS complement(74910..75857)

/locus_tag="TT_P0080"

/old_locus_tag="TTP0080"

/product="hypothetical conserved protein"

/protein_id="AAS82410.1"

**A** 0.88 0.413 0.94 0.439 0.86 0.481 0.80 0.518 0.164 430 73.95 78799

CDS complement(77504..78799)

/locus_tag="TT_P0082"

/old_locus_tag="TTP0082"

/product="hypothetical conserved protein"

/protein_id="AAS82412.1"

**A** 0.86 0.547 0.90 0.611 0.82 0.665 0.84 0.649 0.254 229 84.72 86008

CDS 86008..86697

/locus_tag="TT_P0092"

/old_locus_tag="TTP0092"

/product="hypothetical conserved protein"

/protein_id="AAS82422.1"

**A** 0.91 0.450 0.98 0.461 0.86 0.526 0.86 0.523 0.081 133 90.98 89293

CDS 89293..89697

/locus_tag="TT_P0097"

/old_locus_tag="TTP0097"

/product="hypothetical conserved protein"

/protein_id="AAS82427.1"

**A** 0.86 0.557 0.90 0.616 0.81 0.684 0.82 0.677 0.255 206 81.07 91694

CDS complement(91071..91694)

/locus_tag="TT_P0099"

/old_locus_tag="TTP0099"

/product="probable transposase"

/protein_id="AAS82429.1"

**A** 0.94 0.713 0.99 0.719 0.90 0.794 0.87 0.817 0.364 151 64.24 100311

CDS complement(99853..100311)

/locus_tag="TT_P0108"

/old_locus_tag="TTP0108"

/product="hypothetical protein"

/protein_id="AAS82438.1"

**A** 0.91 0.820 0.95 0.865 0.88 0.934 0.87 0.946 0.501 178 61.24 101591

CDS complement(101055..101591)

/locus_tag="TT_P0109"

/old_locus_tag="TTP0109"

/product="hypothetical protein"

/protein_id="AAS82439.1"

**A** 0.97 0.458 1.03 0.444 0.93 0.493 0.92 0.498 0.105 185 80.54 112661

CDS 112661..113221

/locus_tag="TT_P0121"

/old_locus_tag="TTP0121"

/product="hypothetical conserved protein"

/protein_id="AAS82451.1"

**A** 0.89 0.400 0.93 0.431 0.84 0.476 0.87 0.459 0.061 179 78.77 118415

CDS complement(117873..118415)

/locus_tag="TT_P0126"

/old_locus_tag="TTP0126"

/product="hypothetical protein"

/protein_id="AAS82456.1"

**A** 0.76 0.334 0.82 0.409 0.68 0.491 0.73 0.455 0.071 278 79.14 119251

CDS complement(118412..119251)

/locus_tag="TT_P0127"

/old_locus_tag="TTP0127"

/product="hypothetical protein"

/protein_id="AAS82457.1"

**A** 0.80 0.433 0.84 0.517 0.74 0.581 0.80 0.539 0.117 168 86.31 131056

CDS 131056..131565

/locus_tag="TT_P0131"

/old_locus_tag="TTP0131"

/product="hypothetical protein"

/protein_id="AAS82461.1"

**A** 0.83 0.336 0.90 0.372 0.77 0.438 0.78 0.433 0.122 785 77.58 132238

CDS 132238..134598

/locus_tag="TT_P0132"

/old_locus_tag="TTP0132"

/product="hypothetical conserved protein"

/protein_id="AAS82462.1"

**A** 0.92 0.424 0.97 0.435 0.87 0.487 0.87 0.488 0.106 228 73.68 134608

CDS 134608..135297

/locus_tag="TT_P0133"

/old_locus_tag="TTP0133"

/product="hypothetical conserved protein"

/protein_id="AAS82463.1"

**A** 0.80 0.328 0.86 0.379 0.74 0.442 0.74 0.444 0.122 611 79.05 135206

CDS 135206..137044

/locus_tag="TT_P0134"

/old_locus_tag="TTP0134"

/product="hypothetical conserved protein"

/protein_id="AAS82464.1"

**A** 0.85 0.329 0.93 0.354 0.81 0.407 0.78 0.421 0.069 382 82.20 137044

CDS 137044..138195

/locus_tag="TT_P0135"

/old_locus_tag="TTP0135"

/product="hypothetical conserved protein"

/protein_id="AAS82465.1"

**A** 0.96 0.488 1.02 0.477 0.91 0.537 0.90 0.545 0.094 115 77.39 138185

CDS 138185..138535

/locus_tag="TT_P0136"

/old_locus_tag="TTP0136"

/product="hypothetical conserved protein"

/protein_id="AAS82466.1"

**A** 0.94 0.665 0.98 0.682 0.94 0.709 0.88 0.758 0.314 160 64.38 144917

CDS 144917..145402

/locus_tag="TT_P0141"

/old_locus_tag="TTP0141"

/product="hypothetical protein"

/protein_id="AAS82471.1"

**A** 0.94 0.423 0.96 0.441 0.92 0.462 0.91 0.465 0.049 138 81.16 145799

CDS complement(145383..145799)

/locus_tag="TT_P0142"

/old_locus_tag="TTP0142"

/product="putative plasmid stability protein Y4JK"

/protein_id="AAS82472.1"

**A** 0.96 0.645 0.98 0.655 0.93 0.691 0.93 0.692 0.225 82 75.61 146047

CDS complement(145796..146047)

/locus_tag="TT_P0143"

/old_locus_tag="TTP0143"

/product="hypothetical conserved protein"

/protein_id="AAS82473.1"

**A** 0.84 0.623 0.88 0.705 0.81 0.770 0.81 0.769 0.405 442 64.71 146999

CDS 146999..148330

/locus_tag="TT_P0145"

/old_locus_tag="TTP0145"

/product="hypothetical protein"

/protein_id="AAS82475.1"

**A** 1.04 0.494 1.11 0.445 1.03 0.478 0.93 0.530 0.203 409 77.02 148362

CDS 148362..149594

/locus_tag="TT_P0146"

/old_locus_tag="TTP0146"

/product="hypothetical protein"

/protein_id="AAS82476.1"

**A** 0.98 0.465 1.08 0.429 0.91 0.511 0.88 0.526 0.224 856 81.89 149874

CDS 149874..152447

/locus_tag="TT_P0147"

/old_locus_tag="TTP0147"

/product="hypothetical protein"

/protein_id="AAS82477.1"

**A** 0.78 0.310 0.82 0.377 0.71 0.435 0.79 0.393 0.090 527 82.35 158076

CDS complement(156490..158076)

/locus_tag="TT_P0154"

/old_locus_tag="TTP0154"

/product="hypothetical protein"

/protein_id="AAS82484.1"

**A** 0.86 0.547 0.90 0.611 0.82 0.665 0.84 0.649 0.254 229 84.72 160289

CDS 160289..160978

/locus_tag="TT_P0159"

/old_locus_tag="TTP0159"

/product="hypothetical conserved protein"

/protein_id="AAS82489.1"

**A** 0.84 0.358 0.94 0.379 0.76 0.474 0.77 0.467 0.090 321 77.57 171472

CDS 171472..172440

/locus_tag="TT_P0172"

/old_locus_tag="TTP0172"

/product="diguanylate cyclase/phosphodiesterase domain 1

(GGDEF)"

/protein_id="AAS82502.1"

**A** 0.96 0.409 1.04 0.393 0.90 0.457 0.88 0.467 0.116 328 76.22 181181

CDS complement(180192..181181)

/locus_tag="TT_P0182"

/old_locus_tag="TTP0182"

/product="transposase"

/protein_id="AAS82512.1"

**A** 1.06 0.473 1.08 0.438 1.04 0.456 1.04 0.456 0.039 100 82.00 182341

CDS complement(182036..182341)

/locus_tag="TT_P0184"

/old_locus_tag="TTP0184"

/product="hypothetical protein"

/protein_id="AAS82514.1"

**A** 0.96 0.501 0.98 0.512 0.97 0.515 0.91 0.552 0.086 94 79.79 183597

CDS 183597..183884

/locus_tag="TT_P0186"

/old_locus_tag="TTP0186"

/product="hypothetical conserved protein"

/protein_id="AAS82516.1"

**A** 0.88 0.407 0.96 0.426 0.81 0.505 0.81 0.503 0.116 260 88.08 183962

CDS 183962..184747

/locus_tag="TT_P0187"

/old_locus_tag="TTP0187"

/product="putative transposase"

/protein_id="AAS82517.1"

**A** 0.78 0.305 0.87 0.351 0.71 0.430 0.72 0.423 0.100 765 79.48 195797

CDS 195797..198097

/locus_tag="TT_P0199"

/old_locus_tag="TTP0199"

/product="hypothetical protein"

/protein_id="AAS82529.1"

**A** 0.78 0.345 0.83 0.416 0.74 0.467 0.75 0.458 0.081 303 80.20 198859

CDS 198859..199773

/locus_tag="TT_P0201"

/old_locus_tag="TTP0201"

/product="hypothetical protein"

/protein_id="AAS82531.1"

**A** 0.92 0.333 0.96 0.345 0.87 0.383 0.88 0.377 0.025 263 81.75 202189

CDS 202189..202983

/locus_tag="TT_P0204"

/old_locus_tag="TTP0204"

/product="hypothetical conserved protein"

/protein_id="AAS82532.1"

**A** 0.85 0.458 0.87 0.527 0.80 0.569 0.85 0.541 0.087 109 83.49 204539

CDS complement(204207..204539)

/locus_tag="TT_P0205"

/old_locus_tag="TTP0205"

/product="probable DNA-binding protein"

/protein_id="AAS82533.1"

**A** 0.91 0.461 0.92 0.503 0.87 0.527 0.93 0.495 0.106 156 83.97 207113

CDS 207113..207583

/locus_tag="TT_P0209"

/old_locus_tag="TTP0209"

/product="hypothetical protein"

/protein_id="AAS82537.1"

**A** 0.85 0.327 0.90 0.363 0.81 0.403 0.79 0.414 0.097 541 86.69 209327

CDS complement(207699..209327)

/locus_tag="TT_P0210"

/old_locus_tag="TTP0210"

/product="transposase"

/protein_id="AAS82538.1"

**A** 0.90 0.851 0.93 0.919 0.87 0.977 0.86 0.986 0.489 106 62.26 213009

CDS 213009..213329

/locus_tag="TT_P0214"

/old_locus_tag="TTP0214"

/product="hypothetical protein"

/protein_id="AAS82542.1"

**A** 0.97 0.496 0.97 0.509 0.97 0.510 0.96 0.519 0.070 86 87.21 213997

CDS complement(213734..213997)

/locus_tag="TT_P0215"

/old_locus_tag="TTP0215"

/product="hypothetical protein"

/protein_id="AAS82543.1"

**A** 0.98 0.549 1.04 0.530 0.95 0.577 0.91 0.602 0.204 201 74.63 214183

CDS 214183..214791

/locus_tag="TT_P0216"

/old_locus_tag="TTP0216"

/product="hypothetical protein"

/protein_id="AAS82544.1"

**A** 0.84 0.469 0.90 0.522 0.74 0.631 0.83 0.567 0.150 169 72.19 214819

CDS 214819..215331

/locus_tag="TT_P0217"

/old_locus_tag="TTP0217"

/product="hypothetical conserved protein"

/protein_id="AAS82545.1"

**A** 0.92 0.619 1.01 0.613 0.83 0.744 0.86 0.717 0.246 116 75.86 224924

CDS 224924..225277

/locus_tag="TT_P0224"

/old_locus_tag="TTP0224"

/product="hypothetical protein"

/protein_id="AAS82552.1"
