# Supplementary material for: Sequence of the hyperplastic genome of the naturally competent Thermus scotoductus SA-01
Source: BMC Genomics. 2011 Nov 24;12:577. doi: 10.1186/1471-2164-12-577 (PMC3235269; doi:10.1186/1471-2164-12-577)
Supplement: Additional file 7 — Figure S1. Part of Island 5 showing the DNA segment acquired by T. scotoductus SA01 from Meiothermus ruber. Illustrates the gene cluster for phenolic metabolism of SA-01 which was acquired from Meiothermus ruber. [file 1471-2164-12-577-S7.DOC]

**Figure S1.** Part of Island 5 showing the DNA segment acquired by *T. scotoductus* SA01 from *Meiothermus ruber.*  TSC_C11390 to TSC_C11440 (MRUB_2681 to MRUB_2676) : catechol 2,4 dioxygenase; GntR family transcriptional regulator; conserved hypothetical protein; LmbE family protein, dimethylmenaquinone methyltransferase, 6-phosphogluconate dehydrogenase.
TSC_C11450 to TSC_C11520 (MRUB_2682 to MRUB_2689): P-hydroxybenzoate hydroxylase; dihydrodiol dehydrogenase; 3-phenylpropionate dioxygenase, subunit beta; biphenyl dioxygenase, subunit alpha; ABC transporter ATP-binding/permease protein; membrane spanning protein; inner-membrane translocator; extracellular ligand-binding receptor.
