# Supplementary material for: Sequence of the hyperplastic genome of the naturally competent Thermus scotoductus SA-01
Source: BMC Genomics. 2011 Nov 24;12:577. doi: 10.1186/1471-2164-12-577 (PMC3235269; doi:10.1186/1471-2164-12-577)
Supplement: Additional file 8 — Figure S2. Identification of transformants as T. scotoductus SA1 derivatives. Illustrates that the pMKNor transformed cells were indeed SA-01 and not a contaminant. [file 1471-2164-12-577-S8.DOC]

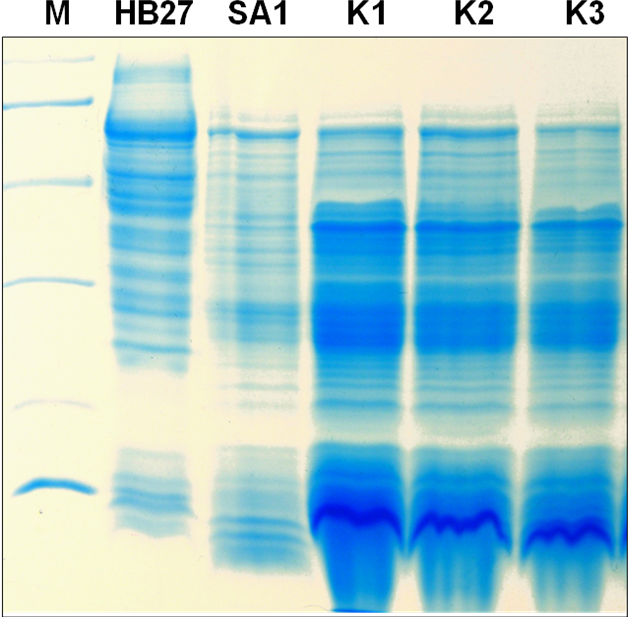

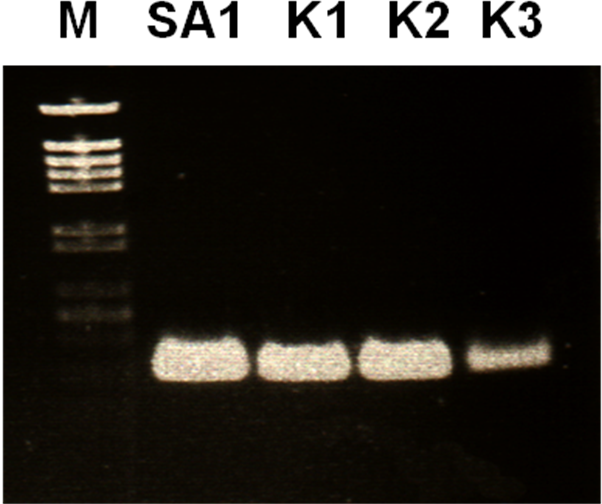


**Figure S2. Identification of transformants as *T. scotoductu*s SA1 derivatives.** A) SDS-PAGE of total proteins from *T. thermophilus* HB27 (HB27), *T. scotoductus* SA01 (SA1), and three SA01 colonies (K1-K3) transformed with pMKNor. B) Positive identification by PCR of a 304 bp fragment of the *nirK* gene (white arrowhead), specific for *T. scotoductus* SA01. M: Molecular weight markers. Proteins (arrowheads): 177, 118, 75, 51, 39, 26 kDa.
